# Health-motivated taxes on red and processed meat: A modelling study on optimal tax levels and associated health impacts

**DOI:** 10.1371/journal.pone.0204139

**Published:** 2018-11-06

**Authors:** Marco Springmann, Daniel Mason-D’Croz, Sherman Robinson, Keith Wiebe, H. Charles J. Godfray, Mike Rayner, Peter Scarborough

**Affiliations:** 1 Oxford Martin Programme on the Future of Food, University of Oxford, Oxford, United Kingdom; 2 Centre on Population Approaches for Non-Communicable Disease Prevention, Nuffield Department of Population Health, University of Oxford, Oxford, United Kingdom; 3 Environment and Production Technology Division, International Food Policy Research Institute (IFPRI), Washington, DC, United States of America; 4 Agriculture and Food, Commonwealth Scientific and Industrial Research Organisation (CSIRO), St Lucia, Queensland, Australia; 5 Department of Zoology, University of Oxford, Oxford, United Kingdom; SOAS, University of London, UNITED KINGDOM

## Abstract

**Background:**

The consumption of red and processed meat has been associated with increased mortality from chronic diseases, and as a result, it has been classified by the World Health Organization as carcinogenic (processed meat) and probably carcinogenic (red meat) to humans. One policy response is to regulate red and processed meat consumption similar to other carcinogens and foods of public health concerns. Here we describe a market-based approach of taxing red and processed meat according to its health impacts.

**Methods:**

We calculated economically optimal tax levels for 149 world regions that would account for (internalize) the health costs associated with ill-health from red and processed meat consumption, and we used a coupled modelling framework to estimate the impacts of optimal taxation on consumption, health costs, and non-communicable disease mortality. Health impacts were estimated using a global comparative risk assessment framework, and economic responses were estimated using international data on health costs, prices, and price elasticities.

**Findings:**

The health-related costs to society attributable to red and processed meat consumption in 2020 amounted to USD 285 billion (sensitivity intervals based on epidemiological uncertainty (SI), 93–431), three quarters of which were due to processed meat consumption. Under optimal taxation, prices for processed meat increased by 25% on average, ranging from 1% in low-income countries to over 100% in high-income countries, and prices for red meat increased by 4%, ranging from 0.2% to over 20%. Consumption of processed meat decreased by 16% on average, ranging from 1% to 25%, whilst red meat consumption remained stable as substitution for processed meat compensated price-related reductions. The number of deaths attributable to red and processed meat consumption decreased by 9% (222,000; SI, 38,000–357,000), and attributable health costs decreased by 14% (USD 41 billion; SI, 10–57) globally, in each case with greatest reductions in high and middle-income countries.

**Interpretation:**

Including the social health cost of red and processed meat consumption in the price of red and processed meat could lead to significant health and environmental benefits, in particular in high and middle-income countries. The optimal tax levels estimated in this study are context-specific and can complement the simple rules of thumb currently used for setting health-motivated tax levels.

## Introduction

The consumption of red and processed meat exceeds recommended levels in most high and middle-income countries and has been associated with a range of negative health and environmental impacts [1,2]. In 2015, the cancer agency of the World Health Organization, the International Agency for Research on Cancer (IARC), classified the consumption of red meat, which includes beef, lamb, and pork, as carcinogenic to humans if eaten in processed form, and as probably carcinogenic if eaten unprocessed [3]. In addition to being linked with cancer, the consumption of red and processed meat has also been associated with increased rates of coronary heart disease[4], stroke [5], type 2 diabetes mellitus [6], and overall mortality [7,8]. Those impacts and the IARC’s classification raise the question whether the consumption of red and processed meat should be regulated similar to other carcinogens or to other foods of public health concern, such as sugary drinks [9].

Market-based approaches to regulation have gained popularity in public health research and the public debate. In particular health-motivated taxes have been widely discussed [10–12], and implemented in some countries, e.g. for sugar-sweetened beverages [9,13], and saturated fats [14]. The tax levels discussed or implemented have mostly been based on practical considerations on their likely impact. However, from an economic perspective, health-motivated taxes are so-called Pigouvian taxes whose purpose it is to correct for the unintended and previously unaccounted consequences to society of an economic activity (in this case, the negative health impacts associated with red and processed meat consumption) by incorporating the cost of those consequences into the price of the activity or good [9,15,16]. Thus, the economically optimal tax level of a health-motivated Pigouvian tax is determined such that market prices include the marginal health costs of consumption, i.e. the cost of treating the health conditions that are associated with one additional serving of the good in question.

Here we provide estimates of the health costs to society and optimal tax levels for red and processed meat for all major world regions, and we estimate the impacts that health-motivated taxation of red and processed meat could have on food consumption, and mortality from diet-related, non-communicable diseases. In our analysis, we treated red meat and processed meat as separate risk factors, and estimated their health burden and health-motivated taxes individually and when combined. We assumed the risk associations between red and processed meat and diet-related diseases as causal based on the existence of plausible pathways, mechanistic evidence, and dose-response relationships (see section A1 in S1 File) [3,6,17–19]. We accounted for changes between red meat consumption and processed meat consumption as a result of differentiated taxation, but also for impacts on other food groups that are considered substitutes, such as poultry, or complements, such as vegetable oils. We focus on the year 2020 as a possible future year for implementation, and we considered other implementation dates (2010 and 2050) in sensitivity analyses.

## Methods

**Fig 1:**
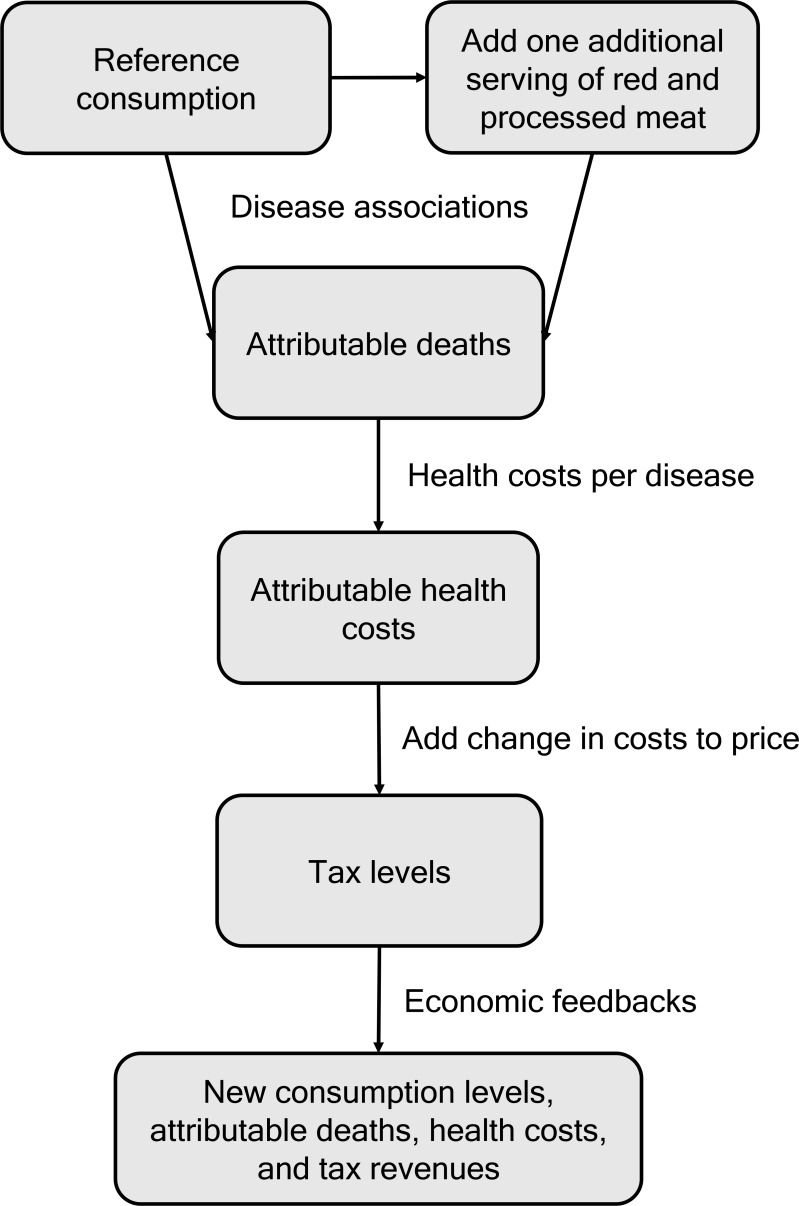
Schematic of algorithm used to calculate optimal tax levels for red and processed meat based on the marginal health costs associated with red and processed meat consumption.

We used a coupled modelling framework to calculate optimal tax levels for red and processed meat and the associated health and climate change impacts in the year 2020 for 149 world regions (Fig 1). Our calculation included several steps. First, we estimated the health impacts associated with the current and projected consumption levels of red and processed meat. Second, we estimated the health costs associated with those health impacts. Third, we repeated that calculation for a scenario in which we increased red and processed meat consumption by a marginal increase which we take to be one additional serving per day in each region. (Note that we are interested in the change in mortality and health costs per marginal increase in consumption. Because the dose-response functions we use are linear and we divide over the marginal increase when levying the damage costs on baseline prices, it does not matter what we define as marginal.) Fourth, we calculated the marginal health costs of red and processed meat consumption by subtracting the cost estimates of the two scenarios. Fifth, we levied the marginal health costs per marginal change in consumption onto the initial market prices of red and processed meat in each region, and calculated the impacts of those price changes on consumption levels, health impacts, and health costs.

For calculating the health impacts associated with red and processed meat consumption, we used a global comparative risk assessment framework [20]. We estimated the mortality burden attributable to changes in the consumption of red and processed meat by calculating population attributable fractions (PAFs) which represent the proportions of disease cases that are attributable to the risk exposure and that would be avoided due to changes in risk exposure, respectively [21–23]. The disease states included coronary heart disease (CHD), stroke, colorectal cancer, and type 2 diabetes mellitus (T2DM). There are indications that red and processed meat consumption increases the risk for other cancers and cardiovascular diseases [24–26]. In a sensitivity analysis, we therefore adopted broader risk associations of red and processed meat consumption with total cancer and cardiovascular diseases in general (Appendix A1 in S1 File) [24,25]. Cause-specific mortality rates and population numbers were adopted from data reported by the Global Burden of Disease project and projected forward using data from the United Nations Population Division. We treated red and processed meat consumption as two separate risk factors, and adopted the relevant relative risk parameters describing the association between red and processed meat consumption and mortality from meta-analyses of prospective cohort studies (Table A1 in S1 File) [19,4,6,5]. For calculating the joint risk of red and processed meat consumption, we combined each PAF mutiplicatively [21–23]. Given that the diseases included in the modelling framework predominantly affect adults, we focused on the health implications for individuals aged 20 and older. In a sensitivity analysis, we estimated the impacts that tax-related changes in food consumption could have on weight distributions and weight-related mortality by using derived relationships between body mass index and food availability (Table A2 in S1 File) [20].

For estimating the health costs associated with changes in mortality, we adopted cost-of-illness (CoI) estimates and used a cost transfer method to estimate the costs of illness in different parts of the world and in different years (section A2 in S1 File) [1]. We based our cost-of-illness estimates for CHD, stroke, and cancer on a comparative assessment of the economic burden of CVD [27,28] and cancer [29] across the European Union which included direct costs (healthcare expenditure, health service utilization, expenditure on medication) and indirect costs (opportunity costs of informal care, productivity costs due to mortality and morbidity). We calculated costs per death based on mortality statistics [28], and estimated the costs per death by disease in other regions and years by scaling the EU base values by the ratio of health expenditure per capita for direct costs, and by the ratio of GDP per capita (adjusted for purchasing power parity) for indirect costs. Productivity losses due to morbidity and mortality, which are a part of the indirect costs, were only included for deaths occurring among those of working age which we took to be below 65 years in all regions, in line with other assessments [29]. For the CoI analysis related to diabetes, we adopted country-specific cost estimates [30], and to avoid double-counting of complications related to cardiovascular diseases, adjusted those for the incremental cost component specifically attributable to diabetes [31,32]. No data was available to estimate indirect costs for T2DM. Where possible, we included both direct and indirect costs in our analysis in order to account for the full health costs of red meat consumption to society, and we explored the relative contributions of direct and indirect costs to the final estimates in a sensitivity analysis. On average, indirect costs represented half to two thirds of the total cost of illness for CHD, stroke, and cancer (Table A3 in S1 File).

For estimating the consumption feedbacks of levying taxes on red and processed meat, we used a global agriculture-economic model, the International Model for Policy Analysis of Agricultural Commodities and Trade (IMPACT) [33], and adjusted it to account for differences between red and processed meat. The IMPACT model is based on a global partial equilibrium multi-market model of agricultural production, demand, trade, and prices (section A3 in S1 File). For our analysis, we adopted IMPACT data on current and future food availability, consumer prices, and on own and cross-price elasticities that determine how the demand of a commodity and related commodities, such as other types of meat, changes when its price changes [34]. To obtain a better proxy for food consumption, we adjusted food availability data for waste at the consumption level using regional estimates from the Food and Agriculture Organization of the United Nations (FAO) (section A4 in S1 File) [35], and we disaggregated total red meat consumption into processed and unprocessed components using compositional data from the Global Dietary Database [2]. Processed meat is generally defined as any meat preserved by salting, curing, smoking, or by adding chemical preservatives, including bacon, sausages, salami, hot dogs and processed deli meats. It can also include processed white meat, but because we disaggregate processed meat from total red meat, we only include processed red meat from beef, lamb and pork in our analysis. We treated red meat and processed meat as substitutes, and used the same cross-price elasticities that describe the substitution of different types of meat (e.g. between beef and poultry). Processed meats are generally cheaper than non-processed meat, because of the quality of the parts of meats used. In our main scenario, we used a price wedge between processed and unprocessed meats of 15%, which is in line with the average price difference over the last five years in the UK [36], and we tested price wedges of zero and 30% in a sensitivity analysis (Tables A27-A28 in S1 File). All monetary data were converted to the value of the US dollar in 2010 by using changes in the consumer price index by region based on data from the International Monetary Fund.

In our uncertainty analysis, we accounted for epidemiological and economic uncertainties. In our analysis, the main source of epidemiological uncertainty is related to the relative risk estimates used for calculating health impacts, and the main source of economic uncertainty is related to the projections of health care-related costs for each region. In each case, we recalculated the endpoints of our analysis (tax levels, consumption changes, health impacts) by using the low and high values of the 95% confidence interval of relative risk estimates, and the standard deviation of health-cost estimates. In the main text, we focus on the epidemiological uncertainty. Using the high and low values of the health-cost estimates resulted in smaller uncertainty intervals than using the high and low values of the epidemiological uncertainty range (Tables A29-A30 in S1 File). We also explored the impacts that changes in price elasticities (which determine consumer responses) have on our estimates. Varying own-price elasticities by 10%, which is in line with estimated confidence intervals [37], also resulted in estimates within the epidemiological uncertainty range (Tables A25-A26 in S1 File).

## Results

### Impacts of optimal taxation

According to our model projections (Table 1), the consumption of red meat was associated with 860,000 (95% confidence interval related to epidemiological uncertainty (SI) 220–1,410,000) deaths globally in the year 2020, and that of processed meat with 1,530,000 (SI, 430–2,470,000) deaths. When assessed together, those represented 4.4% of all projected deaths in the analysis in that year. About two thirds of attributable deaths were due to stroke (for red meat), and coronary heart disease (for processed meat), followed by type-2 diabetes mellitus (14–17%) and colorectal cancer (4–11%). About two thirds of attributable deaths (64%) occurred in middle-income countries, one third (32%) in high-income countries, and a small portion (4%) in low-income countries. The associated costs related to health care amounted to USD 285 billion (SI, 93–431), which represented 0.3% of expected world GDP in that year. More than two thirds of the health costs (69%) fell on high-income countries (due to higher healthcare-related expenditure), a third (30%) on middle-income countries, and a small fraction (0.4%) on low-income countries. Country-level results are listed in Tables A13-A14 in S1 File.

**Table 1:** Impacts of cost-compensating taxation of red and processed meat globally and by regions in different income categories.

Item	Red meat					Processed meat				
	Global	High-income countries	Upper middle-income countries	Lower middle-income countries	Low-income countries	Global	High-income countries	Upper middle-income countries	Lower middle-income countries	Low-income countries
Optimal tax (USD/kg)	0.28	0.94	0.39	0.15	0.02	1.45	4.17	2.41	0.86	0.10
Price before tax (USD/kg)	6.75	4.42	6.05	6.93	8.75	5.74	3.75	5.14	5.89	7.44
Price after tax (USD/kg)	7.03	5.36	6.44	7.08	8.77	7.19	7.93	7.55	6.75	7.54
Price change (%)	4.17	21.36	6.51	2.16	0.23	25.21	111.17	46.85	14.62	1.34
Consumption before tax (g/d)	56.65	94.91	65.97	53.48	25.70	16.52	48.14	25.99	8.88	6.77
Consumption after tax (g/d)	56.76	94.13	66.07	53.86	25.71	13.90	36.06	22.29	8.31	6.69
Consumption change (g/d)	0.11	-0.78	0.09	0.38	0.01	-2.62	-12.09	-3.71	-0.57	-0.08
Consumption change (%)	0.20	-0.82	0.14	0.72	0.04	-15.87	-25.11	-14.25	-6.45	-1.17
Attributable deaths before tax (thousands)	863.06	167.22	124.08	531.38	34.90	1,533.21	604.53	384.96	484.43	55.69
Attributable deaths after tax (thousands)	866.22	165.81	124.84	535.15	34.92	1,298.58	470.21	320.46	449.69	54.81
Change in attributable deaths (thousands)	3.16	-1.41	0.76	3.76	0.02	-234.63	-134.32	-64.50	-34.74	-0.88
Change in attributable deaths (%)	0.37	-0.84	0.61	0.71	0.05	-15.30	-22.22	-16.76	-7.17	-1.58
Health care-related costs before tax (USD billion)	80.74	44.88	10.00	25.17	0.41	216.53	163.34	33.76	18.45	0.76
Health care-related costs after tax (USD billion)	80.58	44.47	10.06	25.35	0.41	173.42	127.97	27.65	16.86	0.74
Change in health care-related costs (USD billion)	-0.16	-0.41	0.06	0.18	0.00	-43.10	-35.37	-6.11	-1.59	-0.02
Change in health care-related costs (%)	-0.20	-0.91	0.63	0.73	0.06	-19.91	-21.66	-18.09	-8.63	-2.25
Tax revenues (USD billion)	69.67	38.19	8.89	21.95	0.37	102.32	71.25	18.16	12.22	0.54

Abbreviations: HIC: high-income countries, UMC: upper middle-income countries, LMC: lower middle-income countries, LIC: low-income countries

Note that the combined effect of red and processed meat is generally lower than the sum of the individual effects as individuals can be affected red and processed meat simultaneously without getting two different types of the same disease. Country-level results are listed in S1 File, and uncertainty intervals and risk aggregates are listed at https://doi.org/10.5287/bodleian:j0n1Jd5rb.

**Fig 2:**
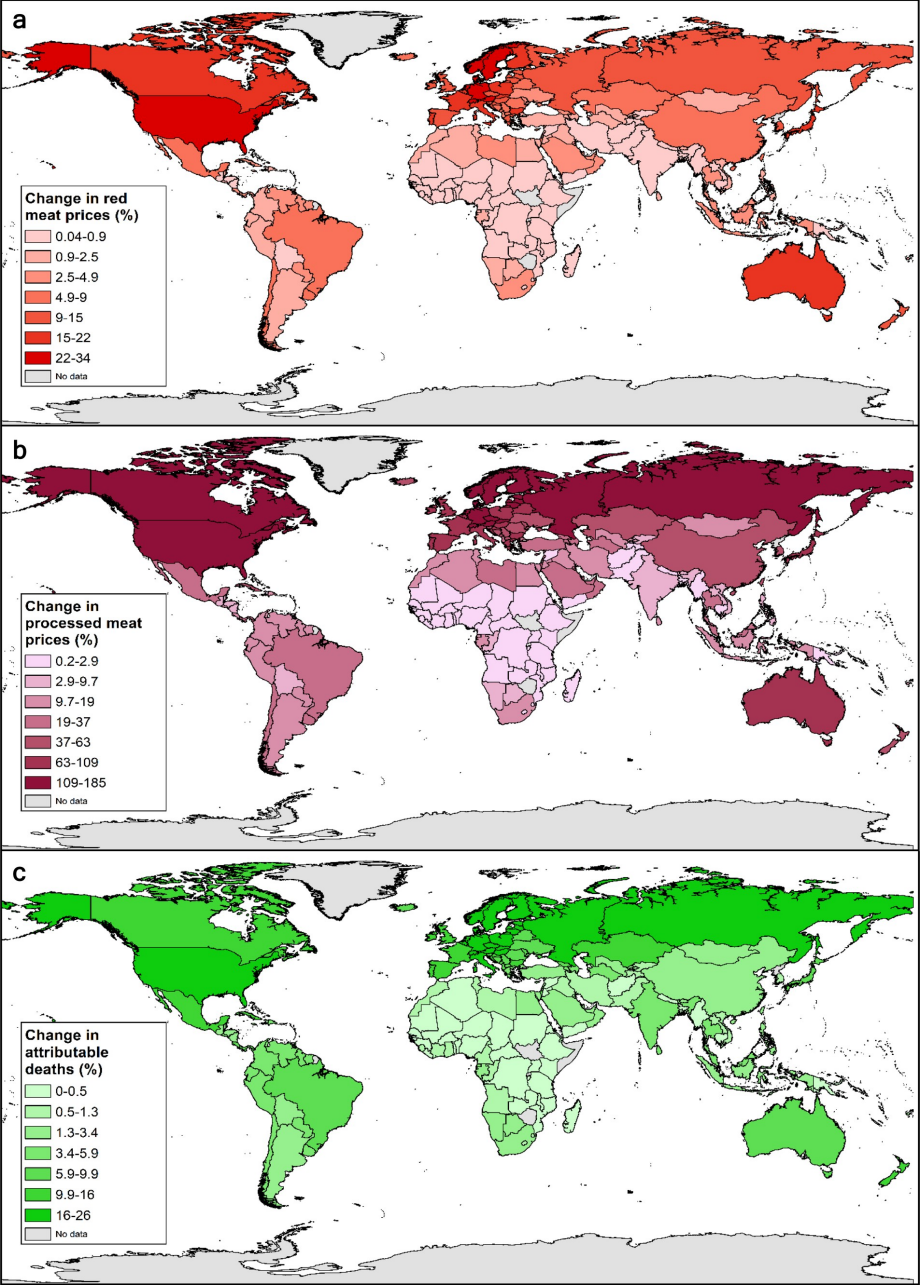
Change in the price of red meat (**a**) and processed meat (**b**) under cost-compensating taxation in relation to attributable health costs (%), change in deaths attributable to red and processed meat consumption (%) (**c**). We produced the figure by mapping our data using ArcGIS (version 10.3.1, Esri Inc.) and its layer for world countries.

Under optimal taxation, the price for one serving of red and processed meat reflects the health costs associated with one additional serving of red and processed meat (Tables A8-A9 in S1 File). Integrating the health costs associated with one serving of red and processed meat into the prices of one serving of red and processed meat increased the price of red meat by 4% (SI, 1–6) on average, ranging from less than 1% in low-income countries to 21% in high-income countries, and the price of processed meat by 25% (SI, 10–32), ranging from 1% in low-income countries to 111% in high-income countries (Table 1). Country-level impacts on prices showed a greater range with price changes of up to 34% for red meat and 185% for processed meat (Fig 2A and 2B; Table A10 in S1 File).

**Fig 3:**
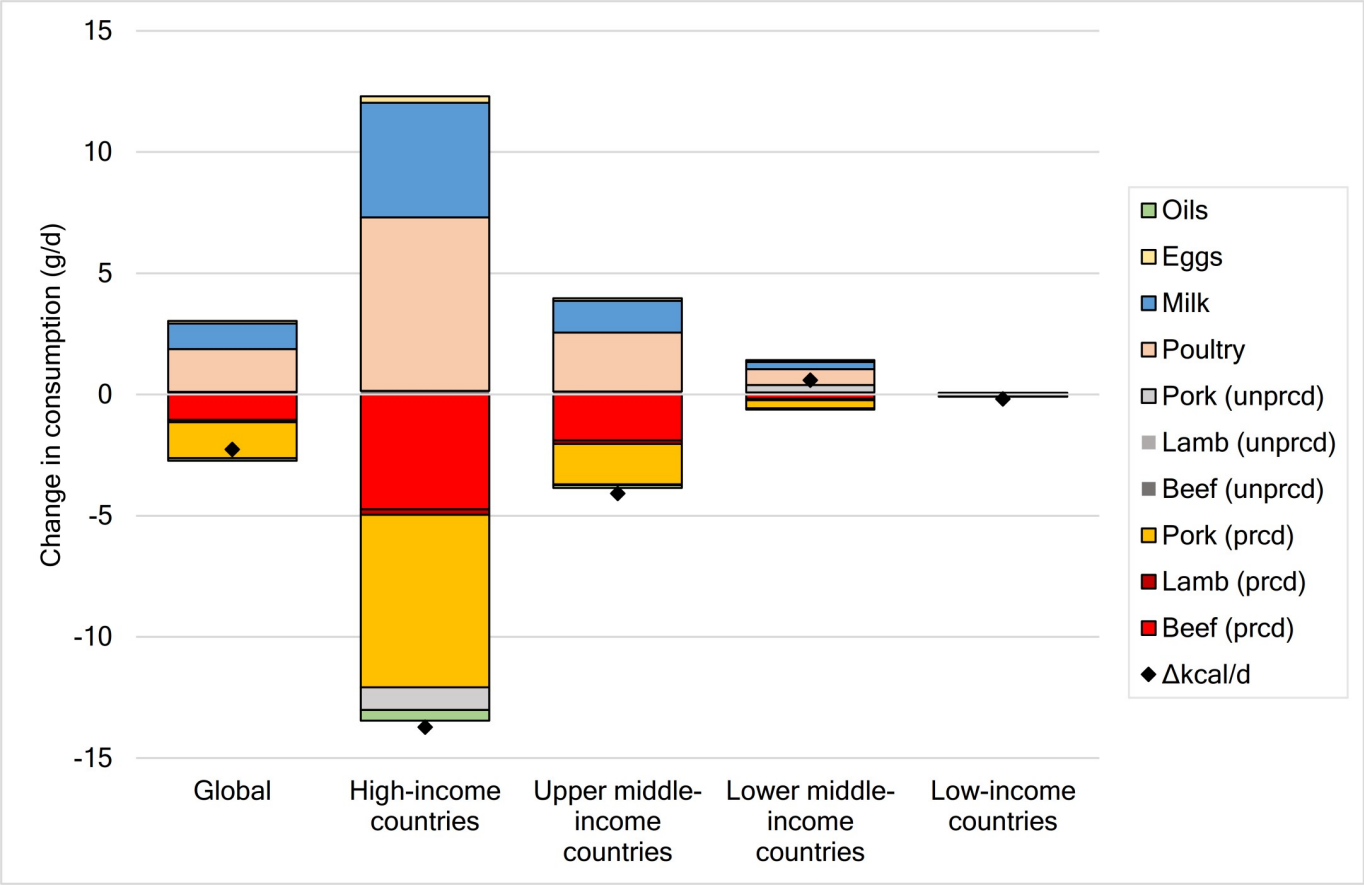
Tax-related changes in food consumption by food commodity and region. Food commodities include processed (prcd) and unprocessed (unprcd) red meats. Changes in food consumption are shown in g/d, with the exception of Δkcal/d which denotes changes in overall energy intake in terms of kcal/d.

Associated with the change in prices were changes in consumption. The greater changes in the price of processed meat compared to red meat resulted in greater changes in consumption for processed meat and also lead to substitution effects, including a shift to poultry and unprocessed red meat (despite a higher price of unprocessed red meat in absolute), and smaller changes in the consumption of milk and eggs, and a small decrease in vegetable oils which is often consumed alongside meat products (Fig 3). The consumption of processed meat decreased by 16% (SI, 9–17; 3 g/d) on average, ranging from 1% (0.1 g/d) in low-income countries to 25% (12 g/d) in high-income countries (Table 1), and up to 37% (28 g/d) for individual countries (Table A11 in S1 File). The consumption of red meat remained similar to a situation without taxation as a result of substitution effects, ranging from a reduction of 0.8% to an increase of 0.7%. Other changes in consumption were a 5% (2 g/d) increase in poultry consumption (0.2–9% across income groups), and smaller increases of 0.4% for milk and eggs (0–0.9% across income groups), and a small decrease of 0.4% for vegetable oils (0–0.9% across income groups) (Fig 3; Table A12 in S1 File).

As a result of the tax-related changes in consumption, the number of deaths attributable to red and processed meat consumption decreased by 222,000 (SI, 38,000–357,000; 9%), from 2,400,000 (SI, 650,000–3,880,000) to 2,118,000 (SI, 609,000–3,379,000). The reductions in the number of attributable deaths were composed of 235,000 (SI, 40,000–380,000) less deaths attributable to processed meat consumption, and 3,200 (SI, -2,400–1,200) more deaths attributable to red meat consumption (Table 1) (note that the combined effect of changes in red and processed meat consumption is generally lower than the sum of the individual effects, because individuals can be affected by both risks simultaneously without two types of the same disease). The changes in attributable deaths corresponded to a reduction in the burden attributable to red and processed meat consumption of 9% on average, ranging from 1% in low-income countries to 17% in high-income countries, and up to 26% for individual countries (Fig 2C; Table A13 in S1 File; https://doi.org/10.5287/bodleian:j0n1Jd5rb).

Following the reduction in health burden, the healthcare-related costs associated with red and processed meat consumption were reduced by USD 41 billion (SI, 10–57), from USD 285 billion (SI, 93–431) to USD 244 billion (SI, 83–374), which represented a cost reduction of 14% on average, ranging from 1% in low-income countries to 17% in high-income countries (Table 1), and up to 26% for individual countries (Table A14 in S1 File; https://doi.org/10.5287/bodleian:j0n1Jd5rb). In comparison, tax revenues amounted to USD 172 billion (SI, 72–215), two thirds (64%) of which came from high-income countries, a sixth to a fifth (16–20%) from middle-income countries, and less than 1% from low-income countries (Table 1; Table A15 in S1 File). Thus, healthcare-related costs under taxation exceeded tax revenues by 42% on average, ranging from 22% in lower middle-income countries to 50% in high-income countries.

### Additional analyses

In a sensitivity analysis, we analysed a cost-compensating taxing scheme in which we increased the prices of red and processed meat until the tax revenues were equal to (i.e. could pay for) the healthcare-related costs associated with their consumption whilst taking into account the feedbacks on consumption and health (section A5 in S1 File). Under cost-compensating taxation, the price increases for red and processed meat approximately doubled compared to marginal-cost pricing, and the reductions in consumption, attributable deaths, and healthcare-related costs of red and processed meat increased by about a third (Table A16 in S1 File).

In addition to changes in diet-related risk factors, consumption changes can influence weight levels and weight-related risks associated with underweight, overweight, and obesity [23,38]. In a sensitivity analysis, we analysed those changes and found that the health impacts from tax-related changes in weight levels associated with changes in calorie intake were small and mostly positive as modest reductions in calorie intake reduced the number of overweight and obese people which in most regions exceed the number of underweight people. The weight impacts led to an additional 3,800 (SI, 3,600–4,100) avoided deaths globally, ranging from 9 additional deaths in low-income countries (which compare to 860 avoided deaths due to reduced red and processed meat consumption) to 2,900 avoided deaths in high-income countries (Tables A17-A18 in S1 File).

Livestock-related emissions are responsible for the majority of food-related greenhouse-gas (GHG) emissions, and for about 14.5% of GHG emissions overall, a similar proportion as from transport [39,40]. Consumption changes towards lower red and processed meat consumption could therefore have major implications for climate change. In a sensitivity analysis, we analysed the potential changes in food-related emissions using emissions intensities of foods obtained from meta-analyses of life-cycle analyses (section A6 in S1 File). We note that the emissions intensities do not account for changes in production methods and technologies that might be associated with changes in consumption. In this static framework, we found that optimal taxation could reduce food-related GHG emissions by 109 MtCO2-eq (CI, 50–139), most of which due to reduced beef consumption (Table A18 in S1 File). The change in emissions represents a reduction of 1.2% globally, ranging from less than one percent (0.6 MtCO2-eq) in low-income countries to 3% (62 MtCO2-eq) in high-income countries, and up to 7% in individual countries (Tables A19-A20 in S1 File).

Red and processed meat consumption is expected to increase in the future, in particular in low and middle-income countries [41,42]. Increases in red and processed meat consumption have implications for optimal tax levels when associated with changes in disease-specific mortality rates and healthcare-related costs. In a final sensitivity analysis, we projected optimal taxes on red and processed meat for the year 2050. As a consequence of socio-economic changes and changes in healthcare-related costs, we found that optimal tax rates more than doubled, ranging from two-fold increases in high-income countries to five-fold increases in low-income countries (Table A21 in S1 File).

## Discussion

The consumption of red and processed meat has been associated with increased mortality from chronic diseases, and red and processed meat have been declared by the World Health Organization to be carcinogenic (processed meat) and probably carcinogenic (red meat) to humans [3–6,17,19,24,25]. One possible policy response to these impacts is market-based regulation in the form of taxes. Here we estimated optimal tax levels for red and processed meat that are based on the (marginal) health cost associated with red and processed meat consumption. By design, the level of health-motivated taxes is context-specific and accounts for disease-specific health costs and mortality in a given location. Consequently, we find that health-motivated taxation of red and processed meat would be low in low-income countries which currently experience a low health and economic burden from red and processed meat consumption, and taxation would be high in high and middle-income countries which currently experience a greater health and economic burden. As income is projected to increase in future years, in particular in low and middle-income countries, it can be expected that optimal tax levels would increase in line with dietary and socio-economic changes.

In our analysis, we estimated a health burden associated with red and processed meat consumption of 2.4 (SI, 0.7–3.9) million attributable deaths in 2020, which represented 4.4% of all projected deaths in the analysis in that year. For the year 2010, the estimates of the number of deaths attributable to red and processed meat consumption are 2.0 (SI, 0.5–3.2) million (Table A22 in S1 File). Our estimates are more comprehensive than the Global Burden of Disease estimate of 0.9 (95% confidence interval (CI), 0.2–1.5) million deaths attributable to red and processed meat in 2010 [22], and 0.7 (CI, 0.6–1.0) million deaths in 2013 [23]. Compared to the GBD estimates, we considered a greater number of disease associations of red and processed meat consumption (CHD, stroke, colorectal cancer, and T2DM compared to CHD, colorectal cancer and T2DM), and we considered minimal exposure levels of zero instead of 11·4–17·1 g/d for red meat and 0–14·3 g/d for processed meat assumed for the GBD estimate for 2013 [22,23]. Both choices are supported by epidemiological evidence (see section A1 in S1 File for a more comprehensive discussion) [43,7,25,24]. Another difference is that we used consumption data that is not standardised to an energy intake of 2000 kcal/d, something that accounts for over and underconsumption. Our analysis might therefore reflect more accurately absolute consumption levels than one based on the energy-standardised data of food composition used by the GBD. Harmonising risk factors, minimum exposures, and energy intake reduced the difference between the GBD estimate and ours from 170% to 78% (risk factors), 72% (risk factors and minimum exposure), 47% (risk factors and energy intake), and 41% (risk factors, minimum exposure, and energy intake), respectively, with overlapping confidence intervals (Table A23 in S1 File).

We estimated an economic burden associated with red and processed meat consumption of USD 285 billion (SI, 93–431) in 2020, which represented 0.3% of the total health expenditure estimated for that year. Our estimate included both direct costs (healthcare expenditure, health service utilization, expenditure on medication) and indirect costs (opportunity costs of informal care, productivity costs due to mortality and morbidity) to provide an estimate of the full health costs of red and processed meat consumption to society. On average, indirect costs represented half to two thirds of the total cost of illness for CHD, stroke, and cancer, but no estimates of indirect costs were available for T2DM. Our estimate of the economic burden attributable to red and processed meat consumption can therefore be considered an underestimate of all costs. Focusing only on the direct cost component would roughly half our estimate (Table A24 in S1 File), and using a more general valuation approach based on a measure for the willingness to pay for a reduction in mortality risk, the so-called value-of-statistical-life approach, would increase our estimate by about a factor of ten [1]. Using disease associations for total cancer (instead of colorectal cancer only) and cardiovascular disease (instead of CHD and stroke only) would roughly double the health and economic burden (Table A23 in S1 File).

Our analysis highlights significant differences between the tax-related impacts on the prices and consumption of red and processed meat. For example, in order to account for the health costs attributable to red and processed meat by adjusting prices, red meat prices would have to increase by more than 20% in high-income countries, and processed meat prices would have to more than double for those countries. Price changes in upper middle-income countries would amount to 7% and 47% for red meat and processed meat, respectively. As a result, processed meat consumption would decrease by about one serving per week (12 g/d) in high-income countries and less than a third of a serving per week (4 g/d) in upper middle-income countries. As consumers are projected to partially switch from processed meat to unprocessed meat and other substitutes such as poultry, red meat consumption would remain largely unchanged in those regions despite its increase price. The total reduction in red and processed meat consumption is therefore lower than one would expect based on the associated changes in prices. Although the changes in red and processed meat consumption are still substantial on a population level, absolute levels of red and processed meat consumption would remain higher in each region (130 g/d in high-income countries and 88 g/d in upper middle-income countries) than recommended by bodies such as the World Cancer Research Institute, which advises consumption of less than 300 g of (uncooked) red meat per week (about 40 g/d), little if any in processed form [26]. Market-based approaches, such as health-motivated taxation, can therefore best be considered as one of a range of measures that would be needed to move diets towards more healthy and sustainable consumption patterns [44].

With respect to the environmental co-benefits of health motivated taxation of red and processed meat, we estimated an emissions reduction potential of about 110 MtCO2-eq globally in 2020, in absence of changes in production methods and technologies that might be associated with changes in consumption. The change in emissions represented a reduction in food-related GHG emissions of 1.2%. The reduction potential is similar to that of technical greenhouse-gas mitigation options, such as rice, livestock, and manure management, which have been estimated to be below 250 MtCO2-eq each [45,46]. Thus, health-motivated taxation of red and processed meat, alongside other measures, could make meaningful contributions to food-related emissions-reduction targets [47]. In another study, we estimated the mitigation potential of environmentally motivated taxation of foods in general to be up to 1 GtCO2-eq in 2020 [48]. However, in environmentally motivated taxation schemes, we found that care has to be taken to compensate for potential reductions in food security, e.g. by using tax revenues for health promotion measures, whereas in the health-motivated approach analysed here, health concerns are built into by design, but all red meat (beef, lamb, pork) is treated equally despite differing emissions intensities. How to optimally combine health and environmentally motivated schemes remains an important question for future research.

Several caveats apply. We assumed that the risk associations between red and processed meat and diet-related diseases are causal based on mechanistic evidence from analyses of the digestive tract for colorectal cancer [3], there are several pathways that plausibly explain the increase in risk for other disease [6,17–19], and the disease associations show a dose-response relationship in cohort studies [17–19]. Whilst the cohort studies controlled for major confounding factors, such as body mass and smoking, we cannot rule out a residual effect of other confounding risk factors. We did not track changes in the nutritional quality of diets, such as levels of micronutrients that could be of concern especially in low-income countries. However, our analysis suggests that cost-compensating tax levels would be zero or close to zero in such environments, and the magnitude of estimated changes is unlikely to have any detrimental impacts in high and middle-income environments where most micronutrient levels are adequate and can be easily obtained from other sources [49]. Due to our focus on consumption, we did not analyse the implications for agricultural production, livelihoods, market adjustments between countries and across time, or how health systems might change under different funding schemes. We hope our comparative regional analysis provides a good starting point for such research.

In our analysis of consumption changes, we used a set of regionally comparable own and cross-price elasticities that describe the substitution between different animal-based foods, and between animal-based foods and some complementary foods, such as vegetable oils. Such substitution is in line with recent reviews of country-level data [50]. However, we cannot rule out substitution effects not captured by the data, such as replacement of processed meat with fish, legumes or grains, especially when changes in caloric intake would be substantial. Both our health estimates and our emissions estimates would change depending on the food groups that would compensate for the reductions in processed meat consumption. For example, greater consumption of sugar and refined carbohydrates, something that is associated with negative health impacts [51], could compensate some of the health benefits associated with lower consumption of processed meat. Similarly, a switch from beef to fish caught by trawling could offset a portion of the emissions reductions associated with reduced processed meat consumption [52]. On the other hand, replacement of red and processed meat with legumes, fruits and vegetables, or whole grains could lead to additional health benefits without significantly affecting the emissions reductions identified here [23,51,52].

## Supporting information

S1 File Supplementary information. (PDF)
