# Supplementary material for: Health-motivated taxes on red and processed meat: A modelling study on optimal tax levels and associated health impacts
Source: PLoS One. 2018 Nov 6;13(11):e0204139. doi: 10.1371/journal.pone.0204139 (PMC6219766; doi:10.1371/journal.pone.0204139)
Supplement: S1 File — (PDF) [file pone.0204139.s001.pdf]

Health-motivated taxes on red and processed meat: a modelling study on optimal tax levels and associated health impacts

Appendix

Contents

Appendix A1. Supplementary health methods ... 3

Table A1 Relative risk parameters associated with one additional serving of 100 g/d. ... 3

Appendix A2. Supplementary costing methods ... 7

Table A3 Healthcare-related costs by region, disease, and component. ... 9

Appendix A3. Supplementary description of IMPACT ... 10

Table A4. Regional aggregation ... 18

Appendix A4. Supplementary methods on linking agricultural and health analyses ... 21

Table A5 Waste percentages at consumption according to FAO ... 21

Appendix A5. Supplementary methods on cost-compensating taxation ... 22

Appendix A6. Supplementary environmental methods ... 23

Table A6 GHG emissions intensities for animal-based foods by food commodity and region (kgCO2-eq per kg) ... 23

Table A7 GHG emissions intensities for plant-based foods by food commodity (kgCO2-eq per kg) ... 23

Appendix A7. Supplementary results ... 24

Table A8. Deaths attributable to red and processed meat consumption (in thousands) associated with one additional serving of red and processed meat by region and disease. ... 24

Table A9. Healthcare-related costs attributable to red and processed meat consumption (in USD billion) associated with one additional serving of red and processed meat by region and disease. ... 25

Table A10. Prices for red and processed meat consumption in the reference (REF) and tax (TAX) scenarios in the year 2020 by region and country. ... 26

Table A11. Consumption of red and processed meat consumption in the reference (REF) and tax (TAX) scenarios in the year 2020 by region and country ... 29

Table A12. Consumption of red and processed meat substitutes in the tax (TAX) scenario in the year 2020 by region and country ... 33

Table A13. Deaths attributable to red and processed meat consumption in the reference (REF) and tax (TAX) scenarios in the year 2020 by region and country ... 36

Table A14. Health costs attributable to red and processed meat consumption in the reference (REF) and tax (TAX) scenarios in the year 2020 by region and country ... 40

Table A15. Tax revenues from red and processed meat (in USD billion) in the year 2020 by region and country. ... 43

Table A17. Changes in energy intake (kcal/d) by food group and region. ... 48

Table A18. Number of avoided deaths (in thousands) associated with tax-related changes in weight levels ... 51

Table A20. Food-related greenhouse gas emissions in the reference (REF) and tax (TAX) scenarios in the year 2020 by region and country. ... 55

Table A20. Food-related greenhouse gas emissions in the reference (REF) and tax (TAX) scenarios in the year 2020 by food group........ 57

Table A21. Main results by region for the year 2050........ 58

Table A22. Main results by region for the year 2010........ 59

Table A24. Main results for a sensitivity analysis which includes only direct health care-related instead of total costs in the calculation of optimal tax levels........ 62

Table A25. Main results for a sensitivity analysis in which own-price elasticities are 10% lower........ 63

Table A26. Main results for a sensitivity analysis in which own-price elasticities are 10% higher........ 64

Table A27. Main results for a sensitivity analysis in which the price of processed meat is equal to the price of red meat........ 65

Table A28. Main results for a sensitivity analysis in which the price of processed meat is 30% less than the price of red meat........ 66

Appendix A1. Supplementary health methods

We estimated the mortality and disease burden attributable to dietary and weight-related risk factors by calculating population attributable fractions (PAFs). PAFs represent the proportions of disease cases that would be avoided when the risk exposure was changed from a baseline situation (the reference scenario with current consumption levels) to a counterfactual situation (scenarios with lower consumption of red and processed meat) using the general formula:[1,2]

$$PAF = \frac{\int RR(x)P(x)dx - \int RR(x)P'(x)dx}{\int RR(x)P(x)dx} \quad (1)$$

where $RR(x)$ is the relative risk of disease for risk factor level x, $P(x)$ is the number of people in the population with risk factor level x in the baseline scenario, and $P'(x)$ is the number of people in the population with risk factor level x in the counterfactual scenario. We assumed that changes in relative risks follow a dose-response relationship,[2,3] and that PAFs combine multiplicatively, i.e. $PAF_{TOT} = 1 - \prod_i (1 - PAF_i)$ where the i's denote independent risk factors.[1,2]

Changes in mortality were calculated by multiplying region and age-specific PAFs by region, age, and disease-specific mortality rates and population numbers.

We used publically available data sources to parameterize the comparative risk analysis. Cause-specific mortality rates and population numbers were adopted from data reported by the Global Burden of Disease project and projected forward using data from the United Nations Population Division. The relative risk parameters were adopted from meta-analysis of prospective cohort studies (Table A1).[4,5] Given that dietary risk factors are predominantly associated with chronic, non-communicable disease mortality, we focused on the health implications of changes in those risk factors for adults (aged 20 and older).

Below we detail our selection of relative risk parameters, and the method used in a sensitivity analyse for estimating the health impacts of weight changes that could occur in response to tax-related changes in the consumption of red meat and the associated substitutions.

Table A1 Relative risk parameters associated with one additional serving of 100 g/d.

Disease	Red meat			Processed meat			Source
	mean	low	high	mean	low	high	
Coronary heart disease	1.00	1.00	1.00	2.02	1.14	3.57	Micha et al (2012)
Stroke	1.13	1.03	1.23	1.23	1.04	1.44	Chen et al (2013)
Colorectal cancers	1.17	1.05	1.31	1.39	1.21	1.64	Chan et al (2011)
Type-2 diabetes mellitus	1.19	1.04	1.37	2.28	1.56	3.35	Feskens et al (2013)

Relative risk parameters

The relative risks of coronary heart diseases due to red and processed meat consumption were adopted from Micha and colleagues[6,7]. Their comprehensive systematic review and meta-analysis of the relationship between meat consumption (processed, red, and total meat) and cardiovascular diseases (coronary heart disease (CHD), type-2 diabetes mellitus (T2DM), and stroke) included 20 studies (17 prospective cohorts and 3 case-control studies) with 1,218,380 individuals from 10 countries. However, analyses of specific subcategories, e.g. total meat consumption and stroke, included significantly less studies. The results show positive associations between consumption of processed and total meat and the incidence of CHD, diabetes mellitus, and stroke. Since the publication of Micha et al[6], updated reviews of the association between meat consumption and stroke have become available. We therefore only adopted the estimates for the association between red and processed meat

consumption and coronary heart disease from the Micha et al study, which indicated no significant association between unprocessed red meat consumption and CHD risk, and a 42% higher risk (RR=1.42, 95% CI, 1.07-1.89) for each 50 g per day increase in processed meat consumption. The latter estimate was based on six observational studies, and remained robust when restricted to prospective cohorts.

The relative risk of stroke due to red and processed meat consumption was adopted from Chen et al[8] which, for stroke, provided an updated meta-analysis of Micha et al[6] containing five large independent cohort studies (compared to two in Micha et al[6]). Chen et al[8] found that consumption of red and/or processed meat increases the risk of stroke, in particular, ischemic stroke. Their dose–response analysis of the primary studies showed that the risk of stroke increased significantly by 13% for each 100 g per day increment in red meat consumption (RR=1.13; 95% CI, 1.03–1.23) and by 11% for each 50 g per day increment in processed meat consumption (RR=1.11; 95% CI, 1.02–1.20), with low study heterogeneity.

The relative risk of T2DM due to red and processed meat consumption was adopted Feskens et al[5] who updates the meta-analysis of Micha et al[6] for T2DM. For red meat, the overall relative-risk estimate, based on 14 individual studies, was 1.13 per 100 g/d (95% CI, 1.03–1.23), and for processed meat, the summary estimate of 21 separate cohorts was 1.32 per 50 g/d (95% CI, 1.19–1.48). As discussed by the authors, there are several plausible explanations for the elevated risks of diabetes and its vascular complications in meat consumers, which support the observational evidence.[5,6,9] Those include dietary fatty acids and cholesterol, haem iron, sodium, nitrates and nitrites, and advanced glycation end products (AGEs).[5,10] The higher content of sodium and nitrite and, probably, AGEs in processed meats could specifically explain the higher risk of T2DM and CHD associated with the consumption of processed meats.

The association between red meat consumption and cancer was comprehensively reviewed in the Second Expert Report "Food, Nutrition, Physical Activity, and the Prevention of Cancer: a Global Perspective" published in 2007 by the World Cancer Research Fund (WCRF) together with the American Institute for Cancer Research,[11] and more recently by the cancer agency of the World Health Organization, the International Agency for Research on Cancer (IARC).[12] The WCRF report was based on reviews and meta-analysis of over 7,000 scientific studies published on cancer prevention, and involved a panel of 21 leading scientists and 9 research centres around the world. With respect to red meat, the report concluded that[11]: red and processed meats are convincing causes of colorectal cancer; there is substantial amount of evidence, with a dose-response relationship apparent from case-control studies (red meat) and cohort studies (processed meat); there is evidence (red meat) and strong evidence (processed meat) for plausible mechanisms operating in humans. The report also noted that there is limited evidence suggesting that red meat is a cause of cancers of the oesophagus, lung, pancreas and endometrium; and that processed meat is a cause of cancers of the oesophagus, lung, stomach and prostate.

The working group contributing to the 2017 IARC came to similar conclusions and classified the consumption of processed meat as "carcinogenic to humans" and the consumption of red meat as "probably carcinogenic to humans".[10] The classification was based on substantial epidemiological data showing a positive association between the consumption of red and processed meat and colorectal cancer, including strong mechanistic evidence. Additionally, a positive association with the consumption of processed meat was found for stomach cancer, and a positive association with the consumption of red meat was found for pancreatic and prostate cancer.

Following the IARC and WCRF reports, we adopted the relative risk of colorectal cancer due to red and processed meat consumption from a meta-analysis by Chan and colleagues.[13] In their dose-response meta-analyses, red meat was statistically significantly associated with increased risk of colorectal cancer (RR for 100 g/day increase = 1.17, 95% CI = 1.05-1.31) based on 8 studies and 4314 cases. The summary relative-risk estimate for every 50 g/d increase in processed meat was 1.18 (95% CI = 1.10-1.28) based on 9 studies and 10863 cases.

In each case, we used zero as minimum risk level. This assumption is based on individual cohort studies that reported details on differentiated consumption levels, and found increasing CVD and cancer risks for very low intakes of red and processed meat.[14,15]

In sensitivity analyses (Table A23), we adopted the results of recent meta-analyses that suggest that red and processed meat consumption could have more general disease associations than those used in our main analysis, in particular on cardiovascular diseases (instead of solely on CHD and stroke) and cancer (instead of solely on colorectal cancer).[4,10] In the sensitivity analysis, we adopted the relative risk of cardiovascular disease (CVD) due to red and processed meat consumption from a 2016 meta-analysis of prospective cohort studies by Wang et al.[4] In their meta-analysis, each (50 g) serving per day of processed meat consumption was associated with a 15% (RR = 1.15; 95% CI, 1.07-1.24) higher risk of cardiovascular mortality based on six studies involving a total of 1,195,947 subjects and 35,426 events, and an 8% (RR = 1.08; 95% CI, 1.06-1.11) higher risk of cancer mortality based on five studies with a total of 1,144,264 subjects and 45,738 cancer deaths. A dose–response analysis based on three studies showed that each (100 g) serving per day of unprocessed red meat consumption was significantly and positively associated with risk of cardiovascular mortality (RR = 1.19; 95% CI, 1.13-1.26) and cancer mortality (RR = 1.12; 95% CI, 1.07-1.17). A previous meta-analysis found similar relationships for unprocessed red meat and CVD, and for processed red meat and CVD, but their category of total meat also included white meats, such as poultry.[10]

Weight estimation

In a sensitivity analysis, we estimated the impacts that tax-related changes in food consumption could have on weight distributions and weight-related mortality by using derived relationships between body mass index and food availability. For the weight-related risk assessment, we estimated changes in weight as shifts in the baseline weight distribution by using the historical relationship between national food availability and mean BMI. We estimated the baseline distribution by fitting a log-normal distribution to WHO estimates of mean BMI and the prevalence of overweight and obesity using a cross-entropy method.[16] Cross-entropy estimation is a Bayesian technique for recovering parameters and data which have been observed imperfectly. The cross-entropy approach redefines the estimation problem as estimating and minimizing the divergence from the original prior while satisfying various constraints. In our application, we take mean BMI values as given and use the cross-entropy method to find the shape and position parameters of the log-normal distribution which jointly minimize the deviation of the estimates of the prevalence of overweight and the prevalence of obesity from the input parameters.

We estimated the relationship between national food availability and mean BMI by pairing FAO food availability data for the years 1980-2009 with WHO data on mean BMI for the same period. We smoothed the time trends of food availability by applying a moving average over three years to reduce year-to-year variability and correct for outliers. Using a polynomial trend yielded the following relationship (R^2 = 0.50):

$$BMI(r) = (-1.28 \cdot 10^{-6}) \cdot kcal(r)^2 + (9.82 \cdot 10^{-3}) \cdot kcal(r) + 7.42 \tag{25}$$

where $kcal(r)$ denotes food availability in region r in terms of kcal per person per day, and $BMI(r)$ denotes the average mean BMI in that region.

Based on the relationship between mean BMI and food availability, we estimated the changes in the weight distribution as follows. We calculated the mean BMI values for the years 2010, 2020, and 2050 using food availability projections from the tax scenarios, and we then used the percentage change in mean BMI between 2010 and 2020/2050 to shift the baseline BMI distribution. In shifting the weight distribution, we held constant the distribution's shape parameter, $\sigma(r)$, and re-calculated its position parameter $\mu(r)$ based on the estimated mean: $\mu(r) = \log BMI(r) - \frac{\sigma(r)^2}{2}$. Analyses were conducted to assess the impact of holding the shape parameter constant, which showed that results were not sensitive to this assumption.

Weight-related risk parameters

Excess weight is an established risk factor for several causes of death, including ischaemic heart disease[17,18], stroke[18–20], and various cancers[11,21–23]. Plausible biological explanations[24–26] and the identification of mediating factors[25,27] suggest that the association between body weight and mortality is not merely statistical association, but a causal link independent of other factors, such as diet and exercise[28–32].

We inferred the parameters describing relative mortality risk due to weight categories from two large, pooled analyses of prospective cohort studies[25,33]. We concentrated on four causes of death: ischaemic/coronary heart disease (CHD), stroke, cancers, and type-2 diabetes mellitus (T2DM). We adopted the relative risks for CHD, stroke, and T2DM from the Prospective Studies Collaboration[25], which analysed the association between BMI and mortality among 900,000 persons in 57 prospective studies that were primarily designed to evaluate risk factors for cardiovascular disease; and we adopted the relative risks for cancer from Berrington de Gonzalez and colleagues[33] who examined the relationship between BMI and mortality in a pooled analysis of 19 prospective studies which included 1.46 million adults and which were predominantly designed to study cancer.

From each study, we adopted the relative risk rates for lifelong non-smokers to minimize confounding and reverse causality, and, to increase comparability, we normalized the relative-risk schedule to the lowest risk which, in each case corresponded to a body-mass index (BMI) of 22.5-25. We then used the number of cause-specific deaths to aggregate the BMI intervals of 2.5 that have been used in the studies to the WHO classification of BMI ranges.

Table A2 Relative risk parameters associated with weight classes.

Disease	Value	underweight	normal	overweight	obese
CHD	mean	0.67	1.00	1.31	1.78
	low	0.65	1.00	1.24	1.64
	high	0.70	1.00	1.39	1.92
Stroke	mean	1.03	1.00	1.07	1.55
	low	0.71	1.00	0.73	1.14
	high	1.47	1.00	1.59	2.11
Cancer	mean	1.11	1.00	1.10	1.40
	low	0.94	1.00	1.04	1.30
	high	1.32	1.00	1.17	1.50
T2DM	mean	1.00	1.00	1.54	7.37
	low	1.00	1.00	1.42	5.16
	high	1.00	1.00	1.68	10.47
Other	mean	1.75	1.00	0.96	1.33
	low	1.50	1.00	0.89	1.22
	high	2.05	1.00	1.03	1.46

Appendix A2. Supplementary costing methods

For estimating healthcare-related costs, we adopted the methodology used in a global assessment of healthcare savings associated with dietary change.[34] In that study, we used a cost-of-illness approach which captures the direct and indirect costs associated with treating a specific disease, including medical and health-care costs (direct), and costs of informal care and from lost working days (indirect).[35] Because global cost-of-illness studies of cardiovascular disease and cancer do not exist at present, we used a cost transfer method, which is similar to the benefit transfer method used in the value of statistical life approach and which has been used in other global assessments of the global economic burden of cardiovascular disease and cancer.[36]

We based our cost-of-illness estimation on a comparative assessment of the economic burden of cardiovascular diseases[35,37] and cancer[38] across the European Union. We adopted the total cost estimate associated with CVD and cancer for the EU in 2009, which included direct costs (healthcare expenditure, health service utilization, expenditure on medication) and indirect costs (opportunity costs of informal care, productivity costs due to mortality and morbidity). We calculated the costs per death due to CHD, stroke, and cancer using EU-wide death-by-disease statistics for the same year.[37] The costs per death by disease (d=CHD, stroke, cancer) in the EU and other regions (r) and years were then estimated by scaling the EU base values for direct and indirect costs by the ratio of health expenditure per capita for direct costs, and by the ratio of GDP per capita (adjusted for purchasing power parity) for indirect costs:

$$CoI_{r,d}^{direct} = CoI_{EU,d}^{direct}\left(\frac{hexp_r}{hexp_{EU}}\right)$$

$$CoI_{r,d}^{indirect} = CoI_{EU,d}^{indirect}\left(\frac{GDP_r}{GDP_{EU}}\right)$$

Productivity losses due to morbidity and mortality, which are a part of the indirect costs, were only included for deaths occurring among those of working age (< 65 years old).

Baseline data on GDP per capita and health expenditure per capita were sourced from the World Bank Development Indicator database, and GDP and population projections for 2020 and 2050 were based on projections by the OECD and IIASA for a "Middle of the Road" socio-economic development pathway (SSP Database, available at: https://tntcat.iiasa.ac.at/SspDb).

Projections for health expenditure per capita in 2020 and 2050 are based on own projections. For those, we linearly regressed past health expenditure per capita on past GDP per capita for the period 1995-2013, and then used the relationships to project future health expenditure per capita based on future GDP trajectories. Most regions exhibited a good fit to the linear regression model (p-values smaller than 0.01 (99% significance level) for n=141 out of 174 regions; 96% with p-values smaller than 0.001). For regions that did not exhibit a good statistical fit (p-values larger than 0.01; n=33 out of 174 regions), we used WHO estimates of health expenditure as percentage of GDP in 2010, and calculated future health expenditure by using future GDP values, holding the percentage of health expenditure to GDP constant. (This is likely to have yielded a conservative estimate as global health expenditure as a percentage of GDP increased by approximately 7% in each of the last three five-year periods.)

We added a transfer error (uncertainty) of 30% to the cost-of-illness estimates based on sub-sample comparison. A comparison of the costs per disease death for individual EU countries in 2009 between estimates based on the cost transfer method and the original estimates indicated a population-weighted deviation of 8-14% in total costs, and of 8-31% in direct costs.

For diabetes, globally comparable health expenditure estimates were available,[39] which we adopted directly and adjusted for potential double-counting of cost components by using incremental cost estimates.[40,41] Diabetes is associated with a high risk of developing complications and co-morbidities, such as CHD, stroke, blindness, renal failure, and amputation, which makes cost coding difficult and diabetes-related healthcare spending

difficult to determine precisely. We adopted estimates of diabetes-related deaths and diabetes-related healthcare expenditure by country from the Diabetes Atlas (6th Edition), and used those to calculate diabetes-related healthcare expenditure per death by region. To avoid double counting of cost components, we adjusted the estimates of diabetes-related health expenditure produced for the Diabetes Atlas by estimates of the incremental cost components that are specifically attributable to diabetes. We adopted an incremental-cost ratio of 50% which is the average of available incremental cost estimates (46-57%).[40,41] Finally, we projected the healthcare expenditure attributable to diabetes death forward to 2020 and 2050 by multiplying the 2013 values by the region-specific increase in healthcare spending per capita between 2013 and 2020 and 2050, respectively. Based on earlier version of the Diabetes Atlas,[39] we assumed an uncertainty interval of 50% around the mean estimates. Table A2 provides an overview of our central estimates.

Table A3 Healthcare-related costs by region, disease, and component. Regions include a global average (World), high-income countries (HIC), upper middle-income countries (UMC), lower middle-income countries (LMC), and low-income countries (LIC).

Region	Disease	Healthcare-related cost by component (USD)				
		total	direct	indirect	indirect, productivity	indirect, care
World	CHD	46,684	14,481	32,409	14,090	18,320
	Stroke	43,023	20,449	22,713	9,603	13,110
	Cancer	53,085	20,302	32,990	22,807	10,183
	T2DM	67,797	67,797	N/A	N/A	N/A
HIC	CHD	163,195	63,309	99,808	43,391	56,417
	Stroke	159,424	89,399	69,947	29,572	40,375
	Cancer	190,445	88,753	101,597	70,238	31,361
	T2DM	366,166	366,166	N/A	N/A	N/A
UMC	CHD	53,117	14,510	40,019	17,398	22,621
	Stroke	47,557	20,489	28,046	11,857	16,189
	Cancer	59,648	20,341	40,737	28,163	12,574
	T2DM	45,752	45,752	N/A	N/A	N/A
LMC	CHD	23,346	4,448	18,919	8,225	10,694
	Stroke	19,527	6,281	13,259	5,605	7,653
	Cancer	25,474	6,236	19,258	13,314	5,944
	T2DM	8,687	8,687	N/A	N/A	N/A
LIC	CHD	6,706	1,435	5,315	2,311	3,005
	Stroke	5,721	2,027	3,725	1,575	2,150
	Cancer	7,377	2,012	5,411	3,741	1,670
	T2DM	1,706	1,706	N/A	N/A	N/A

Appendix A3. Supplementary description of IMPACT

The International Model for Policy Analysis of Agricultural Commodities and Trade (IMPACT) uses economic, water, and crop models to simulate global food production, consumption, and trade of 62 agricultural commodities for over 150 world regions.[42] The regional aggregation used in this study is listed in Table A4. For this study, we used the IMPACT model to produce global food scenarios for the year 2020 and 2050, and we relied on its demand system to estimate changes in food demand resulting from levying health-motivated taxes on red and processed meat.

The IMPACT model system is organized around a core global partial equilibrium multi-market model of agricultural production, demand, trade, and prices. The multi-market model simulates the operation of national and global markets for agricultural commodities, solving for equilibrium prices and quantities. The model specifies supply and demand behaviour in all markets. The following sections describe the elements of the model.

Crop Production

Crop production in IMPACT is simulated through area and yield response functions. (In IMPACT, area is treated as harvested area, which is the total area planted and harvested within a year, and may include multi-cropping or multiple harvests and differ from total arable land or reported physical area). The choice of specifying crop production in this way has a long history in IMPACT and facilitates interaction with commodity experts and land-use specialists, who work in natural units (hectares, tons/hectare). Crop production in IMPACT is specified sub-nationally with the area and yield functions at the level of Food Production Units (FPU). This regional disaggregation permits linking with water models and provides the added benefit of smaller geographical units for aggregating climate change results, which can vary significantly from one location to another. Land used for crop production is divided into irrigated and rainfed systems, capturing the significant differences in yields observed across these cultivation systems and linking directly with the water models which treat irrigated and rainfed water supplies separately.

IMPACT includes the implementation of a land market to manage competing demands for agricultural land from different crops, as well as providing new linkage points to land-use models that work with broader land-use changes, such as conversion of forest to grasslands and agricultural land. It also allows us to separate total area supply (irrigated and rainfed) from individual crop area demands, and allows equilibrium conditions to determine the best economic use of the available land. The total supply of land is assumed to be a function of the "scarcity value" or "shadow price index" of land, which can also be considered a summary of changes in crop prices. The shadow price (WF) is indexed to 1 in the first year and changes based on changing demands from all crops for land area.

$$
\begin{aligned}
QFS_{\mathrm{fpu,lnd}} &= QFSInt_{\mathrm{fpu,lnd}} \times QFSInt2_{\mathrm{fpu,lnd}} \\
QFS &= \text{Land supply} \\
QFSInt &= \text{Land supply intercept (base year supply)} \\
QFSInt2 &= \text{Land supply growth multiplier} \\
fpu &= \text{Food production unit} \\
lnd &= \text{Land type (i.e. irrigated, rainfed)}
\end{aligned}
\qquad (1)
$$

The supply of land is considered exogenous within each year, meaning that farmers are not allowed to adjust the total crop area in the middle of the year. The total land supply over time is driven by exogenous trends on the availability of area for agriculture, as well as endogenous responses to changes in area demand, which is handled in between years. The following equation is applied at the end of each year before solving for a new year.

$$QFSInt2_{\text{fpu,lnd,t}+1} = QFSInt2_{\text{fpu,lnd,t}} \times \left(1 + Landgr_{\text{fpu,lnd}}\right) \times \left(\frac{WF_{\text{fpu,lnd,t}}}{\left\langle WF_{\text{fpu,lnd,t}} \right\rangle_{t-3}} \right)^{L\gamma} \tag{2}$$

$Landgr$ = Exogenous land supply growth rate

$\left\langle WF_{\text{fpu,lnd,t}} \right\rangle_{t-3}$ = Average shadow price of past 3 years

$L\gamma$ = Land supply elasticity

Crop area is specified as an area demand function with respect to changes in the marginal revenue product, changes in land cost, and exogenous non-price trends in harvested area. Crop area elasticities simulate the supply response to changes in the marginal revenue of land represented by the following equation as the interaction of the net price of an activity and the productivity of the activity in using an additional hectare of land.

$$MRP_{\text{j,fpu,lnd}} = Yld_{\text{j,fpu,lnd}} \times PNET_{\text{j,cty}} \tag{3}$$

MRP = Marginal revenue product of land

Yld = Crop yield

$PNET$ = Net price for the activity at the country-level mapped to fpu

j = Activity (crop)

cty = Country

The exogenous trend in harvested area captures changes in area resulting from factors other than direct market effects, such as government programs encouraging cropping expansion, or contraction due to soil degradation, or conversion of land from agriculture to nonagricultural uses. The combination of these endogenous and exogenous factors in area demand are described in the following equation.

$$Area_{\text{j,fpu,lnd}} = AreaInt_{\text{j,fpu,lnd}} \times AreaInt2_{\text{j,fpu,lnd}} \times WF_{\text{fpu,lnd}}^{WF\varepsilon} \times \left(\frac{MRP_{\text{j,fpu}}}{MRP0_{\text{j,fpu}}} \right)^{A\varepsilon} \tag{4}$$

$Area$ = Final crop area

$AreaInt$ = Crop area intercept (base year crop area)

$AreaInt2$ = Exogenous crop area growth multiplier

$WF\varepsilon$ = Elasticity of demand with respect to land shadow price

$MRP0$ = Base year marginal revenue product (used to index prices)

$A\varepsilon$ = Elasticity of area demand with respect to marginal revenue product

Assumptions for exogenous trends are determined by a combination of historical changes in land use and expert judgment on potential future regional dynamics. They are represented as compound growth from the base and are applied between years.

$$AreaInt2_{\text{j,fpu,lnd,t+1}} = AreaInt2_{\text{j,fpu,lnd,t}} \times \left(1 + Areagr_{\text{fpu,lnd}}\right) \tag{5}$$

$Areagr$ = Exogenous area demand growth rate

Competing demands from different crops are handled through an equilibrium equation that determines the land allocation and ensures that all crop area demand must sum up to the total land supply for each FPU.

$$QFS_{fpu,lnd} = \sum_j Area_{j,fpu,lnd} \tag{6}$$

Crop yields are a function of commodity prices, prices of inputs, available water, climate, and exogenous trend factors. The IMPACT model includes four ways that changes in yields are achieved. First, the model assumes a scenario of underlying improvements in yields over time that, to varying degrees, continue trends observed over the past 50-60 years in an informed extrapolation following the concepts introduced in Evenson and Rosegrant,[43] and Evenson and colleagues[44]. These long-run trends, or intrinsic productivity growth rates (IPRs), are intended to reflect the expected increases in inputs, improved seeds, and improvements in management practices. These trends differ and are generally higher for developing countries, where there is considerable scope to narrow the gap in yields compared to developed countries. These IPRs are exogenous to the model, and changes in them are specified as part of the definition of different scenarios. We assume that these underlying trends vary by crop and

region, and that they will decline somewhat over the next fifty years as the pace of technological improvements in developed countries slows, and as developing countries “catch up” to yields in developed countries.

Second, the IMPACT model includes a short-run (annual), endogenous, response of yields to changes in both input and output prices. These yield response functions specify the change in yield as a constant elasticity function of the changes in output prices, with elasticity parameters that can vary by crop and region. The underlying assumption is that farmers will respond to changes in prices by varying the use of inputs, including inputs such as fertilizer, chemicals, and labour that will, in turn, change yields.

Third, climate is assumed to affect yields through two mechanisms. The first mechanism is through the effects of changes in temperature and “weather” due to climate change on crop yields for rainfed and irrigated crops, as calculated from the solution of a crop simulation model (DSSAT[45,46]) for different climate change scenarios. These crop simulations vary by crop type. The DSSAT model is run with detailed time, geographic, and crop disaggregation for different climate change scenarios that are “downscaled” to include weather variation over small geographic areas. This analysis gives changes in average yields due to climate change that are then averaged to generate yield shocks by crop and region (FPU) in the IMPACT model. These long-run climate scenarios generate yield shocks that are assumed to follow simple trends over time, and do not consider extreme events such as droughts or floods.

The fourth mechanism by which climate change affects yields is through variation in water availability for agriculture year-by-year under different climate scenarios. This mechanism is modelled through the use of the IMPACT water models. These include: (1) a global hydrology model that determines run off to the river basins included in the IMPACT model; (2) water basin management models for each FPU that optimally allocate available water to competing non-agricultural and agricultural uses, including irrigation; and (4) a water allocation and stress model that allocates available irrigation water to crops and, when the water supply is less than demand by crop, computes the impact of the water shortage on crop yields accounting for differences among crops and varieties. These yields shocks are then passed to the IMPACT model, affecting year-to-year crop yields.

$$Yield_{\text{j,fpu,lnd}} = YieldInt_{\text{j,fpu,lnd}} \times YieldInt2_{\text{j,fpu,lnd}} \times WatShk_{\text{j,fpu,lnd}} \times CliShk_{\text{j,fpu,lnd}} \times \left(\frac{PNET_{\text{j,cty}}}{PNET0_{\text{j,cty}}} \right)^{Y\varepsilon} \times PF^{F\varepsilon} \tag{7}$$

$Yield$ = Final yield
$YieldInt$ = Yield intercept (base year yield)
$YieldInt2$ = Exogenous yield growth multiplier
$WatShk$ = Water stress shock (from water models)
$CliShk$ = Climate change shock (from water and crop models)
$Y\varepsilon$ = Yield supply elasticity with respect to net price
PF = Input prices
$F\varepsilon$ = Yield supply elasticity with respect to input prices

Final crop production for each FPU and crop (j) is estimated as the product of the solution for its respective area and yield equations, with national production ($QS_{j,cty}$) equal to the summation of the production in all of the relevant FPUs in that country.

$$QS_{\text{j,cty}} = \sum_{\text{fpu,lnd}} \left(Area_{\text{j,fpu,lnd}} \times Yield_{\text{j,fpu,lnd}} \right) \tag{8}$$

Livestock Production

Livestock production is modelled at the FPU level and includes animal numbers, with associated feed demands, and meat/dairy production based on “processing” the animals. Similar to the crop sector, this specification allows for easier translation of information from livestock experts who are used to working with herd-size and feeding requirements. In the current version of the model, there is no modelling of herd dynamics—herd size over time is set exogenously.

Feed demand is a function of the livestock’s own price, the prices of intermediate (feed) inputs, and a trend variable reflecting growth in livestock herds (slaughter rates are implicitly assumed to stay more or less constant

over time). The price elasticities in the livestock supply function are derived in a similar fashion to the crop area and yield elasticities.

$$Animals_{\text{j,fpu,livsys}} = AnimalInt_{\text{j,fpu,livsys}} \times AnimalInt2_{\text{j,fpu,livsys}} \times \left(\frac{PNET_{\text{j,cty}}}{PNET0_{\text{j,cty}}}\right)^{AN\varepsilon} \times \prod_{\text{cfeeds}} \left(\frac{PC_{\text{c,cty}}}{PC0_{\text{c,cty}}}\right)^{Feed\varepsilon}$$

$Animals$ = Number of producing animals
$AnimalInt$ = Animal intercept (initial number of animals)
$AnimalInt2$ = Exogenous population growth
PC = Consumer prices
$PC0$ = Intial consumer prices
$Feed\varepsilon$ = Supply elasticity with respect to changes in feed prices
$livsys$ = Livestock production systems
$cfeeds$ = Feed commodities demanded by livestock sector

(9)

Livestock yields are determined through exogenous growth due to improved animals and management practices. Currently, all price responses in the livestock sector are accounted for in the animal number equations.

$$AnimalYield_{\text{j,fpu,livsys}} = AnimalYieldInt_{\text{j,fpu,livsys}} \times AnimalYieldInt2_{\text{j,fpu,livsys}}$$

$AnimalYield$ = Animal yields
$AnimalYieldInt$ = Initial animal yields
$AnimalYieldInt2$ = Exogenous yield growth

(10)

Total national production ($QS_{j,cty}$) is calculated by multiplying the slaughtered number of animals by the yield per head and summing across FPU and livestock system.

$$QS_{\text{j,cty}} = \sum_{\text{fpu,livsys}} \left(Animals_{\text{j,fpu,livsys}} \times AnimalYield_{\text{j,fpu,livsys}}\right) \quad (11)$$

Production of Processed Goods

Modelling of processed goods (i.e. food oils, oil meals, sugar) has been an active area of improvement for IMPACT, and the development of the activity-commodity framework allows for a general handling of all processed goods in IMPACT through Input-Output matrices (IOMATs) and the use of net prices. The IOMATs represent technical coefficients on input requirements and are specified by quantities of inputs per unit of output (i.e. mt of soybeans per mt of soybean oil), and are calculated from the base data. The net price is the price the producer receives net of input costs. The net price will equal the producer price of the activity whenever there are no intermediate inputs. (Crops and livestock currently do not include intermediate inputs in the PNET equation, and instead directly take input price effects through supply elasticities in the crop yield, and animal number equations).

$$PNET_{\text{j,cty}} = PP_{\text{j,cty}} - \sum_{\text{inputs}} \left(IOMAT_{\text{inputs,j,cty}} \times \left(1 - CSEI_{\text{inputs,cty}}\right) \times PC_{\text{inputs,cty}}\right)$$

$PNET$ = Net price
PP = Producer price
PC = Consumer price of inputs
$CSEI$ = Consumer support estimate on intermediate inputs
$IOMAT$ = Input-output matrix
$inputs$ = Set of commodities (c) that are inputs into activity j

(12)

Production of processed goods are then simulated by a supply function that incorporates both endogenous price effects, as well as exogenous technological change. As opposed to crop and livestock production, processed goods are modelled at the country level instead of at the FPU.

$$QS_{\text{j,cty}} = QSInt_{\text{j,cty}} \times QSINT2_{\text{j,cty}} \times \left(\frac{PNET_{\text{j,cty}}}{PNET0_{\text{j,cty}}} \right)^{QS\varepsilon}$$

$$\begin{aligned} QS &= \text{Total production} \\ QSInt &= \text{Initial production} \\ QSInt2 &= \text{Exogenous productivity growth} \\ QS\varepsilon &= \text{Supply elasticity with respect to net price} \end{aligned} \qquad (13)$$

Commodity Supply and Demand

Total supply of commodities requires mapping from output of production activities to supply of commodities. The mapping is given by:

$$QSUP_{\text{c,cty}} = \sum_{\text{j}} JCRatio_{\text{j,c}} \times QS_{\text{j,cty}}$$

$$\begin{aligned} QSUP &= \text{Total commodity supply} \\ JCRatio &= \text{Activity to commodity mapping} \\ c &= \text{Commodity} \\ cty &= \text{Country} \end{aligned} \qquad (14)$$

The parameter *JCRatio* maps from the activity output to commodities. Usually, each activity produces a matched commodity (e.g., wheat growing activity produces the commodity wheat, and nothing else). The specification, however, is general. There can be many activities producing the same commodity (e.g., different wheat growing activities producing the same wheat commodity) or a single activity producing more than one commodity (e.g., oil seed processing yielding both oil and meal). By convention, the units of j agree with the units of the main commodity produced by the activity (e.g., output of the wheat activity yields the commodity wheat, in the same units), so that the *JCRatio* for this mapped commodity always equals one. Other outputs, if any, from an activity in *JCRatio* are measured as a ratios to the output of the main activity (e.g., tons of meal per ton of production of oil in an oilseed processing plant).

Total domestic demand for a commodity is the sum of household food demand, agricultural intermediate demand (feed, and for process goods), and intermediate demand from other sectors (i.e. biofuels, and industrial uses).

$$QD_{\text{c,cty}} = \sum_{\text{h}} \left(QH_{\text{c,h,cty}} \right) + QInterm_{\text{c,cty}} + QL_{\text{c,cty}} + QBF_{\text{c,cty}} + QOTH_{\text{c,cty}}$$

$$\begin{aligned} QD &= \text{Total commodity demand} \\ QH &= \text{Household food demand} \\ QInterm &= \text{Intermediate demand from Ag-processing sector} \\ QL &= \text{Feed demand from livestock sector} \\ QBF &= \text{Intermediate demand for biofuel feedstock} \\ QOth &= \text{All other demand} \\ h &= \text{Household type} \end{aligned} \qquad (15)$$

Food demand is a function of the price of the commodity and the prices of other competing commodities, per capita income, and total population. Per capita income and population increase annually according to country-specific population and income growth rates. Population and GDP trends vary by scenario and are drawn from the Shared Socio-economic Pathway (SSP) database representing socio-economic scenarios from the IPCC's 5th assessment report. The IMPACT demand elasticities were originally based on elasticities estimated by the USDA,[47] and adjusted to represent a synthesis of average, aggregate elasticities for each region, given the income level and distribution of urban and rural population.[42] Own-price elasticities have been calibrated to a region-specific meta-analysis on the impacts of changes in food prices on food consumption.[48] Over time the elasticities are adjusted to accommodate the gradual shift in demand from staples to high value commodities like meat, especially in developing countries. This assumption is based on expected economic growth, increased urbanization, and continued commercialization of the agricultural sector.

$$QH_{\mathrm{c,h,cty}} = QHInt_{\mathrm{c,h,cty}} \times \left(\frac{pcGDP_{\mathrm{h,cty}}}{pcGDP0_{\mathrm{h,cty}}} \right)^{Inc\varepsilon} \times \left(\frac{(1-CSE_{\mathrm{c,cty}}) \times PC_{\mathrm{c,cty}}}{(1-CSE0_{\mathrm{c,cty}}) \times PC0_{\mathrm{c,cty}}} \right)^{HF\varepsilon}$$

$$\times \prod_{cc \neq c} \left(\frac{(1-CSE_{\mathrm{cc,cty}}) \times PC_{\mathrm{cc,cty}}}{(1-CSE0_{\mathrm{cc,cty}}) \times PC0_{\mathrm{cc,cty}}} \right)^{HF\varepsilon} \times \frac{PopH_{\mathrm{h,cty}}}{PopH0_{h,cty}}$$

QH = Household food demand
$QHInt$ = Initial household food demand
$pcGDP$ = Per capita GDP
$pcGDP0$ = Initial per capita GDP
CSE = Consumer support estimate
$CSE0$ = Initial consumer support estimate
$PopH$ = Population disaggregated by household type
$PopH0$ = Initial household population
$Inc\varepsilon$ = Income demand elasticity
$HF\varepsilon$ = Price demand elasticty

$\left(\frac{(1-CSE) \times PC}{(1-CSE0) \times PC0} \right)^{HF\varepsilon}$ = Own-price response

$\prod_{cc \neq c} \left(\frac{(1-CSE) \times PC}{(1-CSE0) \times PC0} \right)^{HF\varepsilon}$ = Cross-price response (16)

Feed demand is a derived intermediate demand. It is determined by two components: (1) animal feed requirements determined by livestock production and livestock feed requirements and (2) price effects that take into account potential substitution possibilities among different feeds. The equation also incorporates a technology parameter that indicates improvements in feeding efficiencies over time.

$$QL_{\mathrm{c,cty}} = \sum_{\mathrm{jlvst}} \left(QS_{\mathrm{jlvst,cty}} \times \mathrm{Re}q_{\mathrm{jlvst,c,cty}} \right) \times \prod_{\mathrm{cfeeds}} \left(\frac{PC_{\mathrm{c,cty}}}{PC0_{\mathrm{c,cty}}} \right)^{LFD\varepsilon}$$

QL = Total feed demand for livestock sector
QS = Total production of each livestock activity (17)
$\mathrm{Re}q$ = Feed requirements for each livestock activity
$LFD\varepsilon$ = Price elasticity of demand for feed
$jlvst$ = Set of livestock producing activities

Intermediate demand is a derived demand that is based on the demand for final processed goods, such as food oils and sugar. The input-output matrix determines the proportions of inputs (c) required for each producing activity (j).

$$QDInterm_{\mathrm{c,cty}} = \sum_{\mathrm{j}} \left(IOMat_{\mathrm{c,j,cty}} \times QS_{\mathrm{j,cty}} \right)$$

$QDInterm$ = Intermediate demand (18)
$IOMat$ = Input-Output matrix

Exogenous biofuel feedstock demand is determined through exogenous growth rates which represent government mandates to encourage the production of biofuels, though adjusted in various scenarios where the mandates are infeasible, or adjusted to reflect scenarios on the role of first or second generation biofuels. The biofuel feedstock demand equation also allows for a price response for biofuels to allow for substitution across different potential feedstocks, as well as to reflect the reality that increasing food prices would put pressure to ease biofuel mandates.

$$QBF_{\text{c,cty}} = QBFInt_{\text{c,cty}} \times QBFINT2_{\text{c,cty}} \times \prod_{\text{c}} \left(\frac{PC_{\text{c,cty}}}{PC0_{\text{c,cty}}} \right)^{BF\varepsilon} \tag{19}$$

QBF = Biofuel feedstock demand
$QBFInt$ = Initial demand from biofuel sector
$QBFInt2$ = Exogenous growth in demand from biofuels
$BF\varepsilon$ = Price elasticity of demand for biofuel feedstock

Other demand summarizes all other demands for agricultural products from sectors outside of the focus of IMPACT (e.g. seeds, industrial use, etc.). It is simulated under two different equations. The primary method follows the household food demand equation, and is sensitive to changes in income, population and prices.

$$QOth_{\text{c,cty}} = QOthInt_{\text{c,cty}} \times \left(\frac{pcGDP_{\text{cty}}}{pcGDP0_{\text{cty}}} \right)^{IOth\varepsilon} \times \left(\frac{POP_{\text{cty}}}{POP0_{\text{cty}}} \right) \times \prod_{cc} \left(\frac{PC_{\text{c,cty}}}{PC0_{\text{c,cty}}} \right)^{POth\varepsilon} \tag{20}$$

$QOth$ = Other Demand
$QOthInt$ = Initial other demand
$IOth\varepsilon$ = Income demand elasticity for other demand
$POth\varepsilon$ = Price demand elasticity for other demand

Markets, Trade, and Equilibrium Prices

The system of equations is written in the General Algebraic Modeling System (GAMS) programming language.[49] The solution of these equations is achieved by the Path solver, which is included in the GAMS system. This procedure finds a set of domestic and world prices for all crops that "clear" domestic and international commodity markets. The world price (PW) of a commodity is the equilibrating mechanism for traded commodities—when an exogenous shock is introduced in the model, PW will adjust to clear world markets and each adjustment is passed back to the effective producer (PS) and consumer (PD) prices via the price transmission equations. Changes in domestic prices subsequently affect commodity supply and demand, necessitating their iterative readjustments until world supply and demand balance and world net trade again equals zero. For non-traded commodities, domestic prices in each country adjust to equate supply and demand within the country.

IMPACT assumes a closed world economy—at the end of every year the world's production must equal the world's demand. This constraint is ensured by the following equation, where the sum of net trade over the globe must equal zero.

$$\sum_{\text{cty}} NT_{\text{c,cty}} = 0 \tag{21}$$

NT = Net Trade

National production and demand for tradable commodities are linked to world markets through trade. Commodity trade by country (cty) is a function of domestic production, domestic demand, and stock change. (Note that stocks are constant and exogenous). Regions with positive net trade are net exporters, while those with negative values are net importers. This specification does not permit a separate identification of international trade by country of origin and destination—all countries export to and import from a single global market.

$$NT_{\text{c,cty}} = QSUP_{\text{c,cty}} - QD_{\text{c,cty}} - QSt_{\text{c,cty}} \tag{22}$$

NT = Net trade
QSt = Change in stocks

Prices are endogenous in the system of equations for food, and are calibrated to year 2005 commodity prices.[10–12] Price data were based on the Agricultural Market Access Database (AMAD) of commodity prices[52], adjusted for the effect of trade policy represented by taxes and tariffs, price policies expressed in terms of producer support estimates (PSE), consumer support estimates (CSE), and the cost of moving products from one market to another represented by marketing margins (MM). Export taxes and import tariffs are drawn from GTAP data (Global Trade Analysis Project at Purdue University) and reflect trade policies at the national level.[53–55] PSEs and CSEs represent public policies to support production and consumption by creating wedges between world and domestic prices. PSEs and CSEs are based on OECD estimates and are adjusted by expert judgment to reflect

regional trade dynamics.[56] Marketing margins (MM) reflects other factors such as transport and marketing costs of getting goods to various markets and are based on expert opinion on the quality and availability of transportation, communication, and market infrastructure. We adopted the data on consumer prices for our consumption-based policy analysis.

In the model, PSEs, CSEs, and MMs are expressed as percentages (ad valorem) of the world price. To calculate producer prices the appropriate wedges are applied to the domestic consumer prices (PC) and represent the mark-up observed in domestic markets from the farm-gate or factory-gate prices producers receive. The producer price of an activity is the weighted sum of the prices of the commodities associated with that activity.

$$PP_{j,cty} \times \left(1 + MMJ_{j,cty}\right) = \left(1 + PSE_{j,cty}\right) \times \sum_{c} JCRatio_{j,c,cty} \times PC_{c,cty}$$

$$\begin{aligned} PP &= \text{Producer price} \\ MMJ &= \text{Farm(factory)-gate to domestic market Marketing Margin (MM)} \\ PSE &= \text{Producer support estimate, ad valorem component} \\ JCRatio &= \text{mapping from activities (j) to commodities (c)} \end{aligned} \qquad (23)$$

How consumer prices are determined in IMPACT depends on the state of tradability of the commodity. Commodities can be specified as either tradable or non-tradable. Traded commodity prices are determined in international markets. Non-traded commodities, are those commodities whose prices are determined in national markets, without direct links to international markets. Examples include sugarcane, sugar beets, and grass, where all demand is intermediate demand from domestic sectors (sugar processing, and livestock). These commodity prices are determined endogenously by country and ensure that domestic supply equals domestic demand.

$$QSUP_{c,cty} = QD_{c,cty} \qquad (24)$$

Non-traded commodity are indirectly linked to world markets through the demand for final products (i.e. sugar), and potential substitution from tradable commodities (i.e. grass and other feeds).

Table A4. Regional aggregation

**High-income countries (HIC)**		
Australia	Hungary	Portugal
Austria	Iceland	Republic of Korea
Belgium and Luxembourg	Ireland	Rest of Arab Peninsula
Canada	Israel	Saudi Arabia
Croatia	Italy	Slovakia
Cyprus	Japan	Slovenia
Czech Republic	Netherlands	Spain
Denmark	New Zealand	Sweden
Finland	Norway	Switzerland
France	Other Caribbean	United Kingdom
Germany	Other Southeast Asia	United States of America
Greece	Poland	
**Upper middle-income countries (UMC)**		
Botswana	Dominican Republic	Baltic States
Algeria	Jamaica	Kazakhstan
Gabon	Mexico	Other Balkans
Namibia	Panama	Romania
South Africa	Peru	Russian Federation
Argentina	Uruguay	Fiji
Brazil	Venezuela (Bolivarian Republic of)	Malaysia
Chile	Lebanon	
Colombia	Libya	
Costa Rica	Bulgaria	
Cuba	Belarus	
**Lower middle-income countries (LMC)**		
Angola	Paraguay	Turkmenistan
Côte d'Ivoire	El Salvador	Ukraine
Cameroon	Djibouti	Bhutan
Lesotho	Egypt	Indonesia
Nigeria	Iran (Islamic Republic of)	India
Papua New Guinea	Jordan	Sri Lanka
Other Indian Ocean	Pakistan	Thailand
Swaziland	Sudan	Timor-Leste
Belize	Syrian Arab Republic	China
Bolivia (Plurinational State of)	Tunisia	Mongolia
Ecuador	Albania	Philippines
Guyanas South America	Armenia	
Guatemala	Azerbaijan	
Honduras	Georgia	
Nicaragua	Republic of Moldova	
**Low-income countries (LIC)**		
Burundi	Mali	Afghanistan
Benin	Mozambique	Yemen
Burkina Faso	Mauritania	Kyrgyzstan
Central African Republic	Malawi	Tajikistan
Congo	Niger	Uzbekistan

Eritrea	Senegal	Bangladesh
Ethiopia	Sierra Leone	Myanmar
Ghana	Chad	Nepal
Guinea	Togo	Cambodia
Gambia	United Republic of Tanzania	Lao People's Democratic Republic
Guinea-Bissau	Uganda	Solomon Islands
Kenya	Zambia	
Liberia	Viet Nam	
Madagascar	Haiti	
**Low and middle-income countries of Africa (AFR_LMIC)**		
Algeria	Ghana	Other Indian Ocean
Angola	Guinea	Senegal
Benin	Guinea-Bissau	Sierra Leone
Botswana	Kenya	South Africa
Burkina Faso	Lesotho	Swaziland
Burundi	Liberia	Togo
Cameroon	Madagascar	Uganda
Central African Republic	Malawi	United Republic of Tanzania
Chad	Mali	Zambia
Congo	Mauritania	
Côte d'Ivoire	Mozambique	
Eritrea	Namibia	
Ethiopia	Niger	
Gabon	Nigeria	
Gambia	Senegal	
**Low and middle-income countries of the Eastern Mediterranean (EMR_LMIC)**		
Lebanon	Tunisia	
Libya	Afghanistan	
Djibouti	Yemen	
Egypt		
Iran (Islamic Republic of)		
Iraq		
Jordan		
Pakistan		
Sudan		
Syrian Arab Republic		
**Low and middle-income countries of Europe (EUR_LMIC)**		
Bulgaria	Georgia	
Belarus	Republic of Moldova	
Baltic States	Turkmenistan	
Kazakhstan	Ukraine	
Other Balkans	Kyrgyzstan	
Romania	Tajikistan	
Russian Federation	Uzbekistan	
Albania		
Armenia		
Azerbaijan		
**Low and middle-income countries of South-East Asia (SEA_LMIC)**		

Bhutan
Indonesia
India
Sri Lanka
Thailand
Timor-Leste
Bangladesh
Myanmar
Nepal
**Low and middle-income countries of the Western Pacific (WPR_LMIC)**
Fiji
Malaysia
Solomon Islands
China
Mongolia
Philippines
Papua New Guinea
Viet Nam
Cambodia
Lao People's Democratic Republic

Appendix A4. Supplementary methods on linking agricultural and health analyses

Conversion from food demand into food consumption

Baseline food production and availability, as estimated by the IMPACT model, are calibrated using food balance sheets supplied by the Food and Agriculture Organization of the United Nations (FAO). The FAO[57–59] states that:

> The quantities of food available for human consumption, as estimated in the food balance sheet, relate to the quantities of food reaching the consumer. Waste on the farm and during distribution and processing is taken into consideration as an element in the food balance sheet. However, The amount of food actually consumed may be lower than the quantity shown in the food balance sheet depending on the degree of losses of edible food and nutrients in the household, e.g. during storage, in preparation and cooking (which affect vitamins and minerals to a greater extent than they do calories, protein and fat), as plate-waste, or quantities fed to domestic animals and pets, or thrown away.

For the dietary risk assessment, we converted the food availability estimates into food consumption estimates by using regional data on food wastage at the consumption level, combined with conversion factors into edible matter[60]. Table A4 lists the waste percentages and conversion factors used. No conversion factor was used for red meat, because the waste percentages reported in Table A4 were obtained for carcass weight (including bone), and therefore included wastage of non-edible parts.

Table A5 Waste percentages at consumption according to FAO[60]

Food items	Europe	USA, Canada, Oceania	Industri-alized Asia	Sub-Saharan Africa	North Africa, West and Central Asia	South and Southeast Asia	Latin America
Cereals	0.25	0.27	0.2	0.01	0.12	0.03	0.1
Roots and tubers	0.17	0.3	0.1	0.02	0.06	0.03	0.04
Oilseeds and pulses	0.04	0.04	0.04	0.01	0.02	0.01	0.02
Fruits and vegetables	0.19	0.28	0.15	0.05	0.12	0.07	0.1
Meat	0.11	0.11	0.08	0.02	0.08	0.04	0.06
Milk	0.07	0.15	0.05	0.001	0.02	0.01	0.04

Conversion factors into edible matter: 0.82 for roots, 0.79 for maize, 0.78 for wheat, 1 for rice, 0.78 for other grains, 0.77 for fruits and vegetables, 1 for meat, 1 for oilseeds and pulses, 1 for milk

Appendix A5. Supplementary methods on cost-compensating taxation

We adopted the emissions factors for livestock from a global life cycle assessment with regional detail undertaken by the Food and Agriculture Organization (FAO) (Table A5)[61]. The assessment included all main emissions sources along the food supply chain from the farm gate to the retail point, including land use, feed
For a sensitivity analysis, we used an optimization algorithm to calculate cost-compensating tax levels for red and processed meat and the associated health impacts (Figure A1). The algorithm consisted of six steps. First, we estimated the health impacts associated with the current and projected consumption levels of red and processed meat. Second, we estimated the health costs associated with those health impacts. Third, we calculated initial tax levels for red and processed meat which would result in tax revenues equal to the health costs if consumption did not change. Fourth, we estimated the impacts of initial tax levels on the consumption of red and processed meat. Fifth, we calculated tax revenues based on the new consumption levels, and compared those to the health costs. Sixth, we adjusted tax levels to be lower if revenues exceeded health costs, and higher if health costs exceeded revenues, and we iterated the calculation of consumption changes, health impacts, health costs, and tax levels until tax revenues equalled health costs after changes in consumption levels.

Figure A1 Schematic of algorithm used to calculate health-motivated and cost-compensating tax levels for red meat.

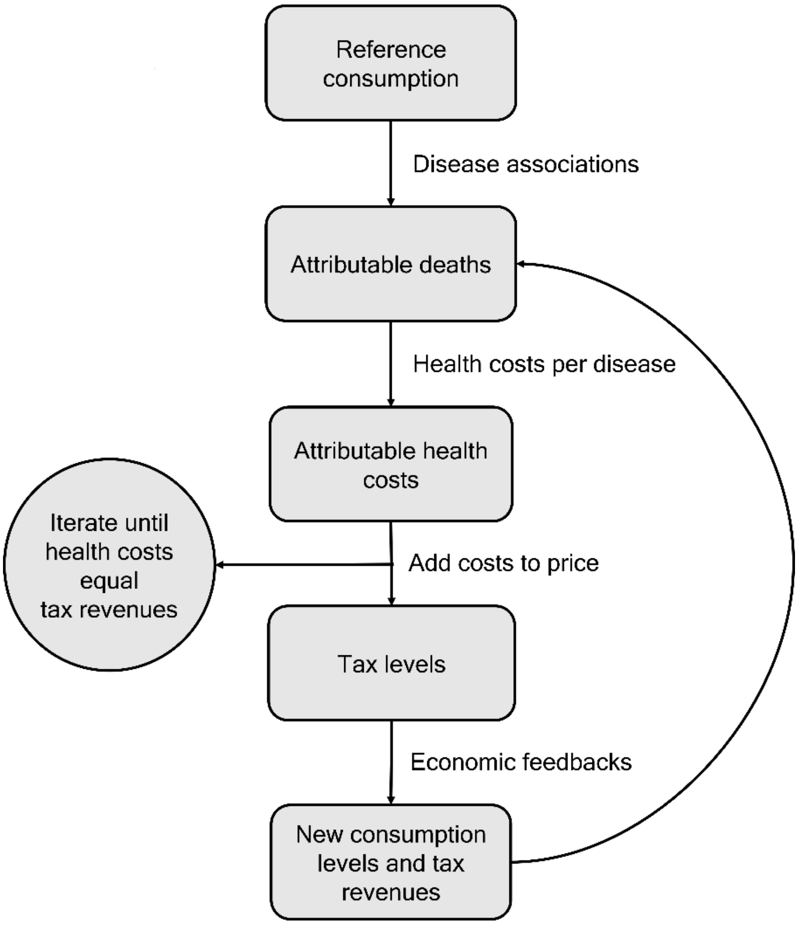

Appendix A6. Supplementary environmental methods

We adopted the emissions factors for livestock from a global life cycle assessment with regional detail undertaken by the Food and Agriculture Organization (FAO) (Table A5)[61]. The assessment included all main emissions sources along the food supply chain from the farm gate to the retail point, including land use, feed production, animal production, processing, and transport, including international trade. Emissions factors for non-animal products were adopted from a comprehensive meta-analysis of life cycle assessments including 555 estimates (Table A6)[62].

Table A6 GHG emissions intensities for animal-based foods by food commodity and region (kgCO2-eq per kg). Based on Gerber and colleagues[61]. Regions include high-income countries (HIC), upper middle-income countries (UMC), lower middle-income countries (LMC), low-income countries (LIC); and the low and middle-income countries of Africa (AFR_LMIC), America (AMR_LMIC), the Eastern Mediterranean (EMR_LMIC), Europe (EUR_LMIC), South-East Asia (SEA_LMIC), the Western Pacific (WPR_LMIC), and a global average (World).

Region	Beef	Lamb	Pork	Poultry	Milk	Eggs
World	53.05	25.58	6.08	5.76	3.93	3.87
HIC	26.83	21.25	5.75	5.33	1.86	3.58
UMC	53.31	25.97	6.64	5.63	3.85	3.70
LMC	57.96	26.03	6.04	5.92	3.98	3.81
LIC	64.58	28.75	6.05	5.72	6.48	4.73
AFR_LMIC	71.03	30.98	6.05	5.40	8.98	5.93
AMR_LMIC	72.00	26.00	7.20	5.85	3.80	3.80
EMR_LMIC	52.50	28.52	6.05	6.03	4.84	3.27
EUR_LMIC	18.91	24.68	5.91	5.17	2.56	2.88
SEA_LMIC	70.07	27.77	6.04	6.16	4.71	3.36
WPR_LMIC	46.89	22.96	6.00	5.80	2.40	4.20

Table A7 GHG emissions intensities for plant-based foods by food commodity (kgCO2-eq per kg). Based on Tilman and Clark[62].

Food item	Emissions intensity (kgCO2-eq per kg)
Vegetable oils	5.17
Rice	1.89
Wheat	0.65
Vegetables	0.64
Other grains	0.55
Maize	0.34
Oil crops	0.32
Sugar	0.26
Fruits (tropical)	0.26
Legumes	0.26
Fruits (temperate)	0.17
Roots	0.09

Appendix A7. Supplementary results

Table A8. Deaths attributable to red and processed meat consumption (in thousands) associated with one additional serving of red and processed meat by region and disease. Regions include all countries (Global), high-income countries (HIC), upper middle-income countries (UMC), lower middle-income countries (LMC), and low-income countries (LIC). Diseases include coronary heart disease (CHD), stroke, colon and rectum cancers, and type-2 diabetes mellitus (T2DM). Values are displayed for the mean, low, and high values of 95% confidence intervals of the relative-risk distribution.

Region	Disease	Red meat			Processed meat		
		mean	low	high	mean	low	high
Global	All diseases	1123.11	309.52	1728.04	5910.39	1959.67	7956.93
	CHD				3543.96	990.9	4588.8
	Stroke	781.57	211.38	1198.26	1353.59	286.22	2145.81
	Colon and rectum cancers	98.62	35.23	147.14	198.57	126.87	263.44
	T2DM	242.93	62.91	382.63	814.26	555.69	958.88
HIC	All diseases	153.72	45.81	228.58	790.2	312.57	932.89
	CHD				485.5	162.06	530.59
	Stroke	81.74	22.55	122.87	136.9	30.37	207.83
	Colon and rectum cancers	39.19	14.23	57.36	75.85	49.77	97.57
	T2DM	32.79	9.02	48.35	91.95	70.37	96.9
UMC	All diseases	167.6	46.16	257.74	986.63	346.84	1260.48
	CHD				631.82	184.4	777.68
	Stroke	104.65	28.17	160.88	176.27	37.92	274.92
	Colon and rectum cancers	16.2	5.73	24.42	32.2	20.56	42.72
	T2DM	46.74	12.27	72.44	146.32	103.96	165.16
LMC	All diseases	680.82	185.93	1048.77	3573.14	1111.49	4981.28
	CHD				2129.62	566.6	2873.45
	Stroke	512.57	139.11	783.79	902.51	189.04	1442.59
	Colon and rectum cancers	37.62	13.37	56.55	79.41	49.61	108.01
	T2DM	130.64	33.45	208.43	461.6	306.24	557.22
LIC	All diseases	103.33	26.99	164.76	456.15	154.17	635.26
	CHD				233.75	61.73	318.01
	Stroke	71.86	18.75	113.65	119.81	25.14	191.21
	Colon and rectum cancers	4.29	1.46	6.74	8.47	5.29	11.51
	T2DM	27.17	6.78	44.36	94.14	62.01	114.53

Table A9. Healthcare-related costs attributable to red and processed meat consumption (in USD billion) associated with one additional serving of red and processed meat by region and disease. Regions include all countries (Global), high-income countries (HIC), upper middle-income countries (UMC), lower middle-income countries (LMC), and low-income countries (LIC). Diseases include coronary heart disease (CHD), stroke, colon and rectum cancers, and type-2 diabetes mellitus (T2DM). Values are displayed for the mean, low, and high values of 95% confidence intervals of the relative-risk distribution.

Region	Disease	Red meat			Processed meat		
		mean	low	high	mean	low	high
Global	All diseases	78.07	22.47	116.9	401.27	152.06	503.78
	CHD				240.65	72.82	291.01
	Stroke	43.34	11.91	65.44	74.93	16.13	116.92
	Colon and rectum cancers	12.14	4.39	17.86	23.72	15.47	30.75
	T2DM	22.59	6.16	33.6	61.97	47.64	65.1
HIC	All diseases	39.69	11.71	58.71	175.61	82.31	197.62
	CHD				92.77	32.07	97.97
	Stroke	14.42	3.98	21.64	23.87	5.35	35.87
	Colon and rectum cancers	8.64	3.14	12.64	16.43	10.87	20.93
	T2DM	16.62	4.58	24.43	42.54	34.01	42.85
UMC	All diseases	13.4	3.71	20.58	81.95	29.06	103.84
	CHD				53.77	15.73	66
	Stroke	8	2.15	12.3	13.44	2.9	20.94
	Colon and rectum cancers	1.47	0.52	2.22	2.91	1.86	3.85
	T2DM	3.93	1.04	6.07	11.82	8.58	13.05
LMC	All diseases	23.19	6.57	34.77	133.19	37.49	187.49
	CHD				87.26	23.24	117.6
	Stroke	19.61	5.43	29.43	35.41	7.42	56.59
	Colon and rectum cancers	1.88	0.68	2.76	4.08	2.55	5.55
	T2DM	1.71	0.46	2.58	6.44	4.28	7.75
LIC	All diseases	0.85	0.23	1.33	4.23	1.21	5.91
	CHD				2.67	0.72	3.56
	Stroke	0.72	0.19	1.12	1.2	0.25	1.9
	Colon and rectum cancers	0.05	0.02	0.08	0.11	0.07	0.15
	T2DM	0.07	0.02	0.12	0.25	0.17	0.3

Table A10. Prices for red and processed meat consumption in the reference (REF) and tax (TAX) scenarios in the year 2020 by region and country (abs: absolute values in USD per kg, chg: changes between scenarios in USD per kg, pct: percentage change between scenarios). Countries are sorted by greatest percentage changes for processed meat in the TAX scenario.

Region	Red meat				Processed meat			
	REF	TAX			REF	TAX		
	abs	abs	chg	pct	abs	abs	chg	pct
Global	6.75	7.03	0.28	4.17	5.74	7.19	1.45	25.21
HIC	4.42	5.36	0.94	21.36	3.75	7.93	4.17	111.17
UMC	6.05	6.44	0.39	6.51	5.14	7.55	2.41	46.85
LMC	6.93	7.08	0.15	2.16	5.89	6.75	0.86	14.62
LIC	8.75	8.77	0.02	0.23	7.44	7.54	0.10	1.34
AFR_LMIC	9.52	9.55	0.03	0.35	8.09	8.25	0.16	2.00
AMR_LMIC	5.67	5.93	0.25	4.45	4.82	5.93	1.11	22.97
EMR_LMIC	10.30	10.36	0.06	0.56	8.76	9.28	0.52	5.97
EUR_LMIC	7.25	7.85	0.60	8.25	6.16	10.98	4.81	78.11
SEA_LMIC	7.70	7.77	0.07	0.87	6.55	6.96	0.41	6.29
WPR_LMIC	4.46	4.73	0.27	5.97	3.79	5.17	1.38	36.38
SWE	3.88	4.93	1.06	27.20	3.30	9.41	6.11	185.41
NOR	4.44	5.95	1.51	34.00	3.77	10.51	6.74	178.61
AUT	3.60	4.70	1.10	30.61	3.06	8.17	5.12	167.42
DEU	3.59	4.59	1.01	28.14	3.05	8.10	5.05	165.84
USA	4.28	5.72	1.45	33.81	3.63	9.57	5.93	163.26
SVK	3.96	4.58	0.63	15.80	3.36	8.81	5.45	162.11
CHP	4.19	5.38	1.20	28.62	3.56	9.24	5.68	159.71
FNP	3.83	4.47	0.64	16.60	3.26	8.02	4.76	145.96
CZE	3.94	4.54	0.60	15.17	3.35	8.15	4.80	143.41
CAN	4.12	5.02	0.89	21.68	3.50	8.04	4.54	129.45
RUS	7.26	8.28	1.02	14.04	6.17	14.16	7.99	129.39
BLT	5.04	5.59	0.54	10.78	4.29	9.40	5.11	119.22
DNK	3.86	4.99	1.13	29.21	3.28	7.19	3.91	119.07
NLD	3.75	4.78	1.03	27.44	3.19	6.87	3.68	115.44
OSA	3.91	4.47	0.56	14.27	3.32	7.08	3.76	113.03
AUS	4.84	5.72	0.88	18.25	4.11	8.60	4.49	109.16
BGR	5.49	6.36	0.87	15.90	4.67	9.67	5.01	107.30
BLX	3.98	4.79	0.81	20.43	3.38	6.96	3.57	105.64
HUN	4.35	4.91	0.56	12.89	3.70	7.54	3.84	103.73
ITP	3.93	4.69	0.76	19.42	3.34	6.71	3.37	100.84
JPN	4.45	5.33	0.88	19.78	3.78	7.40	3.62	95.89
GRC	5.50	6.14	0.64	11.66	4.68	9.12	4.45	95.10
POL	3.85	4.45	0.60	15.55	3.27	6.37	3.09	94.42
HRV	4.93	5.49	0.56	11.43	4.19	7.73	3.54	84.47
PRT	3.82	4.56	0.73	19.19	3.25	5.98	2.73	83.88
IRL	3.97	4.45	0.47	11.89	3.38	6.08	2.71	80.13
UKP	4.39	4.99	0.60	13.64	3.73	6.68	2.95	78.93
BLR	6.80	7.19	0.39	5.77	5.78	10.17	4.40	76.05
SPP	3.86	4.41	0.55	14.17	3.28	5.69	2.41	73.35
CRB	5.30	5.91	0.61	11.58	4.50	7.71	3.21	71.29
FRP	4.01	4.73	0.72	18.07	3.40	5.73	2.32	68.27

NZL	5.05	5.65	0.60	11.91	4.29	7.00	2.71	63.29
ROU	5.33	5.80	0.47	8.88	4.53	7.36	2.83	62.59
SVN	4.35	4.90	0.56	12.83	3.69	6.00	2.31	62.48
KAZ	7.75	8.18	0.43	5.49	6.59	10.47	3.88	58.92
CYP	4.53	4.89	0.36	7.87	3.85	5.96	2.11	54.70
UKR	8.43	8.76	0.33	3.88	7.16	10.81	3.65	50.93
ISR	5.00	5.45	0.45	9.06	4.25	6.37	2.12	49.94
KOR	4.57	5.03	0.46	10.07	3.88	5.79	1.91	49.29
ISL	6.69	7.19	0.50	7.40	5.69	8.36	2.67	46.92
MDA	5.75	6.02	0.27	4.68	4.89	7.06	2.17	44.35
CHM	4.31	4.61	0.30	7.00	3.66	5.22	1.56	42.55
OBN	4.94	5.29	0.35	7.05	4.20	5.94	1.74	41.44
CUB	5.47	5.73	0.26	4.71	4.65	6.55	1.90	40.88
GEO	7.22	7.56	0.34	4.69	6.14	8.41	2.27	37.05
MYS	4.70	4.88	0.19	3.94	3.99	5.44	1.45	36.40
MEX	5.33	5.77	0.44	8.31	4.53	6.11	1.58	34.99
SAU	7.85	8.15	0.30	3.81	6.67	8.92	2.25	33.70
THA	4.23	4.40	0.17	4.01	3.60	4.76	1.16	32.15
URY	6.59	6.96	0.37	5.58	5.60	7.28	1.68	29.94
BRA	5.57	5.88	0.32	5.68	4.73	6.14	1.41	29.83
LBY	8.28	8.53	0.25	2.98	7.04	9.08	2.04	29.01
ARM	6.51	6.67	0.16	2.48	5.53	7.12	1.59	28.67
CHL	5.54	5.86	0.32	5.73	4.71	6.06	1.35	28.63
TKM	8.08	8.22	0.14	1.68	6.87	8.82	1.94	28.30
ALB	5.69	5.90	0.22	3.78	4.84	6.18	1.35	27.87
LBN	6.97	7.09	0.12	1.77	5.92	7.47	1.55	26.11
RAP	9.98	10.31	0.33	3.26	8.48	10.45	1.97	23.21
PAN	5.25	5.51	0.26	4.92	4.47	5.50	1.03	23.07
TUR	9.70	9.95	0.24	2.52	8.25	10.04	1.79	21.72
AZE	9.22	9.39	0.16	1.76	7.84	9.52	1.68	21.44
ZAF	6.02	6.24	0.22	3.64	5.12	6.10	0.98	19.19
VEN	6.33	6.50	0.17	2.69	5.38	6.37	1.00	18.50
GSA	6.08	6.26	0.18	2.94	5.17	6.11	0.94	18.25
GNQ	7.51	7.70	0.19	2.50	6.39	7.55	1.16	18.23
CRI	6.72	6.97	0.25	3.65	5.71	6.73	1.02	17.87
OIO	6.83	7.03	0.20	2.94	5.80	6.77	0.96	16.59
DOM	5.74	5.84	0.10	1.69	4.88	5.61	0.74	15.08
DZA	7.80	7.97	0.18	2.27	6.63	7.62	0.99	14.98
LKA	8.28	8.37	0.09	1.14	7.03	8.07	1.04	14.78
PRY	5.42	5.57	0.15	2.74	4.61	5.26	0.65	14.05
MOR	7.40	7.58	0.18	2.45	6.29	7.12	0.83	13.18
ARG	6.23	6.37	0.14	2.27	5.29	5.99	0.70	13.17
FJI	6.32	6.41	0.08	1.34	5.37	6.08	0.70	13.05
COL	5.92	6.03	0.10	1.76	5.04	5.68	0.65	12.85
UZB	7.19	7.26	0.07	0.99	6.11	6.87	0.76	12.48
MNG	9.50	9.63	0.14	1.43	8.07	9.04	0.96	11.95
ECU	5.35	5.46	0.11	1.97	4.55	5.07	0.52	11.45
IDN	6.44	6.61	0.17	2.67	5.47	6.09	0.62	11.31
EGY	9.28	9.38	0.09	1.00	7.89	8.77	0.88	11.20

GAB	5.84	5.93	0.09	1.57	4.96	5.52	0.55	11.16
SLV	5.14	5.20	0.06	1.22	4.37	4.86	0.49	11.12
JOR	7.76	7.88	0.12	1.52	6.59	7.33	0.73	11.12
IRQ	9.87	9.97	0.10	1.02	8.39	9.30	0.91	10.91
IRN	13.24	13.35	0.12	0.88	11.25	12.46	1.21	10.72
PER	5.20	5.28	0.08	1.48	4.42	4.89	0.47	10.59
TUN	14.23	14.39	0.16	1.14	12.10	13.36	1.26	10.46
JAM	8.33	8.51	0.17	2.07	7.08	7.77	0.69	9.68
PHL	4.76	4.82	0.06	1.27	4.04	4.42	0.38	9.35
BTN	6.48	6.55	0.07	1.08	5.51	5.98	0.47	8.49
BLZ	4.63	4.68	0.06	1.20	3.93	4.22	0.28	7.23
BWA	9.00	9.10	0.11	1.18	7.65	8.12	0.48	6.24
VNM	5.74	5.83	0.10	1.66	4.88	5.17	0.29	5.93
BOL	6.17	6.23	0.05	0.86	5.25	5.55	0.31	5.83
IND	8.07	8.12	0.05	0.63	6.86	7.25	0.39	5.67
NAM	7.80	7.89	0.09	1.16	6.63	6.98	0.35	5.32
KGZ	8.87	8.90	0.04	0.42	7.54	7.93	0.40	5.25
NIC	7.00	7.05	0.05	0.70	5.95	6.26	0.30	5.13
GTM	5.47	5.51	0.04	0.68	4.65	4.87	0.22	4.72
HND	6.05	6.08	0.04	0.58	5.14	5.36	0.22	4.28
COG	6.52	6.56	0.04	0.62	5.54	5.77	0.23	4.19
LAO	5.65	5.68	0.03	0.52	4.80	5.00	0.20	4.10
KHM	6.24	6.26	0.02	0.37	5.30	5.46	0.16	2.92
TJK	9.95	9.98	0.03	0.27	8.46	8.69	0.23	2.74
CIV	6.23	6.26	0.03	0.44	5.29	5.42	0.13	2.47
AGO	9.82	9.85	0.03	0.35	8.35	8.55	0.20	2.45
SLB	5.76	5.79	0.03	0.51	4.90	5.01	0.11	2.33
PNG	4.24	4.26	0.02	0.43	3.61	3.69	0.08	2.32
DJI	8.32	8.35	0.04	0.45	7.07	7.23	0.16	2.25
TLS	4.72	4.74	0.01	0.29	4.02	4.10	0.09	2.20
HTI	6.64	6.67	0.03	0.43	5.65	5.76	0.11	2.01
SWZ	7.73	7.76	0.03	0.36	6.57	6.70	0.13	1.94
GHA	10.71	10.75	0.04	0.34	9.10	9.27	0.17	1.90
LSO	7.77	7.80	0.03	0.45	6.60	6.72	0.12	1.87
PAK	10.09	10.11	0.02	0.21	8.57	8.73	0.16	1.83
SLE	7.14	7.16	0.03	0.36	6.06	6.17	0.11	1.78
YEM	9.58	9.60	0.02	0.17	8.15	8.29	0.14	1.72
CMR	6.97	6.99	0.02	0.26	5.92	6.01	0.09	1.52
NPL	7.88	7.90	0.01	0.19	6.70	6.79	0.09	1.42
NGA	11.05	11.07	0.02	0.21	9.39	9.52	0.12	1.32
GNB	5.39	5.40	0.01	0.22	4.58	4.64	0.06	1.25
AFG	8.06	8.08	0.01	0.15	6.85	6.94	0.09	1.25
SDN	11.29	11.32	0.03	0.23	9.60	9.70	0.11	1.11
BGD	7.87	7.89	0.01	0.15	6.69	6.77	0.07	1.10
MRT	8.66	8.67	0.01	0.17	7.36	7.43	0.08	1.04
BEN	6.65	6.66	0.01	0.17	5.65	5.71	0.06	1.02
BFA	6.40	6.41	0.01	0.18	5.44	5.49	0.05	0.99
SEN	6.56	6.58	0.01	0.17	5.58	5.63	0.05	0.93
ZMB	9.34	9.35	0.02	0.17	7.94	8.01	0.07	0.90

SYR	9.08	9.09	0.01	0.10	7.72	7.79	0.06	0.83
TGO	6.36	6.37	0.01	0.14	5.40	5.45	0.04	0.79
MLI	7.89	7.90	0.01	0.12	6.70	6.75	0.05	0.74
GMB	7.06	7.07	0.01	0.12	6.00	6.04	0.04	0.72
UGA	7.86	7.87	0.01	0.14	6.68	6.72	0.05	0.68
TZA	9.61	9.62	0.01	0.12	8.17	8.22	0.05	0.64
LBR	6.98	6.99	0.01	0.12	5.93	5.97	0.04	0.61
TCD	7.40	7.41	0.01	0.09	6.29	6.33	0.04	0.58
GIN	13.65	13.66	0.01	0.09	11.60	11.67	0.07	0.57
KEN	11.21	11.22	0.01	0.10	9.53	9.58	0.05	0.53
MDG	8.15	8.15	0.01	0.10	6.92	6.96	0.03	0.50
NER	6.78	6.79	0.01	0.07	5.77	5.79	0.02	0.41
MWI	8.32	8.33	0.01	0.09	7.08	7.10	0.03	0.41
CAF	7.18	7.18	0.00	0.06	6.10	6.12	0.02	0.38
MOZ	7.28	7.28	0.01	0.08	6.19	6.21	0.02	0.37
COD	9.48	9.48	0.01	0.06	8.05	8.08	0.03	0.34
BDI	9.59	9.59	0.01	0.06	8.15	8.17	0.02	0.29
ERI	9.09	9.09	0.00	0.05	7.72	7.74	0.02	0.24
MMR	8.32	8.33	0.00	0.05	7.07	7.09	0.01	0.19
ETH	14.20	14.21	0.01	0.04	12.07	12.09	0.02	0.17

Table A11. Consumption of red and processed meat consumption in the reference (REF) and tax (TAX) scenarios in the year 2020 by region and country (abs: absolute values in grams per person per day (g/d), chg: changes between scenarios in g/d, pct: percentage change between scenarios). Countries are sorted by greatest percentage changes for processed meat in the TAX scenario.

Region	Red meat				Processed meat			
	REF	TAX			REF	TAX		
	abs	abs	chg	pct	abs	abs	chg	pct
Global	56.65	56.76	0.11	0.20	16.52	13.90	-2.62	-15.87
HIC	94.91	94.13	-0.78	-0.82	48.14	36.06	-12.09	-25.11
UMC	65.97	66.07	0.09	0.14	25.99	22.29	-3.71	-14.25
LMC	53.48	53.86	0.38	0.72	8.88	8.31	-0.57	-6.45
LIC	25.70	25.71	0.01	0.04	6.77	6.69	-0.08	-1.17
AFR_LMIC	24.02	24.03	0.01	0.05	4.21	4.15	-0.06	-1.41
AMR_LMIC	67.61	67.19	-0.42	-0.62	28.35	25.14	-3.21	-11.33
EMR_LMIC	26.70	26.79	0.09	0.33	5.68	5.60	-0.08	-1.48
EUR_LMIC	55.58	56.72	1.14	2.05	26.30	21.70	-4.60	-17.48
SEA_LMIC	6.35	6.37	0.02	0.39	7.23	6.97	-0.25	-3.53
WPR_LMIC	117.05	117.93	0.87	0.75	11.80	10.80	-0.99	-8.42
SWE	117.50	115.58	-1.92	-1.63	27.16	17.16	-10.00	-36.81
DEU	106.38	102.81	-3.57	-3.36	51.73	32.72	-19.01	-36.74
AUT	130.79	125.37	-5.42	-4.14	76.99	48.78	-28.21	-36.64
NOR	86.26	83.13	-3.13	-3.63	32.44	21.24	-11.20	-34.53
CHP	95.38	92.53	-2.85	-2.99	35.57	23.46	-12.11	-34.05
FNP	75.52	76.21	0.69	0.92	50.26	33.50	-16.76	-33.34
SVK	69.22	70.86	1.65	2.38	40.31	27.01	-13.30	-33.00
CZE	93.11	94.92	1.80	1.94	45.61	31.52	-14.08	-30.88

DNK	125.73	119.59	-6.14	-4.88	66.99	47.44	-19.55	-29.18
NLD	79.05	75.56	-3.49	-4.42	45.24	32.24	-13.00	-28.74
BLT	76.39	78.45	2.07	2.70	34.00	24.52	-9.47	-27.87
BLX	88.06	86.24	-1.82	-2.06	42.95	31.35	-11.59	-27.00
RUS	61.48	62.91	1.43	2.33	25.74	18.82	-6.92	-26.90
ITP	113.76	111.51	-2.25	-1.98	43.09	31.66	-11.43	-26.52
HUN	64.10	64.78	0.68	1.06	55.68	41.14	-14.53	-26.10
USA	99.60	98.80	-0.80	-0.80	79.19	59.30	-19.89	-25.12
POL	79.26	78.96	-0.30	-0.38	61.79	46.73	-15.06	-24.38
PRT	115.61	112.45	-3.17	-2.74	25.31	19.32	-5.99	-23.66
IRL	106.38	106.60	0.22	0.20	59.43	46.01	-13.42	-22.58
GRC	131.66	133.80	2.14	1.63	7.48	5.80	-1.69	-22.55
CAN	110.16	111.36	1.20	1.09	31.57	24.52	-7.05	-22.34
SPP	137.20	135.25	-1.95	-1.42	41.16	31.98	-9.18	-22.31
HRV	72.81	73.50	0.69	0.95	26.88	20.98	-5.90	-21.95
UKP	78.89	78.55	-0.34	-0.43	47.38	37.05	-10.32	-21.78
FRP	85.38	83.03	-2.35	-2.75	61.94	49.77	-12.17	-19.65
JPN	63.85	63.82	-0.04	-0.06	9.98	8.08	-1.90	-19.05
KAZ	86.11	87.58	1.48	1.71	36.84	29.91	-6.93	-18.81
CUB	32.96	33.05	0.09	0.27	7.90	6.44	-1.46	-18.48
AUS	151.94	154.52	2.58	1.70	31.81	25.98	-5.84	-18.35
SVN	116.58	116.11	-0.47	-0.40	46.50	38.82	-7.67	-16.50
CRB	48.34	48.87	0.53	1.10	18.33	15.41	-2.92	-15.94
NZL	108.13	108.33	0.21	0.19	54.06	45.46	-8.60	-15.91
BGR	53.99	55.66	1.67	3.09	28.43	24.23	-4.20	-14.79
MEX	41.16	40.44	-0.71	-1.74	43.41	36.99	-6.41	-14.78
ISL	82.94	83.38	0.43	0.52	42.76	36.74	-6.02	-14.08
BRA	105.48	104.49	-0.99	-0.94	30.10	25.89	-4.21	-13.98
BLR	74.52	77.33	2.81	3.77	56.96	49.76	-7.20	-12.64
OSA	106.40	111.00	4.60	4.32	31.84	27.88	-3.96	-12.44
OBN	27.50	27.62	0.13	0.45	10.77	9.46	-1.30	-12.11
ISR	59.13	59.57	0.44	0.74	12.94	11.39	-1.55	-11.99
CHM	129.09	130.10	1.01	0.78	9.44	8.37	-1.07	-11.32
ROU	72.38	73.98	1.60	2.21	29.76	26.47	-3.29	-11.05
GEO	40.38	40.88	0.50	1.24	38.76	34.90	-3.86	-9.97
ALB	63.55	63.92	0.37	0.58	22.74	20.49	-2.25	-9.91
PAN	39.02	38.79	-0.24	-0.60	50.87	45.93	-4.95	-9.72
GNQ	1.29	1.29	0.00	-0.02	0.22	0.20	-0.02	-9.63
URY	97.90	98.14	0.24	0.24	25.55	23.19	-2.37	-9.26
UKR	31.84	32.74	0.90	2.82	29.94	27.20	-2.74	-9.15
GSA	11.78	11.77	-0.02	-0.14	3.29	3.00	-0.29	-8.83
CHL	89.57	89.69	0.12	0.13	23.47	21.42	-2.05	-8.74
TKM	113.26	115.12	1.87	1.65	35.00	32.02	-2.99	-8.53
MDA	20.76	21.21	0.45	2.18	22.63	20.73	-1.90	-8.42
CRI	27.23	27.11	-0.12	-0.43	37.82	34.77	-3.06	-8.09
DOM	38.74	38.85	0.12	0.30	13.43	12.41	-1.02	-7.63
PRY	84.60	84.19	-0.41	-0.48	29.58	27.35	-2.23	-7.53
SAU	27.72	28.15	0.43	1.56	1.85	1.71	-0.14	-7.49
LBY	38.11	38.64	0.53	1.40	1.73	1.61	-0.12	-7.20

KOR	128.76	130.91	2.15	1.67	13.80	12.84	-0.96	-6.99
ARM	38.73	39.34	0.61	1.57	31.81	29.66	-2.16	-6.78
THA	38.85	39.43	0.58	1.48	15.02	14.00	-1.01	-6.75
TUR	25.31	25.53	0.22	0.88	2.52	2.36	-0.17	-6.56
MYS	26.81	27.32	0.52	1.93	22.34	20.88	-1.46	-6.52
ZAF	64.71	64.81	0.10	0.16	10.04	9.40	-0.65	-6.43
RAP	45.81	46.18	0.38	0.82	3.12	2.92	-0.20	-6.34
GAB	41.53	41.54	0.00	0.01	5.71	5.36	-0.35	-6.06
VEN	36.29	36.49	0.20	0.55	27.20	25.61	-1.59	-5.86
AZE	31.36	31.74	0.38	1.21	21.79	20.54	-1.24	-5.70
ECU	56.11	56.05	-0.06	-0.11	26.21	24.72	-1.49	-5.67
CYP	109.61	112.69	3.09	2.82	17.06	16.10	-0.96	-5.63
COL	36.74	36.83	0.09	0.25	22.27	21.06	-1.21	-5.45
LKA	4.56	4.59	0.04	0.77	3.42	3.23	-0.19	-5.44
PER	17.72	17.73	0.02	0.10	8.54	8.09	-0.45	-5.24
SLV	10.54	10.57	0.04	0.34	18.88	17.92	-0.96	-5.09
EGY	35.00	35.16	0.16	0.47	2.85	2.70	-0.14	-5.01
MNG	223.67	224.52	0.85	0.38	59.92	57.05	-2.87	-4.78
OIO	9.69	9.73	0.04	0.44	4.98	4.75	-0.23	-4.61
UZB	49.17	49.50	0.32	0.66	17.72	16.91	-0.81	-4.58
ARG	143.49	143.89	0.40	0.28	25.19	24.10	-1.08	-4.31
LBN	76.09	77.45	1.36	1.79	8.81	8.46	-0.35	-3.94
BLZ	29.90	29.88	-0.02	-0.08	16.33	15.69	-0.64	-3.94
IND	2.36	2.36	0.00	0.04	7.24	6.96	-0.28	-3.85
BWA	42.30	42.21	-0.09	-0.22	11.24	10.82	-0.42	-3.76
IRQ	7.90	7.95	0.05	0.57	0.50	0.48	-0.02	-3.52
FJI	51.96	52.34	0.38	0.73	19.65	19.01	-0.64	-3.24
DZA	34.95	35.19	0.25	0.70	0.97	0.94	-0.03	-3.16
NAM	43.84	43.72	-0.12	-0.27	12.84	12.43	-0.41	-3.16
BTN	26.68	26.77	0.09	0.33	14.11	13.70	-0.41	-2.92
BOL	74.92	74.96	0.04	0.05	27.92	27.10	-0.81	-2.91
MOR	31.80	31.96	0.16	0.50	2.53	2.45	-0.07	-2.89
JAM	21.17	21.20	0.03	0.15	13.39	13.01	-0.38	-2.83
IRN	30.29	30.49	0.20	0.67	3.43	3.34	-0.09	-2.71
TUN	38.43	38.65	0.22	0.58	2.32	2.26	-0.06	-2.63
IDN	10.26	10.29	0.03	0.26	7.09	6.90	-0.18	-2.58
COG	13.46	13.45	0.00	-0.03	2.08	2.03	-0.05	-2.54
GTM	13.01	13.01	0.00	0.03	15.53	15.13	-0.39	-2.53
NIC	8.08	8.09	0.01	0.10	9.63	9.40	-0.23	-2.38
PHL	47.25	47.47	0.22	0.46	24.33	23.77	-0.56	-2.29
JOR	37.33	37.56	0.23	0.62	1.56	1.52	-0.03	-2.13
HND	15.59	15.61	0.01	0.08	21.10	20.67	-0.43	-2.04
KGZ	69.32	69.53	0.22	0.31	21.42	21.05	-0.37	-1.73
VNM	72.29	72.27	-0.01	-0.02	28.14	27.66	-0.48	-1.71
CIV	10.10	10.09	-0.01	-0.06	1.32	1.30	-0.02	-1.54
DJI	41.44	41.39	-0.05	-0.11	6.52	6.42	-0.10	-1.49
AGO	43.73	43.73	0.00	0.00	7.69	7.58	-0.11	-1.47
SWZ	60.48	60.44	-0.04	-0.07	11.43	11.29	-0.14	-1.25
GHA	12.20	12.20	-0.01	-0.05	2.03	2.00	-0.02	-1.18

LSO	31.67	31.63	-0.04	-0.12	6.87	6.79	-0.08	-1.14
SLE	6.80	6.80	0.00	-0.07	1.23	1.22	-0.01	-1.10
HTI	21.28	21.27	-0.01	-0.06	5.84	5.77	-0.06	-1.07
LAO	36.92	37.01	0.08	0.22	17.04	16.86	-0.18	-1.05
PAK	17.29	17.30	0.01	0.04	10.01	9.91	-0.10	-1.04
TJK	23.73	23.76	0.03	0.15	7.28	7.21	-0.07	-0.92
CMR	16.90	16.90	0.00	-0.02	7.69	7.62	-0.07	-0.91
NGA	18.48	18.48	0.00	-0.02	3.99	3.95	-0.03	-0.85
GNB	33.72	33.71	-0.01	-0.03	5.73	5.69	-0.05	-0.79
KHM	43.31	43.38	0.07	0.16	6.35	6.30	-0.05	-0.75
TLS	34.49	34.52	0.03	0.10	10.10	10.03	-0.07	-0.70
BFA	38.29	38.28	-0.01	-0.03	8.18	8.12	-0.05	-0.65
MRT	62.99	62.99	-0.01	-0.01	6.67	6.63	-0.04	-0.63
BEN	9.87	9.86	0.00	-0.01	2.95	2.93	-0.02	-0.62
YEM	13.88	13.89	0.01	0.09	0.90	0.90	-0.01	-0.59
SEN	22.15	22.14	-0.01	-0.03	4.15	4.12	-0.02	-0.58
NPL	22.63	22.64	0.01	0.05	6.86	6.82	-0.04	-0.57
AFG	29.22	29.23	0.01	0.04	4.17	4.15	-0.02	-0.57
ZMB	20.93	20.92	-0.01	-0.03	4.30	4.27	-0.02	-0.55
BGD	8.23	8.24	0.00	0.02	1.73	1.72	-0.01	-0.55
CAF	69.77	69.74	-0.03	-0.04	10.56	10.51	-0.05	-0.51
TGO	12.64	12.64	0.00	-0.02	2.68	2.67	-0.01	-0.50
SDN	50.15	50.15	0.00	0.00	8.57	8.53	-0.04	-0.47
MLI	35.53	35.53	0.00	-0.01	4.27	4.25	-0.02	-0.47
GMB	12.92	12.92	0.00	-0.02	2.65	2.64	-0.01	-0.45
UGA	24.25	24.24	-0.01	-0.03	5.06	5.04	-0.02	-0.43
TZA	22.88	22.88	0.00	-0.02	4.62	4.60	-0.02	-0.42
LBR	12.55	12.54	0.00	-0.03	2.40	2.39	-0.01	-0.39
TCD	29.55	29.55	0.00	-0.01	3.31	3.29	-0.01	-0.38
PNG	36.57	36.62	0.05	0.13	9.35	9.32	-0.03	-0.37
GIN	19.43	19.43	0.00	-0.01	3.19	3.18	-0.01	-0.36
KEN	34.39	34.39	-0.01	-0.02	5.65	5.63	-0.02	-0.35
SLB	23.17	23.20	0.03	0.12	7.76	7.74	-0.03	-0.34
MDG	22.47	22.46	0.00	-0.02	4.27	4.25	-0.01	-0.32
SYR	37.41	37.43	0.01	0.04	2.66	2.65	-0.01	-0.30
NER	57.18	57.17	-0.01	-0.01	7.25	7.23	-0.02	-0.28
MWI	12.06	12.06	0.00	-0.02	2.67	2.66	-0.01	-0.25
MOZ	25.87	25.87	0.00	-0.02	5.69	5.68	-0.01	-0.21
COD	3.37	3.37	0.00	-0.01	0.41	0.41	0.00	-0.21
BDI	8.88	8.88	0.00	-0.01	2.34	2.34	0.00	-0.18
ERI	12.03	12.03	0.00	-0.01	2.38	2.38	0.00	-0.15
ETH	16.92	16.91	0.00	-0.01	2.82	2.82	0.00	-0.11
MMR	29.81	29.81	0.00	0.00	16.36	16.35	-0.01	-0.05

Table A12. Consumption of red and processed meat substitutes in the tax (TAX) scenario in the year 2020 by region and country (chg: changes with respect to the REF scenario in g/d, pct: percentage with respect to the REF scenario). Countries are sorted by greatest percentage changes in poultry consumption.

Region	Poultry		Milk		Eggs		Oils	
	chg	pct	chg	pct	chg	pct	chg	pct
Global	1.76	4.89	1.05	0.44	0.10	0.42	-0.11	-0.37
HIC	7.15	9.29	4.72	0.91	0.28	0.87	-0.44	-0.88
UMC	2.43	3.49	1.31	0.39	0.11	0.41	-0.12	-0.35
LMC	0.66	2.69	0.29	0.16	0.08	0.30	-0.05	-0.19
LIC	0.02	0.21	0.02	0.03	0.00	0.03	0.00	-0.02
AFR_LMIC	0.10	0.90	0.04	0.05	0.00	0.05	-0.01	-0.03
AMR_LMIC	1.80	2.46	0.73	0.24	0.06	0.25	-0.08	-0.23
EMR_LMIC	0.19	0.87	0.11	0.04	0.01	0.07	-0.01	-0.06
EUR_LMIC	2.93	7.06	2.45	0.57	0.19	0.66	-0.18	-0.60
SEA_LMIC	0.12	0.92	0.10	0.06	0.01	0.09	-0.02	-0.07
WPR_LMIC	1.43	3.58	0.51	0.37	0.17	0.37	-0.10	-0.36
NOR	3.96	12.59	7.80	1.19	0.30	1.19	-0.47	-1.18
SWE	4.42	12.30	10.82	1.17	0.31	1.17	-0.50	-1.15
USA	13.97	12.00	6.94	1.14	0.38	1.14	-0.68	-1.13
AUT	5.54	11.91	6.37	1.13	0.36	1.13	-0.58	-1.12
DEU	4.30	11.66	7.34	1.11	0.32	1.11	-0.51	-1.10
CHP	4.05	11.47	8.34	1.09	0.26	1.09	-0.51	-1.08
SVK	6.30	10.51	3.09	1.00	0.33	1.00	-0.32	-0.99
FNP	4.59	9.95	8.08	0.95	0.19	0.95	-0.29	-0.94
DNK	4.49	9.82	6.14	0.94	0.44	0.94	-0.17	-0.93
CZE	7.30	9.72	4.78	0.93	0.22	0.93	-0.44	-0.92
CAN	8.91	9.68	4.39	0.93	0.24	0.93	-0.52	-0.92
NLD	4.75	9.52	8.15	0.91	0.40	0.91	-0.41	-0.90
RUS	4.88	9.04	3.48	0.87	0.33	0.87	-0.28	-0.86
BLX	4.94	8.50	5.04	0.82	0.25	0.82	-0.43	-0.81
AUS	8.48	8.49	4.34	0.82	0.10	0.82	-0.46	-0.81
OSA	10.74	8.34	2.46	0.80	0.34	0.80	-0.26	-0.80
BLT	4.98	8.31	5.06	0.80	0.25	0.80	-0.23	-0.80
BGR	4.70	8.21	3.32	0.79	0.25	0.79	-0.34	-0.79
ITP	2.88	8.19	5.27	0.79	0.22	0.79	-0.47	-0.78
JPN	3.45	7.98	1.60	0.77	0.37	0.77	-0.27	-0.76
HUN	6.37	7.78	3.25	0.75	0.31	0.75	-0.32	-0.75
POL	4.58	7.56	3.34	0.73	0.22	0.73	-0.25	-0.73
PRT	4.15	7.32	3.86	0.71	0.16	0.71	-0.31	-0.70
GRC	2.83	7.26	4.92	0.70	0.16	0.70	-0.38	-0.70
HRV	1.70	6.70	3.37	0.65	0.18	0.65	-0.23	-0.65
UKP	5.01	6.60	4.02	0.64	0.16	0.64	-0.27	-0.64
IRL	4.55	6.51	4.05	0.63	0.11	0.63	-0.24	-0.63
FRP	3.35	6.37	4.20	0.62	0.21	0.62	-0.29	-0.62
SPP	3.96	6.34	2.64	0.62	0.22	0.62	-0.40	-0.61
CRB	7.10	6.00	1.62	0.58	0.07	0.58	-0.14	-0.58
BLR	2.84	5.75	2.41	0.56	0.22	0.56	-0.19	-0.56
SVN	3.14	5.61	3.42	0.55	0.09	0.55	-0.18	-0.54
NZL	5.10	5.58	1.20	0.54	0.13	0.54	-0.12	-0.54

ROU	3.07	5.27	3.32	0.52	0.19	0.52	-0.18	-0.51
KAZ	2.19	4.76	2.72	0.47	0.08	0.47	-0.13	-0.46
CYP	3.11	4.72	1.53	0.46	0.10	0.46	-0.14	-0.46
KOR	2.03	4.57	0.31	0.45	0.14	0.45	-0.18	-0.45
ISR	8.59	4.53	2.08	0.44	0.10	0.44	-0.30	-0.44
ISL	2.13	4.19	2.49	0.41	0.08	0.41	-0.08	-0.41
UKR	1.83	4.13	1.87	0.41	0.15	0.41	-0.14	-0.40
CHM	1.60	3.87	0.59	0.38	0.20	0.38	-0.12	-0.38
OBN	0.58	3.80	1.02	0.37	0.04	0.37	-0.07	-0.37
MDA	1.90	3.79	1.40	0.37	0.09	0.37	-0.13	-0.37
CUB	1.15	3.56	0.73	0.35	0.06	0.35	-0.07	-0.35
MEX	2.60	3.48	0.99	0.34	0.15	0.34	-0.09	-0.34
GEO	1.03	3.30	1.50	0.33	0.06	0.33	-0.08	-0.32
MYS	3.42	3.19	0.38	0.31	0.11	0.31	-0.15	-0.31
SAU	3.07	2.99	0.69	0.30	0.04	0.30	-0.11	-0.29
THA	1.15	2.90	0.22	0.29	0.08	0.29	-0.06	-0.29
BRA	2.65	2.89	1.11	0.29	0.06	0.29	-0.13	-0.28
URY	1.37	2.89	1.13	0.29	0.09	0.29	-0.07	-0.28
CHL	2.44	2.81	0.75	0.28	0.05	0.28	-0.07	-0.28
LBY	1.36	2.59	0.60	0.26	0.08	0.26	-0.06	-0.26
ALB	0.68	2.58	1.95	0.25	0.04	0.25	-0.04	-0.25
ARM	0.56	2.52	1.04	0.25	0.06	0.25	-0.04	-0.25
TKM	0.38	2.42	1.03	0.24	0.05	0.24	-0.05	-0.24
PAN	2.61	2.33	0.40	0.23	0.04	0.23	-0.07	-0.23
LBN	1.89	2.27	0.73	0.22	0.05	0.22	-0.09	-0.22
RAP	2.99	2.19	0.62	0.22	0.07	0.22	-0.04	-0.22
TUR	0.84	2.01	0.67	0.20	0.05	0.20	-0.10	-0.20
AZE	0.41	1.92	0.51	0.19	0.03	0.19	-0.02	-0.19
ZAF	1.48	1.92	0.34	0.19	0.03	0.19	-0.06	-0.19
CRI	1.18	1.82	0.79	0.18	0.05	0.18	-0.07	-0.18
GSA	1.08	1.78	0.35	0.18	0.01	0.18	-0.03	-0.18
VEN	1.37	1.78	0.37	0.18	0.02	0.18	-0.07	-0.18
GNQ	0.02	1.74			0.00	0.17		
OIO	0.42	1.66	0.23	0.16	0.02	0.16	-0.03	-0.16
DZA	0.34	1.47	0.47	0.15	0.02	0.15	-0.05	-0.15
PRY	0.34	1.44	0.24	0.14	0.07	0.14	-0.06	-0.14
DOM	1.46	1.43	0.20	0.14	0.02	0.14	-0.07	-0.14
LKA	0.30	1.35	0.15	0.13	0.01	0.13	-0.01	-0.13
MOR	0.58	1.34	0.15	0.13	0.03	0.13	-0.04	-0.13
ARG	1.07	1.32	0.62	0.13	0.03	0.13	-0.05	-0.13
COL	0.67	1.25	0.41	0.12	0.03	0.12	-0.04	-0.12
FJI	0.61	1.23	0.12	0.12	0.01	0.12	-0.04	-0.12
IDN	0.33	1.21	0.04	0.12	0.02	0.12	-0.03	-0.12
ECU	0.65	1.16	0.28	0.12	0.02	0.12	-0.05	-0.12
MNG	0.02	1.15	0.33	0.11	0.00	0.11	-0.03	-0.11
UZB	0.06	1.15	0.44	0.11	0.01	0.11	-0.03	-0.11
GAB	0.75	1.10	0.11	0.11	0.00	0.11	-0.02	-0.11
JOR	0.84	1.09	0.24	0.11	0.02	0.11	-0.05	-0.11
SLV	0.48	1.06	0.30	0.11	0.03	0.11	-0.02	-0.11

EGY	0.31	1.05	0.14	0.10	0.01	0.10	-0.01	-0.10
PER	0.37	1.04	0.14	0.10	0.02	0.10	-0.02	-0.10
IRQ	0.10	1.03	0.09	0.10	0.01	0.10	-0.05	-0.10
JAM	1.32	1.02	0.26	0.10	0.01	0.10	-0.03	-0.10
IRN	0.49	1.00	0.20	0.10	0.02	0.10	-0.03	-0.10
TUN	0.37	1.00	0.26	0.10	0.02	0.10	-0.05	-0.10
PHL	0.25	0.92	0.04	0.09	0.01	0.09	-0.01	-0.09
BTN	0.02	0.83	0.12	0.08	0.00	0.08		
BLZ	0.54	0.74	0.17	0.07	0.01	0.07	-0.01	-0.07
VNM	0.15	0.67	0.02	0.07	0.01	0.07	0.00	-0.07
BWA	0.08	0.65	0.17	0.07	0.00	0.07	-0.02	-0.07
BOL	0.32	0.59	0.06	0.06	0.01	0.06	-0.01	-0.06
NAM	0.18	0.57	0.12	0.06	0.00	0.06	-0.01	-0.06
IND	0.04	0.55	0.13	0.06	0.00	0.06	-0.01	-0.06
NIC	0.18	0.51	0.11	0.05	0.00	0.05	-0.01	-0.05
KGZ	0.05	0.50	0.25	0.05	0.00	0.05	-0.01	-0.05
GTM	0.21	0.48	0.06	0.05	0.02	0.05	-0.01	-0.05
COG	0.14	0.43	0.03	0.04	0.00	0.04	-0.02	-0.04
HND	0.25	0.43	0.12	0.04	0.01	0.04	-0.01	-0.04
LAO	0.08	0.41	0.01	0.04	0.00	0.04	0.00	-0.04
KHM	0.03	0.29	0.00	0.03	0.00	0.03	0.00	-0.03
TJK	0.01	0.27	0.05	0.03	0.00	0.03	-0.01	-0.03
CIV	0.01	0.26	0.01	0.03	0.00	0.03	-0.01	-0.03
AGO	0.08	0.25	0.01	0.02	0.00	0.02	-0.01	-0.02
PNG	0.01	0.25	0.00	0.02	0.00	0.02		
SLB	0.01	0.25	0.00	0.03	0.00	0.03	-0.01	-0.03
DJI	0.04	0.24	0.04	0.02	0.00	0.02	-0.01	-0.02
HTI	0.02	0.22	0.01	0.02	0.00	0.02	0.00	-0.02
TLS	0.05	0.22	0.00	0.02	0.00	0.02	0.00	-0.02
LSO	0.03	0.21	0.01	0.02	0.00	0.02	0.00	-0.02
SWZ	0.04	0.21	0.04	0.02	0.00	0.02	0.00	-0.02
GHA	0.04	0.20	0.01	0.02	0.00	0.02	0.00	-0.02
SLE	0.02	0.19	0.00	0.02	0.00	0.02	-0.01	-0.02
PAK	0.01	0.18	0.08	0.02	0.00	0.02	-0.01	-0.02
YEM	0.04	0.17	0.02	0.02	0.00	0.02	0.00	-0.02
CMR	0.01	0.16	0.01	0.02	0.00	0.02	0.00	-0.02
NGA	0.01	0.14	0.00	0.01	0.00	0.01	-0.01	-0.01
NPL	0.00	0.14	0.02	0.01	0.00	0.01	0.00	-0.01
AFG	0.01	0.13	0.02	0.01	0.00	0.01	0.00	-0.01
GNB	0.01	0.13	0.01	0.01	0.00	0.01	0.00	-0.01
SDN	0.00	0.12	0.06	0.01	0.00	0.01	0.00	-0.01
BEN	0.03	0.11	0.00	0.01	0.00	0.01	0.00	-0.01
BFA	0.01	0.11	0.01	0.01	0.00	0.01	0.00	-0.01
BGD	0.01	0.11	0.01	0.01	0.00	0.01	0.00	-0.01
MRT	0.01	0.11	0.04	0.01	0.00	0.01	0.00	-0.01
SEN	0.01	0.10	0.01	0.01	0.00	0.01	0.00	-0.01
ZMB	0.01	0.10	0.00	0.01	0.00	0.01	0.00	-0.01
GMB	0.01	0.08	0.01	0.01	0.00	0.01	0.00	-0.01
MLI	0.01	0.08	0.01	0.01	0.00	0.01	0.00	-0.01

SYR	0.03	0.08	0.03	0.01	0.00	0.01	0.00	-0.01
TGO	0.01	0.08	0.00	0.01	0.00	0.01	0.00	-0.01
LBR	0.01	0.07	0.00	0.01	0.00	0.01	0.00	-0.01
TZA	0.00	0.07	0.01	0.01	0.00	0.01	0.00	-0.01
UGA	0.00	0.07	0.01	0.01	0.00	0.01	0.00	-0.01
GIN	0.00	0.06	0.00	0.01	0.00	0.01	0.00	-0.01
KEN	0.00	0.06	0.01	0.01	0.00	0.01	0.00	-0.01
TCD	0.00	0.06	0.00	0.01	0.00	0.01	0.00	-0.01
MDG	0.01	0.05	0.00	0.01	0.00	0.01	0.00	-0.01
CAF	0.00	0.04	0.00	0.00	0.00	0.00	0.00	0.00
COD	0.00	0.04	0.00	0.00	0.00	0.00		
MOZ	0.00	0.04	0.00	0.00	0.00	0.00	0.00	0.00
MWI	0.00	0.04	0.00	0.00	0.00	0.00	0.00	0.00
NER	0.00	0.04	0.01	0.00	0.00	0.00	0.00	0.00
BDI	0.00	0.03	0.00	0.00	0.00	0.00	0.00	0.00
ERI	0.00	0.03	0.00	0.00	0.00	0.00	0.00	0.00
ETH	0.00	0.02	0.00	0.00	0.00	0.00	0.00	0.00
MMR	0.01	0.02	0.00	0.00	0.00	0.00	0.00	0.00

Table A13. Deaths attributable to red and processed meat consumption in the reference (REF) and tax (TAX) scenarios in the year 2020 by region and country (abs: absolute values in thousands, chg: changes between scenarios in thousands, pct: percentage change between scenarios). Countries are sorted by greatest percentage changes for processed meat in the TAX scenario.

Region	Red meat				Processed meat			
	REF	TAX			REF	TAX		
	abs	abs	chg	pct	abs	abs	chg	pct
Global	863.06	866.22	3.16	0.37	1533.21	1298.58	-234.63	-15.30
HIC	167.22	165.81	-1.41	-0.84	604.53	470.21	-134.32	-22.22
UMC	124.08	124.84	0.76	0.61	384.96	320.46	-64.50	-16.76
LMC	531.38	535.15	3.76	0.71	484.43	449.69	-34.74	-7.17
LIC	34.90	34.92	0.02	0.05	55.69	54.81	-0.88	-1.58
AFR_LMIC	26.35	26.38	0.03	0.11	22.58	22.05	-0.53	-2.36
AMR_LMIC	64.64	64.21	-0.43	-0.67	171.62	153.50	-18.12	-10.56
EMR_LMIC	15.73	15.78	0.05	0.34	25.99	25.54	-0.45	-1.74
EUR_LMIC	61.85	63.16	1.31	2.12	308.52	255.14	-53.38	-17.30
SEA_LMIC	21.72	21.82	0.09	0.43	148.75	143.43	-5.32	-3.58
WPR_LMIC	500.08	503.56	3.48	0.70	247.61	225.31	-22.31	-9.01
SWE	2.11	2.08	-0.03	-1.50	4.05	2.64	-1.41	-34.84
DEU	15.23	14.75	-0.47	-3.11	57.37	38.46	-18.91	-32.97
NOR	0.73	0.70	-0.02	-3.42	1.82	1.23	-0.59	-32.32
CHP	0.93	0.90	-0.03	-2.79	2.81	1.93	-0.89	-31.53
AUT	1.85	1.78	-0.07	-3.77	9.34	6.45	-2.89	-30.94
SVK	0.86	0.87	0.02	2.27	5.81	4.06	-1.76	-30.19
FNP	0.63	0.63	0.01	0.87	4.59	3.22	-1.37	-29.85
CZE	2.21	2.25	0.04	1.81	11.46	8.27	-3.19	-27.80
NLD	2.08	2.00	-0.09	-4.18	6.12	4.52	-1.59	-26.05
BLT	1.23	1.26	0.03	2.56	6.31	4.69	-1.62	-25.71
RUS	36.34	37.15	0.81	2.24	145.80	108.83	-36.97	-25.36

DNK	1.32	1.26	-0.06	-4.47	3.72	2.79	-0.94	-25.14
BLX	1.57	1.54	-0.03	-1.94	4.87	3.68	-1.19	-24.47
ITP	14.60	14.34	-0.27	-1.83	33.63	25.57	-8.06	-23.98
HUN	1.77	1.79	0.02	1.01	14.32	11.05	-3.26	-22.80
PRT	3.20	3.12	-0.08	-2.52	3.19	2.47	-0.71	-22.32
GRC	3.77	3.82	0.06	1.49	1.69	1.32	-0.37	-22.16
POL	8.49	8.46	-0.03	-0.36	45.78	36.13	-9.65	-21.08
CAN	4.96	5.00	0.05	1.00	11.65	9.25	-2.40	-20.61
HRV	0.93	0.94	0.01	0.90	2.68	2.13	-0.55	-20.54
USA	39.91	39.61	-0.30	-0.74	266.31	211.73	-54.58	-20.50
SPP	10.40	10.27	-0.13	-1.29	18.35	14.65	-3.71	-20.20
UKP	7.48	7.45	-0.03	-0.41	31.39	25.30	-6.10	-19.42
IRL	0.52	0.53	0.00	0.19	2.58	2.08	-0.50	-19.41
JPN	15.88	15.87	-0.01	-0.05	12.66	10.31	-2.36	-18.62
CUB	0.69	0.70	0.00	0.27	1.37	1.12	-0.25	-18.10
KAZ	3.39	3.44	0.05	1.62	15.33	12.71	-2.62	-17.08
FRP	8.16	7.95	-0.21	-2.59	28.50	23.69	-4.81	-16.88
AUS	4.39	4.46	0.07	1.52	7.37	6.13	-1.24	-16.86
CRB	0.34	0.34	0.00	1.06	0.82	0.69	-0.12	-15.08
SVN	0.49	0.49	0.00	-0.37	1.03	0.88	-0.15	-14.78
BGR	1.95	2.01	0.06	2.98	7.21	6.22	-0.99	-13.74
NZL	0.69	0.69	0.00	0.18	2.48	2.14	-0.34	-13.73
MEX	9.32	9.17	-0.16	-1.68	58.66	51.12	-7.54	-12.85
BRA	35.35	35.04	-0.31	-0.87	60.76	52.96	-7.79	-12.83
ISL	0.03	0.03	0.00	0.49	0.13	0.11	-0.02	-12.52
OBN	1.27	1.28	0.01	0.45	2.99	2.63	-0.35	-11.77
ISR	0.56	0.57	0.00	0.71	0.80	0.71	-0.09	-11.54
OSA	0.67	0.70	0.03	3.99	1.62	1.43	-0.18	-11.39
CHM	475.31	478.73	3.42	0.72	188.55	167.70	-20.85	-11.06
BLR	1.69	1.75	0.06	3.58	18.75	16.74	-2.01	-10.70
ROU	4.64	4.74	0.10	2.11	13.67	12.28	-1.39	-10.20
GNQ	0.00	0.00	0.00	-0.02	0.00	0.00	0.00	-9.62
ALB	0.64	0.64	0.00	0.56	1.60	1.45	-0.15	-9.32
GEO	0.59	0.60	0.01	1.21	5.07	4.62	-0.45	-8.93
GSA	0.04	0.04	0.00	-0.14	0.06	0.05	-0.01	-8.75
URY	0.57	0.57	0.00	0.22	0.87	0.80	-0.08	-8.63
UKR	4.71	4.84	0.13	2.77	64.09	58.73	-5.36	-8.37
PAN	0.19	0.19	0.00	-0.59	1.47	1.35	-0.12	-8.34
CHL	1.89	1.89	0.00	0.12	2.72	2.50	-0.22	-8.17
MDA	0.21	0.22	0.00	2.15	2.83	2.61	-0.22	-7.87
TKM	1.06	1.08	0.02	1.53	4.49	4.15	-0.34	-7.67
SAU	0.86	0.87	0.01	1.53	0.45	0.42	-0.03	-7.45
DOM	0.50	0.50	0.00	0.29	1.42	1.32	-0.10	-7.32
CRI	0.13	0.13	0.00	-0.43	1.33	1.23	-0.10	-7.20
LBY	0.30	0.31	0.00	1.36	0.12	0.11	-0.01	-7.16
PRY	0.94	0.93	0.00	-0.45	1.93	1.80	-0.13	-6.88
KOR	9.01	9.14	0.14	1.52	4.64	4.32	-0.31	-6.72
TUR	2.75	2.77	0.02	0.87	2.38	2.22	-0.15	-6.51
THA	4.92	4.99	0.07	1.44	13.31	12.45	-0.86	-6.45

RAP	0.45	0.45	0.00	0.79	0.27	0.25	-0.02	-6.27
ZAF	7.86	7.87	0.01	0.15	6.38	5.99	-0.40	-6.22
ARM	0.38	0.38	0.01	1.53	3.31	3.11	-0.20	-6.14
MYS	1.02	1.04	0.02	1.89	7.46	7.00	-0.45	-6.09
GAB	0.08	0.08	0.00	0.01	0.07	0.07	0.00	-5.95
LKA	0.20	0.20	0.00	0.77	1.36	1.29	-0.07	-5.38
VEN	1.48	1.49	0.01	0.53	8.50	8.04	-0.46	-5.36
AZE	0.45	0.46	0.01	1.19	3.63	3.44	-0.19	-5.33
CYP	0.16	0.17	0.00	2.58	0.20	0.19	-0.01	-5.33
ECU	0.95	0.95	0.00	-0.10	2.91	2.76	-0.15	-5.22
PER	0.37	0.37	0.00	0.10	1.35	1.28	-0.07	-5.10
COL	1.41	1.42	0.00	0.25	7.61	7.22	-0.39	-5.08
EGY	3.95	3.97	0.02	0.46	3.09	2.94	-0.15	-4.97
SLV	0.07	0.07	0.00	0.33	1.25	1.19	-0.06	-4.78
OIO	0.07	0.07	0.00	0.44	0.20	0.19	-0.01	-4.53
UZB	2.51	2.52	0.02	0.64	10.65	10.19	-0.46	-4.33
MNG	0.89	0.89	0.00	0.33	2.02	1.93	-0.08	-4.01
ARG	8.65	8.67	0.02	0.25	12.54	12.04	-0.50	-3.98
LBN	0.34	0.35	0.01	1.69	0.53	0.51	-0.02	-3.83
IND	4.29	4.29	0.00	0.04	102.28	98.43	-3.85	-3.76
BLZ	0.01	0.01	0.00	-0.08	0.04	0.04	0.00	-3.73
BWA	0.11	0.11	0.00	-0.21	0.14	0.14	-0.01	-3.63
IRQ	0.29	0.29	0.00	0.57	0.16	0.16	-0.01	-3.51
DZA	2.84	2.86	0.02	0.69	0.49	0.48	-0.02	-3.15
NAM	0.13	0.13	0.00	-0.26	0.18	0.18	-0.01	-3.03
FJI	0.14	0.14	0.00	0.70	0.39	0.38	-0.01	-3.02
MOR	2.72	2.73	0.01	0.48	1.22	1.19	-0.04	-2.87
BTN	0.02	0.02	0.00	0.32	0.07	0.07	0.00	-2.79
JAM	0.18	0.18	0.00	0.15	0.55	0.54	-0.01	-2.71
IRN	2.34	2.36	0.02	0.66	3.18	3.10	-0.09	-2.68
BOL	1.07	1.07	0.00	0.05	2.64	2.57	-0.07	-2.66
TUN	0.71	0.72	0.00	0.56	0.38	0.37	-0.01	-2.61
IDN	7.21	7.23	0.02	0.26	20.58	20.06	-0.52	-2.53
COG	0.07	0.07	0.00	-0.03	0.06	0.06	0.00	-2.52
GTM	0.15	0.16	0.00	0.03	1.32	1.29	-0.03	-2.40
NIC	0.06	0.06	0.00	0.10	0.55	0.54	-0.01	-2.31
JOR	0.33	0.33	0.00	0.60	0.10	0.10	0.00	-2.12
PHL	8.19	8.22	0.04	0.45	28.75	28.14	-0.61	-2.12
HND	0.09	0.09	0.00	0.08	1.12	1.10	-0.02	-1.91
KGZ	0.52	0.52	0.00	0.30	2.00	1.97	-0.03	-1.61
VNM	13.28	13.28	0.00	-0.02	18.44	18.15	-0.29	-1.59
CIV	0.27	0.27	0.00	-0.06	0.19	0.18	0.00	-1.53
DJI	0.05	0.05	0.00	-0.11	0.04	0.04	0.00	-1.46
AGO	0.91	0.91	0.00	0.00	0.98	0.97	-0.01	-1.44
SWZ	0.10	0.10	0.00	-0.07	0.09	0.09	0.00	-1.20
GHA	0.42	0.42	0.00	-0.05	0.36	0.36	0.00	-1.18
LSO	0.10	0.10	0.00	-0.11	0.10	0.10	0.00	-1.11
SLE	0.06	0.06	0.00	-0.07	0.06	0.06	0.00	-1.09
HTI	0.51	0.51	0.00	-0.06	0.65	0.64	-0.01	-1.05

PAK	3.65	3.65	0.00	0.04	15.02	14.87	-0.15	-1.01
LAO	0.27	0.27	0.00	0.22	0.84	0.83	-0.01	-0.99
TJK	0.27	0.27	0.00	0.15	0.80	0.79	-0.01	-0.90
CMR	0.40	0.40	0.00	-0.02	1.03	1.02	-0.01	-0.89
NGA	2.87	2.87	0.00	-0.02	3.36	3.33	-0.03	-0.84
GNB	0.08	0.08	0.00	-0.03	0.07	0.07	0.00	-0.77
KHM	0.63	0.64	0.00	0.16	0.67	0.67	0.00	-0.73
TLS	0.03	0.03	0.00	0.10	0.07	0.07	0.00	-0.68
BFA	0.72	0.72	0.00	-0.03	0.82	0.82	-0.01	-0.64
MRT	0.24	0.24	0.00	-0.01	0.14	0.14	0.00	-0.61
BEN	0.13	0.13	0.00	-0.01	0.21	0.21	0.00	-0.61
YEM	0.32	0.32	0.00	0.09	0.17	0.17	0.00	-0.59
SEN	0.27	0.27	0.00	-0.03	0.27	0.27	0.00	-0.57
AFG	0.91	0.91	0.00	0.04	1.01	1.01	-0.01	-0.56
NPL	0.71	0.71	0.00	0.05	1.45	1.44	-0.01	-0.56
BGD	0.98	0.98	0.00	0.02	1.27	1.26	-0.01	-0.55
ZMB	0.24	0.24	0.00	-0.03	0.24	0.24	0.00	-0.55
CAF	0.25	0.25	0.00	-0.04	0.23	0.23	0.00	-0.49
TGO	0.09	0.09	0.00	-0.02	0.10	0.10	0.00	-0.49
MLI	0.60	0.60	0.00	-0.01	0.41	0.40	0.00	-0.46
SDN	1.83	1.83	0.00	0.00	1.52	1.52	-0.01	-0.46
GMB	0.02	0.02	0.00	-0.02	0.02	0.02	0.00	-0.45
UGA	0.76	0.76	0.00	-0.03	0.78	0.78	0.00	-0.42
TZA	0.97	0.97	0.00	-0.02	1.03	1.03	0.00	-0.42
LBR	0.05	0.05	0.00	-0.03	0.05	0.05	0.00	-0.39
TCD	0.34	0.34	0.00	-0.01	0.21	0.21	0.00	-0.37
PNG	0.34	0.34	0.00	0.13	0.47	0.47	0.00	-0.35
GIN	0.26	0.26	0.00	-0.01	0.24	0.24	0.00	-0.35
KEN	1.24	1.24	0.00	-0.02	1.00	0.99	0.00	-0.34
SLB	0.02	0.02	0.00	0.12	0.03	0.03	0.00	-0.33
MDG	0.59	0.59	0.00	-0.02	0.52	0.52	0.00	-0.32
SYR	0.69	0.69	0.00	0.04	0.65	0.65	0.00	-0.30
NER	0.99	0.99	0.00	-0.01	0.66	0.66	0.00	-0.27
MWI	0.20	0.20	0.00	-0.02	0.21	0.21	0.00	-0.25
COD	0.25	0.25	0.00	-0.01	0.16	0.16	0.00	-0.21
MOZ	0.53	0.53	0.00	-0.02	0.51	0.51	0.00	-0.21
BDI	0.10	0.10	0.00	-0.01	0.12	0.12	0.00	-0.18
ERI	0.07	0.07	0.00	-0.01	0.07	0.07	0.00	-0.15
ETH	1.06	1.06	0.00	-0.01	0.79	0.79	0.00	-0.11
MMR	3.37	3.37	0.00	0.00	8.35	8.35	0.00	-0.04

Table A14. Health costs attributable to red and processed meat consumption in the reference (REF) and tax (TAX) scenarios in the year 2020 by region and country (abs: absolute values in USD billion, chg: changes between scenarios in USD billion, pct: percentage change between scenarios). Countries are sorted by greatest percentage changes for processed meat in the TAX scenario.

Region	Red meat				Processed meat			
	REF	TAX			REF	TAX		
	abs	abs	chg	pct	abs	abs	chg	pct
Global	80.74	80.58	-0.16	-0.20	216.53	173.42	-43.10	-19.91
HIC	44.88	44.47	-0.41	-0.91	163.34	127.97	-35.37	-21.66
UMC	10.00	10.06	0.06	0.63	33.76	27.65	-6.11	-18.09
LMC	25.17	25.35	0.18	0.73	18.45	16.86	-1.59	-8.63
LIC	0.41	0.41	0.00	0.06	0.76	0.74	-0.02	-2.25
AFR_LMIC	0.56	0.56	0.00	0.21	0.45	0.43	-0.02	-4.35
AMR_LMIC	5.04	5.00	-0.04	-0.81	13.09	11.59	-1.50	-11.48
EMR_LMIC	0.40	0.40	0.00	0.58	0.56	0.54	-0.02	-2.72
EUR_LMIC	4.86	4.97	0.11	2.21	23.78	18.92	-4.85	-20.41
SEA_LMIC	0.45	0.45	0.00	0.70	3.14	3.01	-0.13	-4.05
WPR_LMIC	24.27	24.44	0.17	0.71	11.95	10.76	-1.20	-10.03
SWE	0.54	0.53	-0.01	-1.49	0.90	0.59	-0.31	-34.74
DEU	3.75	3.63	-0.12	-3.10	12.56	8.43	-4.13	-32.87
NOR	0.30	0.29	-0.01	-3.40	0.65	0.44	-0.21	-32.15
CHP	0.42	0.41	-0.01	-2.77	1.00	0.68	-0.31	-31.31
AUT	0.55	0.53	-0.02	-3.75	2.25	1.56	-0.69	-30.71
SVK	0.10	0.10	0.00	2.27	0.67	0.47	-0.20	-30.20
FNP	0.11	0.11	0.00	0.87	0.77	0.54	-0.23	-29.81
CZE	0.25	0.26	0.00	1.81	1.37	0.99	-0.38	-27.81
NLD	0.58	0.56	-0.02	-4.17	1.59	1.18	-0.41	-25.89
BLT	0.10	0.11	0.00	2.56	0.59	0.44	-0.15	-25.71
RUS	3.55	3.63	0.08	2.24	15.20	11.34	-3.85	-25.35
DNK	0.36	0.34	-0.02	-4.45	0.92	0.69	-0.23	-24.88
BLX	0.36	0.35	-0.01	-1.93	1.03	0.78	-0.25	-24.37
ITP	2.30	2.26	-0.04	-1.82	5.08	3.87	-1.21	-23.87
HUN	0.14	0.15	0.00	1.01	1.22	0.94	-0.28	-22.82
PRT	0.38	0.37	-0.01	-2.52	0.38	0.29	-0.08	-22.30
GRC	0.40	0.40	0.01	1.49	0.18	0.14	-0.04	-22.15
POL	0.74	0.74	0.00	-0.36	4.24	3.34	-0.89	-21.07
HRV	0.07	0.07	0.00	0.90	0.21	0.17	-0.04	-20.54
CAN	1.60	1.61	0.02	0.99	3.02	2.40	-0.62	-20.49
USA	20.78	20.63	-0.15	-0.74	105.03	83.73	-21.31	-20.28
SPP	1.57	1.55	-0.02	-1.28	2.64	2.11	-0.53	-20.11
UKP	1.28	1.28	-0.01	-0.41	5.14	4.14	-1.00	-19.38
IRL	0.11	0.11	0.00	0.19	0.48	0.39	-0.09	-19.34
JPN	2.89	2.89	0.00	-0.05	2.24	1.82	-0.42	-18.60
CUB	0.04	0.04	0.00	0.27	0.08	0.07	-0.02	-18.09
KAZ	0.26	0.27	0.00	1.62	1.38	1.15	-0.24	-17.07
AUS	1.53	1.55	0.02	1.50	2.06	1.71	-0.34	-16.72
FRP	1.73	1.68	-0.04	-2.58	5.68	4.73	-0.95	-16.70
CRB	0.02	0.03	0.00	1.06	0.07	0.06	-0.01	-15.09
SVN	0.06	0.06	0.00	-0.37	0.12	0.10	-0.02	-14.77

BGR	0.13	0.13	0.00	2.98	0.51	0.44	-0.07	-13.74
NZL	0.13	0.13	0.00	0.18	0.42	0.36	-0.06	-13.62
BRA	3.00	2.98	-0.03	-0.87	4.96	4.32	-0.63	-12.79
MEX	0.99	0.97	-0.02	-1.68	5.57	4.86	-0.71	-12.79
ISL	0.01	0.01	0.00	0.49	0.02	0.02	0.00	-12.47
OBN	0.06	0.06	0.00	0.45	0.15	0.13	-0.02	-11.78
ISR	0.09	0.10	0.00	0.71	0.12	0.11	-0.01	-11.51
OSA	0.16	0.17	0.01	3.99	0.42	0.37	-0.05	-11.39
CHM	23.75	23.92	0.17	0.72	10.33	9.19	-1.14	-11.06
BLR	0.11	0.11	0.00	3.58	1.35	1.21	-0.14	-10.69
ROU	0.30	0.30	0.01	2.11	0.94	0.84	-0.10	-10.20
GNQ	0.00	0.00	0.00	-0.02	0.00	0.00	0.00	-9.62
ALB	0.02	0.02	0.00	0.56	0.05	0.05	0.00	-9.31
GEO	0.02	0.02	0.00	1.21	0.20	0.18	-0.02	-8.93
GSA	0.00	0.00	0.00	-0.14	0.00	0.00	0.00	-8.75
URY	0.05	0.05	0.00	0.22	0.08	0.07	-0.01	-8.60
UKR	0.18	0.18	0.00	2.77	2.57	2.36	-0.22	-8.36
PAN	0.02	0.02	0.00	-0.59	0.13	0.12	-0.01	-8.31
CHL	0.22	0.22	0.00	0.12	0.31	0.29	-0.03	-8.14
MDA	0.01	0.01	0.00	2.15	0.09	0.08	-0.01	-7.86
TKM	0.04	0.04	0.00	1.53	0.22	0.20	-0.02	-7.66
SAU	0.11	0.11	0.00	1.53	0.07	0.06	0.00	-7.45
DOM	0.02	0.02	0.00	0.29	0.06	0.05	0.00	-7.33
LBY	0.03	0.03	0.00	1.36	0.01	0.01	0.00	-7.16
CRI	0.01	0.01	0.00	-0.43	0.12	0.11	-0.01	-7.14
PRY	0.04	0.04	0.00	-0.45	0.08	0.07	-0.01	-6.86
KOR	1.30	1.32	0.02	1.52	0.68	0.63	-0.05	-6.72
TUR	0.20	0.20	0.00	0.87	0.18	0.17	-0.01	-6.51
THA	0.18	0.18	0.00	1.44	0.60	0.56	-0.04	-6.46
RAP	0.15	0.15	0.00	0.79	0.08	0.07	0.00	-6.27
ZAF	0.32	0.32	0.00	0.15	0.27	0.26	-0.02	-6.24
ARM	0.01	0.01	0.00	1.53	0.08	0.08	-0.01	-6.15
MYS	0.06	0.07	0.00	1.89	0.56	0.53	-0.03	-6.09
GAB	0.00	0.00	0.00	0.01	0.00	0.00	0.00	-5.96
LKA	0.00	0.00	0.00	0.77	0.04	0.04	0.00	-5.38
VEN	0.08	0.08	0.00	0.53	0.50	0.47	-0.03	-5.35
AZE	0.02	0.02	0.00	1.19	0.19	0.18	-0.01	-5.33
CYP	0.02	0.02	0.00	2.57	0.02	0.02	0.00	-5.31
ECU	0.04	0.04	0.00	-0.10	0.13	0.12	-0.01	-5.22
PER	0.02	0.02	0.00	0.10	0.07	0.06	0.00	-5.10
COL	0.08	0.08	0.00	0.25	0.41	0.39	-0.02	-5.07
EGY	0.12	0.12	0.00	0.46	0.11	0.11	-0.01	-4.97
SLV	0.00	0.00	0.00	0.33	0.03	0.03	0.00	-4.78
OIO	0.00	0.00	0.00	0.44	0.01	0.01	0.00	-4.53
UZB	0.04	0.04	0.00	0.64	0.22	0.21	-0.01	-4.33
MNG	0.04	0.04	0.00	0.33	0.10	0.10	0.00	-4.01
ARG	0.39	0.39	0.00	0.25	0.42	0.40	-0.02	-3.97
LBN	0.02	0.02	0.00	1.69	0.03	0.03	0.00	-3.83
IND	0.06	0.06	0.00	0.04	1.91	1.83	-0.07	-3.76

BLZ	0.00	0.00	0.00	-0.08	0.00	0.00	0.00	-3.73
BWA	0.00	0.00	0.00	-0.22	0.01	0.01	0.00	-3.64
IRQ	0.01	0.01	0.00	0.57	0.01	0.01	0.00	-3.51
DZA	0.11	0.11	0.00	0.69	0.02	0.02	0.00	-3.15
NAM	0.00	0.00	0.00	-0.26	0.01	0.01	0.00	-3.04
FJI	0.00	0.00	0.00	0.71	0.01	0.01	0.00	-3.03
MOR	0.08	0.08	0.00	0.48	0.04	0.04	0.00	-2.87
BTN	0.00	0.00	0.00	0.32	0.00	0.00	0.00	-2.79
JAM	0.00	0.00	0.00	0.15	0.01	0.01	0.00	-2.71
IRN	0.12	0.12	0.00	0.66	0.17	0.17	0.00	-2.68
BOL	0.02	0.02	0.00	0.05	0.05	0.05	0.00	-2.67
TUN	0.03	0.03	0.00	0.56	0.02	0.02	0.00	-2.61
IDN	0.19	0.19	0.00	0.26	0.56	0.55	-0.01	-2.54
COG	0.00	0.00	0.00	-0.03	0.00	0.00	0.00	-2.52
GTM	0.00	0.00	0.00	0.03	0.03	0.03	0.00	-2.40
NIC	0.00	0.00	0.00	0.10	0.01	0.01	0.00	-2.31
JOR	0.01	0.01	0.00	0.60	0.00	0.00	0.00	-2.12
PHL	0.13	0.13	0.00	0.45	0.54	0.53	-0.01	-2.12
HND	0.00	0.00	0.00	0.08	0.02	0.02	0.00	-1.90
KGZ	0.01	0.01	0.00	0.30	0.03	0.03	0.00	-1.61
VNM	0.27	0.27	0.00	-0.02	0.38	0.38	-0.01	-1.60
CIV	0.00	0.00	0.00	-0.06	0.00	0.00	0.00	-1.53
DJI	0.00	0.00	0.00	-0.11	0.00	0.00	0.00	-1.46
AGO	0.02	0.02	0.00	0.00	0.02	0.02	0.00	-1.44
SWZ	0.00	0.00	0.00	-0.07	0.00	0.00	0.00	-1.21
GHA	0.01	0.01	0.00	-0.05	0.01	0.00	0.00	-1.18
LSO	0.00	0.00	0.00	-0.11	0.00	0.00	0.00	-1.12
SLE	0.00	0.00	0.00	-0.07	0.00	0.00	0.00	-1.09
HTI	0.00	0.00	0.00	-0.06	0.00	0.00	0.00	-1.06
PAK	0.03	0.03	0.00	0.04	0.16	0.16	0.00	-1.01
LAO	0.00	0.00	0.00	0.22	0.01	0.01	0.00	-0.99
TJK	0.00	0.00	0.00	0.15	0.01	0.01	0.00	-0.90
CMR	0.00	0.00	0.00	-0.02	0.01	0.01	0.00	-0.89
NGA	0.04	0.04	0.00	-0.02	0.05	0.05	0.00	-0.84
GNB	0.00	0.00	0.00	-0.03	0.00	0.00	0.00	-0.78
KHM	0.01	0.01	0.00	0.16	0.01	0.01	0.00	-0.73
TLS	0.00	0.00	0.00	0.10	0.00	0.00	0.00	-0.68
BFA	0.00	0.00	0.00	-0.03	0.00	0.00	0.00	-0.64
MRT	0.00	0.00	0.00	-0.01	0.00	0.00	0.00	-0.61
BEN	0.00	0.00	0.00	-0.01	0.00	0.00	0.00	-0.61
YEM	0.00	0.00	0.00	0.09	0.00	0.00	0.00	-0.59
SEN	0.00	0.00	0.00	-0.03	0.00	0.00	0.00	-0.57
AFG	0.01	0.01	0.00	0.04	0.01	0.01	0.00	-0.56
NPL	0.00	0.00	0.00	0.05	0.01	0.01	0.00	-0.56
BGD	0.01	0.01	0.00	0.02	0.01	0.01	0.00	-0.55
ZMB	0.00	0.00	0.00	-0.03	0.00	0.00	0.00	-0.55
CAF	0.00	0.00	0.00	-0.04	0.00	0.00	0.00	-0.50
TGO	0.00	0.00	0.00	-0.02	0.00	0.00	0.00	-0.49
MLI	0.00	0.00	0.00	-0.01	0.00	0.00	0.00	-0.46

SDN	0.02	0.02	0.00	0.00	0.02	0.02	0.00	-0.46
GMB	0.00	0.00	0.00	-0.02	0.00	0.00	0.00	-0.45
UGA	0.01	0.01	0.00	-0.03	0.01	0.01	0.00	-0.42
TZA	0.01	0.01	0.00	-0.02	0.01	0.01	0.00	-0.42
LBR	0.00	0.00	0.00	-0.03	0.00	0.00	0.00	-0.39
TCD	0.00	0.00	0.00	-0.01	0.00	0.00	0.00	-0.37
PNG	0.00	0.00	0.00	0.13	0.00	0.00	0.00	-0.35
GIN	0.00	0.00	0.00	-0.01	0.00	0.00	0.00	-0.35
KEN	0.01	0.01	0.00	-0.02	0.01	0.01	0.00	-0.34
SLB	0.00	0.00	0.00	0.12	0.00	0.00	0.00	-0.33
MDG	0.00	0.00	0.00	-0.02	0.00	0.00	0.00	-0.32
SYR	0.00	0.00	0.00	0.04	0.00	0.00	0.00	-0.30
NER	0.00	0.00	0.00	-0.01	0.00	0.00	0.00	-0.27
MWI	0.00	0.00	0.00	-0.02	0.00	0.00	0.00	-0.25
COD	0.00	0.00	0.00	-0.01	0.00	0.00	0.00	-0.21
MOZ	0.00	0.00	0.00	-0.02	0.00	0.00	0.00	-0.21
BDI	0.00	0.00	0.00	-0.01	0.00	0.00	0.00	-0.18
ERI	0.00	0.00	0.00	-0.01	0.00	0.00	0.00	-0.15
ETH	0.00	0.00	0.00	-0.01	0.00	0.00	0.00	-0.11
MMR	0.00	0.00	0.00	0.00	0.01	0.01	0.00	-0.04

Table A15. Tax revenues from red and processed meat (in USD billion) in the year 2020 by region and country. Countries are sorted by greatest tax revenues for processed meat.

Region	Red meat	Processed meat
Global	69.67	102.32
HIC	38.19	71.25
UMC	8.89	18.16
LMC	21.95	12.22
LIC	0.37	0.54
AFR_LMIC	0.50	0.32
AMR_LMIC	4.33	7.27
EMR_LMIC	0.37	0.39
EUR_LMIC	4.49	12.77
SEA_LMIC	0.42	2.21
WPR_LMIC	21.12	7.95
USA	17.67	43.77
RUS	3.29	7.77
CHM	20.65	6.86
DEU	3.11	5.00
FRP	1.46	2.84
MEX	0.86	2.83
BRA	2.55	2.83
UKP	1.13	2.64
ITP	1.93	2.44
POL	0.66	2.02
UKR	0.17	1.57
CAN	1.37	1.52

SPP	1.30	1.36
JPN	2.57	1.34
IND	0.06	1.34
AUS	1.27	1.09
AUT	0.44	0.80
NLD	0.49	0.75
KAZ	0.24	0.75
BLR	0.10	0.73
CZE	0.23	0.61
ROU	0.27	0.58
HUN	0.13	0.57
BLX	0.31	0.49
KOR	1.12	0.46
CHP	0.35	0.43
IDN	0.17	0.42
THA	0.17	0.40
DNK	0.29	0.40
SWE	0.45	0.39
MYS	0.06	0.37
PHL	0.12	0.36
FNP	0.10	0.33
VEN	0.08	0.31
BGR	0.12	0.30
SVK	0.09	0.30
NOR	0.25	0.29
BLT	0.10	0.28
VNM	0.24	0.28
ARG	0.33	0.27
COL	0.07	0.26
OSA	0.15	0.25
IRL	0.09	0.23
NZL	0.11	0.22
PRT	0.32	0.21
CHL	0.19	0.20
ZAF	0.29	0.19
UZB	0.04	0.15
TKM	0.03	0.13
AZE	0.02	0.13
TUR	0.18	0.12
IRN	0.11	0.12
GEO	0.02	0.12
PAK	0.03	0.12
HRV	0.06	0.11
GRC	0.35	0.10
OBN	0.06	0.09
ECU	0.04	0.08
EGY	0.11	0.08
ISR	0.08	0.08
PAN	0.02	0.07

SVN	0.05	0.07
CRI	0.01	0.07
MNG	0.03	0.06
MDA	0.01	0.05
ARM	0.01	0.05
CUB	0.03	0.05
RAP	0.13	0.05
PRY	0.03	0.05
URY	0.05	0.05
PER	0.02	0.05
SAU	0.10	0.04
DOM	0.02	0.04
NGA	0.03	0.04
CRB	0.02	0.04
BOL	0.02	0.04
ALB	0.02	0.03
LKA	0.00	0.03
MOR	0.08	0.03
LBN	0.02	0.02
GTM	0.00	0.02
SLV	0.00	0.02
KGZ	0.01	0.02
HND	0.00	0.02
SDN	0.02	0.02
AGO	0.01	0.01
DZA	0.10	0.01
CYP	0.02	0.01
ISL	0.01	0.01
TUN	0.03	0.01
JAM	0.00	0.01
LAO	0.00	0.01
LBY	0.02	0.01
BGD	0.01	0.01
NPL	0.00	0.01
NIC	0.00	0.01
CMR	0.00	0.01
IRQ	0.01	0.01
KHM	0.01	0.01
TJK	0.00	0.01
KEN	0.01	0.01
TZA	0.01	0.01
AFG	0.00	0.00
MMR	0.00	0.00
FJI	0.00	0.00
OIO	0.00	0.00
NAM	0.00	0.00
BWA	0.00	0.00
UGA	0.00	0.00
GHA	0.00	0.00

JOR	0.01	0.00
BFA	0.00	0.00
HTI	0.00	0.00
PNG	0.00	0.00
ETH	0.00	0.00
GAB	0.00	0.00
ZMB	0.00	0.00
BTN	0.00	0.00
SYR	0.00	0.00
MDG	0.00	0.00
NER	0.00	0.00
MLI	0.00	0.00
CIV	0.00	0.00
GSA	0.00	0.00
MOZ	0.00	0.00
SEN	0.00	0.00
YEM	0.00	0.00
GIN	0.00	0.00
COG	0.00	0.00
MRT	0.00	0.00
BEN	0.00	0.00
SWZ	0.00	0.00
TCD	0.00	0.00
LSO	0.00	0.00
BLZ	0.00	0.00
MWI	0.00	0.00
CAF	0.00	0.00
TLS	0.00	0.00
DJI	0.00	0.00
TGO	0.00	0.00
SLE	0.00	0.00
COD	0.00	0.00
BDI	0.00	0.00
GNB	0.00	0.00
SLB	0.00	0.00
LBR	0.00	0.00
ERI	0.00	0.00
GMB	0.00	0.00
GNQ	0.00	0.00

Table A16 Impacts of cost-compensating taxation of red and processed meat globally and by regions in different income categories. Country-level results and uncertainty intervals are listed in the Supplementary Data File.

Item	Red meat					Processed meat				
	Global	High-income countries	Upper middle-income countries	Lower middle-income countries	Low-income countries	Global	High-income countries	Upper middle-income countries	Lower middle-income countries	Low-income countries
Optimal tax (USD/kg)	0.46	1.15	0.43	0.24	0.03	3.09	7.55	3.41	1.54	0.16
Price before tax (USD/kg)	6.75	4.42	6.05	6.93	8.75	5.74	3.75	5.14	5.89	7.44
Price after tax (USD/kg)	7.21	5.57	6.48	7.17	8.78	8.83	11.30	8.55	7.43	7.60
Price change (%)	6.81	26.02	7.11	3.46	0.34	53.83	201.33	66.34	26.15	2.15
Consumption before tax (g/d)	56.65	94.91	65.97	53.48	25.70	16.52	48.14	25.99	8.88	6.77
Consumption after tax (g/d)	57.19	95.72	66.56	54.09	25.72	12.97	31.71	20.90	8.15	6.66
Consumption change (g/d)	0.54	0.81	0.59	0.61	0.02	-3.55	-16.43	-5.09	-0.73	-0.11
Consumption change (%)	0.95	0.85	0.89	1.14	0.08	-21.49	-34.13	-19.58	-8.22	-1.62
Attributable deaths before tax (thousands)	863.06	167.22	124.08	531.38	34.90	1,533.21	604.53	384.96	484.43	55.69
Attributable deaths after tax (thousands)	872.02	168.25	125.96	537.34	34.95	1,217.50	419.84	300.26	439.56	54.48
Change in attributable deaths (thousands)	8.96	1.03	1.88	5.96	0.05	-315.71	-184.69	-84.70	-44.87	-1.21
Change in attributable deaths (%)	1.04	0.62	1.52	1.12	0.14	-20.59	-30.55	-22.00	-9.26	-2.17
Health care-related costs before tax (USD billion)	80.74	44.88	10.00	25.17	0.41	216.53	163.34	33.76	18.45	0.76
Health care-related costs after tax (USD billion)	81.60	45.28	10.16	25.46	0.42	157.04	113.90	25.79	16.42	0.73
Change in health care-related costs (USD billion)	0.86	0.40	0.16	0.29	0.01	-59.49	-49.44	-7.97	-2.03	-0.03
Change in health care-related costs (%)	1.07	0.89	1.60	1.15	2.44	-27.47	-30.27	-23.61	-11.00	-3.95

Abbreviations: HIC: high-income countries, UMC: upper middle-income countries, LMC: lower middle-income countries, LIC: low-income countries

Table A17. Changes in energy intake (kcal/d) by food group and region. Countries are sorted by greatest reduction in energy intake.

Region	Total	Red meat	Processed meat	Poultry	Milk	Eggs	Oils
Global	-2.27	0.46	-5.03	2.54	0.59	0.14	-0.98
HIC	-13.73	-1.64	-21.11	9.97	2.50	0.39	-3.85
UMC	-4.08	0.13	-7.42	3.34	0.80	0.15	-1.08
LMC	0.59	1.23	-1.57	1.08	0.19	0.11	-0.45
LIC	-0.18	0.02	-0.22	0.03	0.01	0.00	-0.02
AFR_LMIC	-0.01	0.02	-0.14	0.15	0.03	0.00	-0.07
AMR_LMIC	-4.92	-0.90	-6.47	2.65	0.43	0.08	-0.71
EMR_LMIC	0.21	0.16	-0.15	0.25	0.07	0.01	-0.13
EUR_LMIC	-2.89	2.26	-9.02	3.65	1.51	0.27	-1.57
SEA_LMIC	-0.34	0.07	-0.50	0.17	0.06	0.01	-0.14
WPR_LMIC	1.87	2.87	-3.11	2.40	0.34	0.25	-0.88
AUT	-52.22	-9.55	-48.44	7.25	3.19	0.50	-5.17
FNP	-44.19	1.26	-53.19	5.90	4.05	0.27	-2.48
CHP	-39.62	-9.77	-35.16	5.20	4.18	0.37	-4.44
DNK	-34.55	-10.51	-32.58	6.15	3.08	0.68	-1.37
NOR	-34.36	-9.78	-29.84	4.98	3.94	0.42	-4.06
DEU	-33.74	-6.35	-32.78	5.72	3.68	0.45	-4.47
FRP	-27.17	-5.60	-26.77	5.16	2.27	0.30	-2.53
POL	-25.15	-0.73	-30.04	5.88	1.68	0.31	-2.26
NLD	-23.67	-6.42	-24.34	6.14	4.09	0.56	-3.69
ITP	-22.94	-4.06	-21.32	3.69	2.64	0.31	-4.19
UKP	-21.47	-1.50	-26.39	6.59	2.12	0.22	-2.51
IRL	-17.40	0.43	-24.28	5.93	2.45	0.15	-2.07
BLX	-16.80	-3.18	-19.24	6.12	2.92	0.35	-3.77
SPP	-16.24	-3.47	-15.96	4.97	1.46	0.31	-3.54
HUN	-15.35	1.14	-25.66	9.82	1.81	0.44	-2.88
SWE	-14.45	-3.95	-17.77	5.78	5.43	0.45	-4.38
USA	-13.84	-1.48	-30.66	20.05	3.76	0.53	-6.04
SVK	-13.31	2.73	-23.24	8.01	1.55	0.47	-2.82
CZE	-13.01	2.97	-24.58	9.70	2.55	0.30	-3.94
MEX	-12.60	-1.75	-14.40	3.59	0.54	0.20	-0.79
ISL	-12.37	0.88	-17.37	2.63	2.09	0.11	-0.71
PRT	-11.80	-5.72	-10.96	5.41	1.94	0.23	-2.69
SVN	-9.81	-1.03	-12.94	3.89	1.74	0.12	-1.59
NZL	-9.45	0.01	-16.66	7.42	0.64	0.18	-1.04
KAZ	-8.15	3.16	-14.58	2.70	1.64	0.11	-1.18
HRV	-7.41	1.37	-10.97	2.19	1.81	0.24	-2.06
BLT	-6.15	3.84	-17.32	6.30	2.75	0.35	-2.07
BRA	-5.88	-1.98	-7.65	4.15	0.68	0.07	-1.17
GEO	-5.23	1.05	-7.87	1.27	0.94	0.08	-0.70
BLR	-5.02	5.59	-13.94	3.49	1.21	0.31	-1.68
MNG	-5.00	1.75	-6.76	0.04	0.21	0.01	-0.23
PRY	-4.91	-0.87	-4.22	0.45	0.16	0.09	-0.51
PAN	-4.83	-0.40	-7.51	3.30	0.34	0.05	-0.60

RUS	-4.22	2.84	-13.27	5.99	2.18	0.45	-2.40
CRI	-3.56	-0.21	-4.75	1.49	0.48	0.06	-0.64
URY	-2.98	0.55	-5.35	1.74	0.59	0.11	-0.64
OBN	-2.86	0.32	-4.12	0.91	0.75	0.07	-0.79
ARM	-2.75	1.12	-4.82	0.63	0.63	0.08	-0.39
ECU	-2.19	-0.09	-2.90	1.02	0.18	0.02	-0.41
TKM	-2.13	4.42	-7.29	0.46	0.62	0.07	-0.41
CUB	-1.82	0.17	-3.30	1.46	0.37	0.08	-0.60
COL	-1.73	0.20	-2.67	0.84	0.25	0.04	-0.39
ALB	-1.68	0.62	-4.40	1.15	1.27	0.05	-0.38
AZE	-1.54	0.88	-3.08	0.51	0.28	0.04	-0.17
VNM	-1.47	-0.05	-1.63	0.23	0.01	0.01	-0.04
UKR	-1.29	1.82	-5.42	2.24	1.09	0.21	-1.23
UZB	-1.09	0.75	-1.91	0.07	0.27	0.02	-0.28
PHL	-0.98	0.60	-1.85	0.32	0.02	0.02	-0.09
CHL	-0.95	0.24	-4.74	3.72	0.39	0.06	-0.61
BOL	-0.90	0.06	-1.45	0.50	0.03	0.01	-0.05
MDA	-0.82	0.93	-4.00	2.36	0.94	0.13	-1.19
NAM	-0.79	-0.22	-0.78	0.23	0.06	0.00	-0.09
BWA	-0.77	-0.14	-0.65	0.09	0.10	0.01	-0.18
SLV	-0.76	0.05	-1.49	0.61	0.18	0.04	-0.14
VEN	-0.64	0.28	-2.30	1.75	0.19	0.03	-0.59
BLZ	-0.44	-0.05	-1.28	0.84	0.11	0.01	-0.07
IND	-0.44	0.00	-0.46	0.06	0.07	0.01	-0.11
GTM	-0.41	0.00	-0.65	0.26	0.04	0.02	-0.09
HND	-0.36	0.02	-0.74	0.39	0.07	0.01	-0.10
DOM	-0.35	0.25	-1.98	1.84	0.12	0.03	-0.61
BTN	-0.31	0.16	-0.57	0.03	0.06	0.00	
SWZ	-0.29	-0.08	-0.27	0.05	0.03	0.00	-0.02
IDN	-0.27	0.06	-0.49	0.42	0.02	0.03	-0.30
DJI	-0.26	-0.09	-0.18	0.05	0.02	0.00	-0.07
PER	-0.25	0.01	-0.84	0.63	0.09	0.02	-0.16
AGO	-0.23	0.00	-0.26	0.10	0.01	0.00	-0.08
LSO	-0.20	-0.07	-0.16	0.03	0.01	0.00	-0.01
CAF	-0.17	-0.06	-0.11	0.00	0.00	0.00	-0.01
GNB	-0.17	-0.02	-0.11	0.01	0.00	0.00	-0.04
HTI	-0.17	-0.03	-0.14	0.03	0.01	0.00	-0.04
NIC	-0.17	0.02	-0.44	0.29	0.06	0.01	-0.10
CMR	-0.16	-0.01	-0.14	0.02	0.00	0.00	-0.03
IRQ	-0.16	0.09	-0.04	0.13	0.06	0.01	-0.41
KGZ	-0.16	0.48	-0.81	0.06	0.16	0.01	-0.06
BFA	-0.14	-0.03	-0.11	0.01	0.00	0.00	-0.01
PAK	-0.14	0.01	-0.18	0.02	0.05	0.00	-0.04
NGA	-0.12	-0.01	-0.07	0.01	0.00	0.00	-0.05
CIV	-0.11	-0.01	-0.04	0.02	0.01	0.00	-0.09
LAO	-0.11	0.19	-0.43	0.13	0.00	0.01	-0.01
TJK	-0.11	0.08	-0.16	0.01	0.03	0.00	-0.06
TLS	-0.10	0.11	-0.23	0.06	0.00	0.00	-0.03
COG	-0.08	-0.01	-0.11	0.17	0.02	0.00	-0.15

MRT	-0.08	-0.01	-0.07	0.01	0.03	0.00	-0.04
SLE	-0.08	-0.01	-0.03	0.02	0.00	0.00	-0.07
UGA	-0.08	-0.02	-0.06	0.01	0.00	0.00	-0.01
FJI	-0.07	0.91	-1.54	0.78	0.09	0.02	-0.31
SEN	-0.07	-0.01	-0.05	0.01	0.01	0.00	-0.03
GNQ	-0.06	0.00	-0.14	0.07		0.01	
MOZ	-0.06	-0.01	-0.04	0.00	0.00	0.00	-0.01
SDN	-0.06	0.00	-0.08	0.00	0.04	0.00	-0.02
SLB	-0.06	0.08	-0.08	0.01	0.00	0.00	-0.08
GHA	-0.05	-0.01	-0.05	0.04	0.00	0.00	-0.04
KEN	-0.05	-0.01	-0.04	0.00	0.01	0.00	-0.01
NER	-0.05	-0.01	-0.04	0.00	0.00	0.00	-0.01
TZA	-0.05	-0.01	-0.04	0.00	0.00	0.00	-0.01
ZMB	-0.05	-0.01	-0.05	0.02	0.00	0.00	-0.01
GIN	-0.04	0.00	-0.02	0.00	0.00	0.00	-0.02
GMB	-0.04	0.00	-0.02	0.02	0.00	0.00	-0.04
LBR	-0.04	-0.01	-0.02	0.02	0.00	0.00	-0.03
MLI	-0.04	-0.01	-0.03	0.01	0.01	0.00	-0.01
NPL	-0.04	0.02	-0.06	0.00	0.01	0.00	-0.02
TGO	-0.04	-0.01	-0.03	0.01	0.00	0.00	-0.02
MDG	-0.03	-0.01	-0.03	0.01	0.00	0.00	0.00
TCD	-0.03	0.00	-0.02	0.00	0.00	0.00	-0.01
BEN	-0.02	0.00	-0.04	0.03	0.00	0.00	-0.02
BGD	-0.02	0.00	-0.02	0.01	0.00	0.00	-0.02
MWI	-0.02	-0.01	-0.02	0.00	0.00	0.00	0.00
AFG	-0.01	0.02	-0.05	0.01	0.02	0.00	-0.01
ARG	-0.01	0.93	-2.51	1.68	0.32	0.04	-0.46
BDI	-0.01	0.00	-0.01	0.00	0.00	0.00	0.00
COD	-0.01	0.00	-0.01	0.00	0.00	0.00	
ERI	-0.01	0.00	-0.01	0.00	0.00	0.00	0.00
ETH	-0.01	0.00	-0.01	0.00	0.00	0.00	0.00
MMR	0.00	0.00	-0.02	0.02	0.00	0.00	-0.01
SYR	0.04	0.04	-0.02	0.03	0.02	0.00	-0.03
YEM	0.05	0.03	-0.01	0.05	0.01	0.00	-0.03
OIO	0.06	0.09	-0.56	0.64	0.17	0.02	-0.30
PNG	0.08	0.25	-0.18	0.02	0.00	0.00	
KHM	0.09	0.19	-0.13	0.05	0.00	0.00	-0.02
THA	0.10	1.73	-3.29	1.94	0.11	0.13	-0.53
JPN	0.11	-0.04	-3.15	4.17	0.91	0.54	-2.32
LKA	0.13	0.06	-0.30	0.37	0.09	0.02	-0.11
GAB	0.17	0.01	-0.68	0.92	0.08	0.01	-0.18
EGY	0.44	0.24	-0.22	0.46	0.07	0.01	-0.13
TUN	0.54	0.43	-0.12	0.46	0.15	0.03	-0.41
ZAF	0.55	0.29	-1.60	2.18	0.20	0.04	-0.57
MOR	0.61	0.30	-0.15	0.72	0.08	0.03	-0.37
TUR	0.65	0.38	-0.33	1.04	0.39	0.08	-0.91
DZA	0.67	0.41	-0.06	0.43	0.28	0.02	-0.41
IRN	0.76	0.43	-0.21	0.63	0.10	0.03	-0.22
JAM	0.87	0.06	-0.73	1.66	0.16	0.01	-0.29

GSA	0.90	-0.07	-0.79	1.83	0.22	0.01	-0.30
JOR	1.14	0.41	-0.08	1.07	0.17	0.02	-0.45
MYS	1.22	1.13	-3.89	4.83	0.32	0.16	-1.33
ROU	1.55	2.78	-5.86	3.80	2.20	0.27	-1.64
BGR	1.95	2.59	-7.07	7.20	1.86	0.36	-3.00
CAN	2.04	1.83	-10.91	12.82	2.20	0.33	-4.23
CHM	2.39	3.35	-3.35	2.71	0.40	0.29	-1.01
LBY	2.48	0.95	-0.27	1.73	0.44	0.12	-0.48
KOR	3.41	3.58	-1.71	2.81	0.17	0.20	-1.63
GRC	3.45	3.32	-2.92	3.54	2.69	0.22	-3.40
SAU	3.94	0.86	-0.33	3.89	0.40	0.05	-0.94
LBN	4.22	2.94	-0.77	2.41	0.40	0.07	-0.83
CRB	4.67	1.09	-6.73	10.47	1.04	0.09	-1.29
AUS	4.89	3.98	-9.96	12.51	2.19	0.14	-3.98
RAP	5.33	0.97	-0.59	4.82	0.49	0.10	-0.47
ISR	7.29	0.87	-3.03	10.89	1.05	0.15	-2.63
CYP	7.70	5.54	-1.85	3.96	1.13	0.14	-1.22
OSA	13.34	7.41	-6.76	13.06	1.23	0.45	-2.05

Table A18. Number of avoided deaths (in thousands) associated with tax-related changes in weight levels (via changes in caloric intake, see Appendix A1) (mean: mean, std: standard deviation). Weight levels include underweight (UND), overweight (OVW), obesity (OBS); the combined impact is denoted by WGH. Countries are sorted by greatest number of avoided deaths (WGH, mean).

Region	WGH		UND		OVW		OBS	
	mean	std	mean	std	mean	std	mean	std
Global	3.827	0.223	-1.283	0.128	0.132	0.030	4.978	0.226
HIC	2.944	0.160	-0.848	0.094	0.167	0.017	3.626	0.185
UMC	1.353	0.115	-0.195	0.022	0.045	0.005	1.503	0.116
LMC	-0.453	0.104	-0.174	0.084	-0.088	0.025	-0.192	0.058
LIC	-0.009	0.011	-0.068	0.011	0.008	0.001	0.051	0.003
AFR_LMIC	-0.025	0.002	-0.017	0.002	0.000	0.000	-0.008	0.002
AMR_LMIC	0.981	0.106	-0.181	0.021	0.020	0.003	1.141	0.109
EMR_LMIC	-0.024	0.003	-0.005	0.002	0.001	0.001	-0.020	0.002
EUR_LMIC	0.548	0.046	-0.040	0.008	0.036	0.004	0.552	0.040
SEA_LMIC	-0.124	0.061	-0.336	0.064	0.035	0.007	0.177	0.015
WPR_LMIC	-0.465	0.085	0.142	0.055	-0.126	0.024	-0.481	0.056
DEU	0.800	0.118	-0.211	0.062	0.048	0.013	0.963	0.135
MEX	0.590	0.100	-0.053	0.011	0.000	0.000	0.642	0.103
UKP	0.354	0.047	-0.074	0.020	0.009	0.002	0.420	0.058
ITP	0.349	0.056	-0.075	0.024	0.025	0.007	0.398	0.061
RUS	0.330	0.045	-0.025	0.008	0.023	0.004	0.333	0.038
SPP	0.285	0.043	-0.065	0.020	0.010	0.003	0.340	0.054
BRA	0.264	0.033	-0.098	0.017	0.014	0.003	0.348	0.036
POL	0.231	0.031	-0.027	0.007	0.017	0.003	0.242	0.028
USA	0.194	0.024	-0.037	0.009	-0.002	0.000	0.232	0.030
FRP	0.191	0.043	-0.184	0.054	0.019	0.006	0.356	0.055
FNP	0.096	0.016	-0.027	0.008	0.008	0.002	0.114	0.016

NLD	0.091	0.015	-0.060	0.016	0.009	0.002	0.142	0.020
HUN	0.088	0.012	-0.012	0.003	0.006	0.001	0.094	0.011
SVK	0.056	0.008	-0.004	0.001	0.004	0.001	0.056	0.007
KAZ	0.055	0.006	-0.004	0.001	0.001	0.000	0.058	0.006
CZE	0.053	0.007	-0.005	0.001	0.002	0.001	0.056	0.007
SWE	0.052	0.008	-0.018	0.005	0.005	0.001	0.065	0.009
CHP	0.049	0.010	-0.034	0.011	0.006	0.002	0.077	0.012
UKR	0.040	0.007	-0.001	0.001	0.004	0.001	0.037	0.005
DNK	0.039	0.007	-0.026	0.006	0.004	0.001	0.061	0.008
BLR	0.039	0.006	-0.002	0.001	0.003	0.001	0.038	0.005
OBN	0.032	0.004	-0.007	0.002	0.002	0.000	0.037	0.004
COL	0.031	0.003	-0.010	0.002	0.002	0.000	0.039	0.004
NOR	0.031	0.004	-0.012	0.003	0.002	0.001	0.041	0.006
AUT	0.029	0.005	-0.009	0.003	0.003	0.001	0.035	0.005
PRT	0.028	0.004	-0.006	0.002	0.001	0.000	0.033	0.005
UZB	0.022	0.003	-0.001	0.000	0.002	0.000	0.021	0.002
HRV	0.021	0.003	-0.004	0.001	0.002	0.001	0.023	0.003
BLT	0.020	0.003	-0.002	0.001	0.002	0.000	0.021	0.003
ECU	0.019	0.002	-0.003	0.001	0.000	0.000	0.022	0.002
PHL	0.017	0.007	-0.026	0.006	0.010	0.002	0.033	0.003
IRL	0.016	0.002	-0.003	0.001	0.000	0.000	0.018	0.002
NZL	0.016	0.002	-0.002	0.001	0.000	0.000	0.018	0.002
PRY	0.016	0.002	-0.004	0.001	0.001	0.000	0.018	0.002
VEN	0.014	0.002	-0.001	0.000	0.000	0.000	0.015	0.002
BLX	0.012	0.002	-0.007	0.002	0.001	0.000	0.018	0.003
IDN	0.011	0.004	-0.015	0.003	0.004	0.001	0.021	0.002
GEO	0.011	0.002	0.000	0.000	0.000	0.000	0.011	0.002
PAN	0.009	0.001	-0.002	0.000	0.000	0.000	0.011	0.001
SVN	0.009	0.001	-0.002	0.000	0.000	0.000	0.010	0.001
CUB	0.008	0.001	-0.002	0.001	0.001	0.000	0.009	0.001
ARM	0.007	0.001	0.000	0.000	0.000	0.000	0.007	0.001
BOL	0.007	0.001	-0.002	0.000	0.000	0.000	0.008	0.001
MNG	0.006	0.001	-0.001	0.000	0.000	0.000	0.007	0.001
AZE	0.006	0.001	0.000	0.000	0.000	0.000	0.006	0.001
CHL	0.005	0.001	-0.001	0.000	0.000	0.000	0.006	0.001
CRI	0.005	0.001	-0.001	0.000	0.000	0.000	0.006	0.001
JPN	0.005	0.004	0.012	0.005	-0.002	0.001	-0.005	0.001
URY	0.004	0.001	-0.001	0.000	0.000	0.000	0.005	0.001
TKM	0.004	0.001	0.000	0.000	0.000	0.000	0.004	0.000
MDA	0.003	0.000	0.000	0.000	0.000	0.000	0.003	0.000
PER	0.003	0.000	-0.001	0.000	0.000	0.000	0.004	0.000
SLV	0.003	0.000	-0.001	0.000	0.000	0.000	0.003	0.000
ALB	0.003	0.000	0.000	0.000	0.000	0.000	0.003	0.000
GTM	0.003	0.000	-0.001	0.000	0.000	0.000	0.003	0.000
PAK	0.002	0.002	-0.009	0.002	0.002	0.000	0.009	0.001
IRQ	0.002	0.000	0.000	0.000	0.000	0.000	0.003	0.000
DOM	0.002	0.000	0.000	0.000	0.000	0.000	0.002	0.000
HND	0.001	0.000	0.000	0.000	0.000	0.000	0.001	0.000
ISL	0.001	0.000	0.000	0.000	0.000	0.000	0.001	0.000

HTI	0.001	0.000	-0.001	0.000	0.000	0.000	0.002	0.000
SDN	0.001	0.000	-0.001	0.000	0.000	0.000	0.002	0.000
NIC	0.001	0.000	0.000	0.000	0.000	0.000	0.001	0.000
KGZ	0.001	0.000	0.000	0.000	0.000	0.000	0.001	0.000
TJK	0.001	0.000	0.000	0.000	0.000	0.000	0.001	0.000
CMR	0.000	0.000	-0.002	0.000	0.000	0.000	0.002	0.000
KHM	0.000	0.000	0.001	0.000	0.000	0.000	0.000	0.000
AGO	0.000	0.000	-0.002	0.000	0.000	0.000	0.002	0.000
GHA	0.000	0.000	0.000	0.000	0.000	0.000	0.000	0.000
NAM	0.000	0.000	-0.001	0.000	0.000	0.000	0.001	0.000
ARG	0.000	0.000	0.000	0.000	0.000	0.000	0.000	0.000
BEN	0.000	0.000	0.000	0.000	0.000	0.000	0.000	0.000
BLZ	0.000	0.000	0.000	0.000	0.000	0.000	0.000	0.000
BTN	0.000	0.000	0.000	0.000	0.000	0.000	0.000	0.000
BWA	0.000	0.000	-0.001	0.000	0.000	0.000	0.001	0.000
COG	0.000	0.000	0.000	0.000	0.000	0.000	0.000	0.000
DJI	0.000	0.000	0.000	0.000	0.000	0.000	0.000	0.000
ERI	0.000	0.000	0.000	0.000	0.000	0.000	0.000	0.000
FJI	0.000	0.000	0.000	0.000	0.000	0.000	0.000	0.000
GAB	0.000	0.000	0.000	0.000	0.000	0.000	0.000	0.000
GIN	0.000	0.000	0.000	0.000	0.000	0.000	0.000	0.000
GMB	0.000	0.000	0.000	0.000	0.000	0.000	0.000	0.000
GNQ	0.000	0.000	0.000	0.000	0.000	0.000	0.000	0.000
LBR	0.000	0.000	0.000	0.000	0.000	0.000	0.000	0.000
LSO	0.000	0.000	0.000	0.000	0.000	0.000	0.000	0.000
MMR	0.000	0.000	0.000	0.000	0.000	0.000	0.000	0.000
MRT	0.000	0.000	0.000	0.000	0.000	0.000	0.000	0.000
SLB	0.000	0.000	0.000	0.000	0.000	0.000	0.000	0.000
SWZ	0.000	0.000	0.000	0.000	0.000	0.000	0.000	0.000
TGO	0.000	0.000	0.000	0.000	0.000	0.000	0.000	0.000
TLS	0.000	0.000	0.000	0.000	0.000	0.000	0.000	0.000
AFG	0.000	0.000	0.000	0.000	0.000	0.000	0.000	0.000
BDI	0.000	0.000	0.000	0.000	0.000	0.000	0.000	0.000
CIV	0.000	0.000	-0.001	0.000	0.000	0.000	0.001	0.000
GNB	0.000	0.000	0.000	0.000	0.000	0.000	0.000	0.000
LAO	0.000	0.000	0.000	0.000	0.000	0.000	0.000	0.000
MLI	0.000	0.000	0.000	0.000	0.000	0.000	0.000	0.000
SYR	0.000	0.000	0.000	0.000	0.000	0.000	0.000	0.000
ZMB	0.000	0.000	0.000	0.000	0.000	0.000	0.000	0.000
COD	0.000	0.000	0.000	0.000	0.000	0.000	0.000	0.000
MDG	0.000	0.000	0.000	0.000	0.000	0.000	0.000	0.000
MWI	0.000	0.000	0.000	0.000	0.000	0.000	0.000	0.000
PNG	0.000	0.000	0.000	0.000	0.000	0.000	0.000	0.000
SEN	0.000	0.000	-0.001	0.000	0.000	0.000	0.000	0.000
SLE	0.000	0.000	-0.001	0.000	0.000	0.000	0.000	0.000
TCD	0.000	0.000	0.000	0.000	0.000	0.000	0.000	0.000
NER	0.000	0.000	0.000	0.000	0.000	0.000	0.000	0.000
OIO	0.000	0.000	0.000	0.000	0.000	0.000	0.000	0.000
BFA	0.000	0.000	-0.001	0.000	0.000	0.000	0.000	0.000

ETH	0.000	0.000	-0.001	0.000	0.000	0.000	0.000	0.000
NPL	0.000	0.000	-0.001	0.000	0.000	0.000	0.000	0.000
YEM	0.000	0.000	0.000	0.000	0.000	0.000	-0.001	0.000
CAF	-0.001	0.000	-0.001	0.000	0.000	0.000	0.000	0.000
KEN	-0.001	0.000	-0.001	0.000	0.000	0.000	0.001	0.000
TZA	-0.001	0.000	-0.001	0.000	0.000	0.000	0.001	0.000
BGD	-0.001	0.000	-0.001	0.000	0.000	0.000	0.000	0.000
GSA	-0.001	0.000	0.000	0.000	0.000	0.000	-0.001	0.000
MOZ	-0.001	0.000	-0.001	0.000	0.000	0.000	0.001	0.000
LKA	-0.001	0.000	0.001	0.000	0.000	0.000	-0.002	0.000
UGA	-0.001	0.000	-0.002	0.000	0.000	0.000	0.001	0.000
TUN	-0.001	0.000	0.000	0.000	0.000	0.000	-0.001	0.000
JOR	-0.002	0.000	0.000	0.000	0.000	0.000	-0.002	0.000
JAM	-0.002	0.000	0.000	0.000	0.000	0.000	-0.002	0.000
THA	-0.002	0.000	0.002	0.000	0.000	0.000	-0.003	0.000
NGA	-0.002	0.001	-0.007	0.001	0.000	0.000	0.005	0.001
ISR	-0.002	0.000	0.001	0.000	0.000	0.000	-0.003	0.000
LBY	-0.003	0.000	0.000	0.000	0.000	0.000	-0.003	0.000
LBN	-0.004	0.001	0.000	0.000	0.000	0.000	-0.004	0.001
MOR	-0.004	0.001	0.001	0.000	0.000	0.000	-0.005	0.001
EGY	-0.005	0.001	0.001	0.000	0.000	0.000	-0.006	0.001
TUR	-0.005	0.001	0.000	0.000	0.000	0.000	-0.005	0.001
ROU	-0.006	0.001	0.002	0.001	-0.001	0.000	-0.008	0.001
ZAF	-0.007	0.001	0.007	0.001	0.000	0.000	-0.014	0.002
CYP	-0.008	0.001	0.001	0.000	0.000	0.000	-0.009	0.001
CRB	-0.009	0.002	0.001	0.000	0.000	0.000	-0.010	0.002
MYS	-0.009	0.001	0.003	0.001	-0.001	0.000	-0.011	0.001
DZA	-0.010	0.001	0.002	0.000	-0.001	0.000	-0.011	0.001
OSA	-0.010	0.003	0.008	0.002	-0.004	0.001	-0.014	0.002
GRC	-0.011	0.002	0.001	0.000	0.000	0.000	-0.012	0.002
RAP	-0.012	0.001	0.001	0.000	0.000	0.000	-0.013	0.001
IRN	-0.015	0.002	0.003	0.001	-0.001	0.000	-0.017	0.002
KOR	-0.017	0.005	0.017	0.005	-0.007	0.002	-0.026	0.004
BGR	-0.017	0.002	0.002	0.001	-0.001	0.000	-0.018	0.002
CAN	-0.017	0.002	0.003	0.001	0.000	0.000	-0.020	0.003
VNM	-0.024	0.011	-0.052	0.011	0.006	0.001	0.022	0.002
SAU	-0.026	0.003	0.003	0.001	0.000	0.000	-0.029	0.003
AUS	-0.040	0.006	0.007	0.002	-0.001	0.000	-0.046	0.006
IND	-0.131	0.061	-0.322	0.064	0.031	0.007	0.160	0.015
CHM	-0.455	0.084	0.217	0.053	-0.141	0.024	-0.530	0.056

Table A20. Food-related greenhouse gas emissions in the reference (REF) and tax (TAX) scenarios in the year 2020 by region and country (abs: absolute values in $MtCO_2$-eq, chg: changes between scenarios in $MtCO_2$-eq, pct: percentage change between scenarios). Countries are sorted by greatest percentage changes between the scenarios.

Region	REF	TAX		
	abs	abs	chg	pct
Global	9,232.99	9,124.17	-108.81	-1.18
HIC	1,885.54	1,823.93	-61.62	-3.27
UMC	2,006.16	1,966.26	-39.90	-1.99
LMC	4,340.90	4,333.92	-6.99	-0.16
LIC	885.95	885.33	-0.61	-0.07
AFR_LMIC	857.92	857.31	-0.61	-0.07
AMR_LMIC	1,676.15	1,637.45	-38.70	-2.31
EMR_LMIC	595.51	595.69	0.18	0.03
EUR_LMIC	353.14	350.49	-2.65	-0.75
SEA_LMIC	1,298.96	1,290.90	-8.06	-0.62
WPR_LMIC	2,451.33	2,453.68	2.34	0.10
AUT	12.89	12.02	-0.87	-6.76
DNK	8.89	8.39	-0.50	-5.62
MEX	267.47	254.13	-13.35	-4.99
DEU	103.44	98.31	-5.13	-4.96
FNP	7.17	6.89	-0.28	-3.86
FRP	98.86	95.20	-3.66	-3.70
CHP	11.42	11.00	-0.41	-3.63
BLX	16.38	15.89	-0.50	-3.03
ITP	93.18	90.36	-2.82	-3.03
IRL	7.59	7.37	-0.22	-2.89
BRA	764.76	744.50	-20.26	-2.65
CRI	10.08	9.84	-0.24	-2.41
HUN	9.06	8.84	-0.22	-2.39
KAZ	29.91	29.34	-0.58	-1.93
CZE	10.90	10.71	-0.19	-1.77
ISL	0.47	0.46	-0.01	-1.69
CAN	68.27	67.19	-1.08	-1.58
HRV	3.92	3.88	-0.05	-1.20
COL	102.00	100.85	-1.15	-1.13
GEO	4.25	4.20	-0.05	-1.13
CHL	47.78	47.31	-0.48	-0.99
ECU	33.73	33.41	-0.32	-0.95
IND	852.68	844.74	-7.94	-0.93
BLT	7.04	6.97	-0.06	-0.90
BLR	9.54	9.47	-0.07	-0.77
BWA	3.33	3.30	-0.02	-0.75
ARM	3.85	3.83	-0.03	-0.74
BTN	1.03	1.03	-0.01	-0.73
CUB	11.58	11.50	-0.08	-0.71
ALB	4.03	4.01	-0.02	-0.61
JPN	136.05	135.22	-0.82	-0.60
LKA	13.64	13.57	-0.08	-0.56

HND	11.59	11.53	-0.06	-0.55
AZE	9.13	9.09	-0.05	-0.51
GTM	15.27	15.20	-0.08	-0.50
BLZ	0.45	0.45	0.00	-0.47
BOL	27.42	27.30	-0.12	-0.45
DOM	15.95	15.88	-0.07	-0.43
CRB	3.04	3.03	-0.01	-0.38
GAB	2.26	2.25	-0.01	-0.33
ARG	215.37	214.72	-0.64	-0.30
MNG	12.28	12.24	-0.04	-0.30
GNQ	0.06	0.06	0.00	-0.25
LSO	1.94	1.94	0.00	-0.23
CMR	20.33	20.30	-0.04	-0.19
AGO	32.49	32.44	-0.05	-0.17
MDA	2.30	2.30	0.00	-0.17
DJI	0.81	0.81	0.00	-0.16
GSA	0.99	0.99	0.00	-0.12
HTI	7.55	7.54	-0.01	-0.12
JAM	3.60	3.59	0.00	-0.12
BFA	23.64	23.62	-0.03	-0.11
COG	2.86	2.85	0.00	-0.10
CAF	10.00	9.99	-0.01	-0.09
CIV	13.01	13.00	-0.01	-0.09
GHA	16.57	16.56	-0.01	-0.08
LAO	8.39	8.39	-0.01	-0.08
GNB	1.46	1.46	0.00	-0.07
IDN	180.28	180.16	-0.12	-0.07
KGZ	8.75	8.75	-0.01	-0.07
BEN	6.61	6.61	0.00	-0.06
KEN	64.19	64.16	-0.03	-0.05
MLI	21.13	21.12	-0.01	-0.05
GMB	1.58	1.58	0.00	-0.04
MDG	23.58	23.57	-0.01	-0.04
BDI	4.18	4.18	0.00	-0.03
BGR	6.06	6.06	0.00	-0.03
GIN	13.44	13.44	0.00	-0.03
AFG	36.27	36.26	-0.01	-0.02
BGD	112.52	112.49	-0.02	-0.02
ERI	3.22	3.22	0.00	-0.02
ETH	66.67	66.66	-0.01	-0.02
COD	8.08	8.08	0.00	-0.01
LBR	2.59	2.59	0.00	-0.01
MMR	52.94	52.94	0.00	0.00
FJI	0.84	0.84	0.00	0.02
KHM	14.05	14.05	0.00	0.03
IRQ	20.92	20.94	0.02	0.09
CHM	2,194.44	2,196.89	2.45	0.11
EGY	83.68	83.77	0.09	0.11
AUS	54.57	54.66	0.09	0.16

MOR	30.46	30.54	0.07	0.24
IRN	112.98	113.38	0.39	0.35
JOR	8.70	8.73	0.03	0.38
ISR	15.88	15.96	0.08	0.49
DZA	56.11	56.41	0.30	0.53
LBY	7.08	7.13	0.05	0.74
GRC	17.62	17.77	0.15	0.84
LBN	7.76	7.83	0.07	0.92
CYP	1.26	1.28	0.02	1.21
KOR	81.88	83.05	1.17	1.43

Table A20. Food-related greenhouse gas emissions in the reference (REF) and tax (TAX) scenarios in the year 2020 by food group (abs: absolute values in $MtCO_2$-eq, chg: changes between scenarios in $MtCO_2$-eq, pct: percentage change between scenarios).

Food group	REF abs	TAX abs	TAX chg	TAX pct
total	9,232.99	9,124.17	-108.81	-1.18
beef	3,874.49	3,765.96	-108.53	-2.80
lamb	472.64	467.69	-4.95	-1.05
pork	713.08	686.73	-26.36	-3.70
poultry	601.28	629.34	28.06	4.67
eggs	264.11	265.20	1.09	0.41
dairy	765.18	768.70	3.53	0.46
oils	444.93	443.27	-1.66	-0.37
maize	45.70	45.70	0.00	0.00
wheat	328.06	328.06	0.00	0.00
rice	717.55	717.55	0.00	0.00
other grains	49.10	49.10	0.00	0.00
sugar	45.57	45.57	0.00	0.00
oil crops	18.96	18.96	0.00	0.00
fruits (temperate)	28.22	28.22	0.00	0.00
fruits (tropical)	108.63	108.63	0.00	0.00
vegetables	693.65	693.65	0.00	0.00
roots	48.33	48.33	0.00	0.00
legumes	13.51	13.51	0.00	0.00

Table A21. Main results by region for the year 2050.

Item	Red meat					Processed meat				
	Global	High-income countries	Upper middle-income countries	Lower middle-income countries	Low-income countries	Global	High-income countries	Upper middle-income countries	Lower middle-income countries	Low-income countries
Optimal tax (USD/kg)	0.71	1.75	1.16	0.59	0.10	3.68	7.71	6.17	3.34	0.48
Price before tax (USD/kg)	7.15	4.62	6.13	7.31	8.81	6.08	3.92	5.21	6.22	7.49
Price after tax (USD/kg)	7.86	6.37	7.30	7.91	8.91	9.76	11.63	11.38	9.56	7.97
Price change (%)	9.98	37.95	18.98	8.11	1.11	60.52	196.37	118.28	53.75	6.40
Consumption before tax (g/d)	60.76	99.61	73.18	55.86	43.33	17.97	50.18	28.97	11.39	9.62
Consumption after tax (g/d)	60.99	97.57	72.37	56.92	43.36	14.17	34.31	21.63	9.89	9.24
Consumption change (g/d)	0.23	-2.04	-0.81	1.06	0.03	-3.80	-15.88	-7.35	-1.50	-0.38
Consumption change (%)	0.38	-2.05	-1.10	1.89	0.06	-21.14	-31.64	-25.36	-13.16	-3.97
Attributable deaths before tax (thousands)	1,474.06	196.23	191.26	954.10	118.10	2,387.15	684.86	557.61	959.54	176.10
Attributable deaths after tax (thousands)	1,488.23	192.65	189.60	973.02	118.24	1,889.44	491.09	410.50	815.70	164.29
Change in attributable deaths (thousands)	14.17	-3.58	-1.66	18.93	0.14	-497.70	-193.76	-147.12	-143.84	-11.81
Change in attributable deaths (%)	0.96	-1.82	-0.87	1.98	0.12	-20.85	-28.29	-26.38	-14.99	-6.71
Health care-related costs before tax (USD billion)	258.03	92.03	35.40	123.97	4.55	531.57	313.02	111.13	98.26	7.81
Health care-related costs after tax (USD billion)	258.14	90.04	34.89	126.53	4.56	396.59	227.20	80.08	81.08	7.05
Change in health care-related costs (USD billion)	0.10	-1.99	-0.52	2.56	0.01	-134.98	-85.82	-31.04	-17.18	-0.76
Change in health care-related costs (%)	0.04	-2.17	-1.47	2.06	0.16	-25.39	-27.42	-27.93	-17.48	-9.74
Tax revenues (USD billion)	222.77	77.06	30.58	109.14	4.07	239.66	124.92	50.37	58.46	5.06

Abbreviations: HIC: high-income countries, UMC: upper middle-income countries, LMC: lower middle-income countries, LIC: low-income countries

Table A22. Main results by region for the year 2010.

Item	Red meat					Processed meat				
	Global	High-income countries	Upper middle-income countries	Lower middle-income countries	Low-income countries	Global	High-income countries	Upper middle-income countries	Lower middle-income countries	Low-income countries
Optimal tax (USD/kg)	0.18	0.65	0.24	0.07	0.01	0.95	2.98	1.62	0.43	0.06
Price before tax (USD/kg)	5.70	3.78	5.29	5.84	7.52	4.84	3.21	4.50	4.96	6.39
Price after tax (USD/kg)	5.88	4.43	5.53	5.91	7.53	5.79	6.20	6.12	5.39	6.45
Price change (%)	3.14	17.31	4.60	1.21	0.16	19.55	92.91	36.08	8.60	0.98
Consumption before tax (g/d)	53.08	94.67	62.88	47.38	22.21	16.09	48.59	24.70	7.69	5.95
Consumption after tax (g/d)	53.13	94.14	63.11	47.56	22.22	13.77	37.48	21.94	7.36	5.90
Consumption change (g/d)	0.05	-0.53	0.23	0.19	0.01	-2.33	-11.11	-2.76	-0.33	-0.05
Consumption change (%)	0.10	-0.56	0.37	0.39	0.03	-14.45	-22.87	-11.18	-4.26	-0.85
Attributable deaths before tax (thousands)	679.68	156.36	109.71	384.88	24.65	1,326.06	562.92	351.83	368.95	39.63
Attributable deaths after tax (thousands)	681.15	155.33	110.68	386.38	24.66	1,141.57	448.26	300.82	350.68	39.18
Change in attributable deaths (thousands)	1.47	-1.03	0.97	1.50	0.01	-184.50	-114.65	-51.00	-18.27	-0.45
Change in attributable deaths (%)	0.22	-0.66	0.89	0.39	0.03	-13.91	-20.37	-14.50	-4.95	-1.14
Health care-related costs before tax (USD billion)	43.93	29.86	5.49	8.28	0.17	135.92	108.35	19.55	7.59	0.32
Health care-related costs after tax (USD billion)	43.85	29.69	5.54	8.32	0.17	111.26	87.22	16.46	7.17	0.32
Change in health care-related costs (USD billion)	-0.09	-0.18	0.06	0.03	0.00	-24.66	-21.13	-3.10	-0.42	0.00
Change in health care-related costs (%)	-0.20	-0.59	1.01	0.40	0.05	-18.14	-19.50	-15.83	-5.58	-1.50
Tax revenues (USD billion)	37.99	25.49	4.92	7.30	0.16	65.60	49.12	11.01	5.16	0.23

Abbreviations: HIC: high-income countries, UMC: upper middle-income countries, LMC: lower middle-income countries, LIC: low-income countries

Table A23. Deaths attributable to red and processed meat consumption (in thousands) under different assumption (mean: mean; low/high: low/high values of 95% confidence interval). '*GBD 2013*' denotes estimates of the Global Burden of Disease project,[63] '*Aggregate risk factors*' denotes estimates using general disease association for cardiovascular disease and cancer, '*Disaggregate risk factors*' denotes estimates using specific health endpoints as used in the main analysis, '*TMREL*' denotes estimates using theoretical minimum exposure levels for red meat (100 g per week) as used by the GBD,[63] '*2000 kcal/d*' denotes estimates for consumption data standardised to an energy intake of 2000 kcal/d.

	Red meat			Processed meat		
	mean	low	high	mean	low	high
GBD 2013						
Coronary heart disease				526	383	719
Colon and rectum cancers	50	40	61	34	24	47
Type-2 diabetes mellitus	52	46	58	84	58	120
All causes	**102**	89	116	**644**	467	881
Aggregate risk factors						
Cardiovascular disease	1710	1230	2214	850	423	1272
Cancer	640	391	868	279	213	373
Type-2 diabetes mellitus	118	28	202	180	105	247
All causes	2468	1649	3285	1309	741	1892
Selected GBD causes	**758**	419	1070	**1309**	741	1892
Difference to GBD 2013 (%)	643	371	823	103	59	115
Disaggregate risk factors						
Coronary heart disease				895	192	1470
Stroke	482	122	786	192	37	328
Colon and rectum cancers	79	26	128	59	35	85
Type-2 diabetes mellitus	118	28	202	180	105	247
All causes	680	176	1116	1326	370	2130
GBD causes	**197**	54	330	**1134**	332	1803
Difference to GBD 2013 (%)	93	-39	185	76	-29	105
TMREL						
Coronary heart disease				895	192	1470
Stroke	378	95	617	192	37	328
Colon and rectum cancers	65	21	106	59	35	85
Type-2 diabetes mellitus	86	21	146	180	105	247
All causes	529	137	870	1326	370	2130
GBD causes	**151**	42	252	**1134**	332	1803
Difference to GBD 2013 (%)	48	-53	118	76	-29	105
2000 kcal/d						
Coronary heart disease				742	155	1247
Stroke	401	100	657	158	31	271
Colon and rectum cancers	63	20	104	47	28	68
Type-2 diabetes mellitus	97	23	167	149	85	207
All causes	560	144	928	1095	298	1793
GBD causes	**160**	43	271	**937**	268	1522
Difference to GBD 2013 (%)	57	-51	134	46	-43	73
TMREL and 2000kcal/d						
Coronary heart disease				742	155	1247
Stroke	295	74	485	158	31	271
Colon and rectum cancers	49	16	81	47	28	68

Type-2 diabetes mellitus	64	15	110	149	85	207
All causes	407	104	675	1095	298	1793
GBD causes	**112**	31	191	**937**	268	1522
Difference to GBD 2013 (%)	10	-65	64	46	-43	73

Table A24. Main results for a sensitivity analysis which includes only direct health care-related instead of total costs (see Table A3) in the calculation of optimal tax levels.

	Red meat					Processed meat				
Item	Global	High-income countries	Upper middle-income countries	Lower middle-income countries	Low-income countries	Global	High-income countries	Upper middle-income countries	Lower middle-income countries	Low-income countries
Optimal tax (USD/kg)	0.17	0.70	0.24	0.06	0.01	0.66	2.50	1.04	0.25	0.03
Price before tax (USD/kg)	6.75	4.42	6.05	6.93	8.75	5.74	3.75	5.14	5.89	7.44
Price after tax (USD/kg)	6.92	5.12	6.29	6.99	8.76	6.40	6.25	6.18	6.14	7.47
Price change (%)	2.52	15.84	3.97	0.87	0.11	11.50	66.67	20.23	4.24	0.40
Consumption before tax (g/d)	56.65	94.91	65.97	53.48	25.70	16.52	48.14	25.99	8.88	6.77
Consumption after tax (g/d)	56.50	93.85	65.76	53.54	25.70	14.81	39.42	23.77	8.69	6.74
Consumption change (g/d)	-0.15	-1.06	-0.21	0.06	0.00	-1.71	-8.72	-2.22	-0.19	-0.03
Consumption change (%)	-0.26	-1.12	-0.32	0.11	0.00	-10.35	-18.11	-8.54	-2.14	-0.44
Attributable deaths before tax (thousands)	863.06	167.22	124.08	531.38	34.90	1,533.21	604.53	384.96	484.43	55.69
Attributable deaths after tax (thousands)	861.80	165.45	123.98	532.00	34.90	1,395.13	511.84	351.74	472.65	55.40
Change in attributable deaths (thousands)	-1.26	-1.77	-0.10	0.62	0.00	-138.08	-92.69	-33.22	-11.78	-0.29
Change in attributable deaths (%)	-0.15	-1.06	-0.08	0.12	0.00	-9.01	-15.33	-8.63	-2.43	-0.52
Health care-related costs before tax (USD billion)	50.49	33.92	6.30	9.92	0.19	126.77	105.49	15.45	5.47	0.26
Health care-related costs after tax (USD billion)	50.06	33.49	6.28	9.94	0.19	108.37	88.69	14.03	5.30	0.26
Change in health care-related costs (USD billion)	-0.43	-0.43	-0.02	0.02	0.00	-18.40	-16.80	-1.42	-0.17	0.00
Change in health care-related costs (%)	-0.85	-1.27	-0.32	0.20	0.00	-14.51	-15.93	-9.19	-3.11	0.00
Tax revenues (USD billion)	42.96	28.59	5.49	8.56	0.17	61.75	48.56	9.07	3.87	0.19

Abbreviations: HIC: high-income countries, UMC: upper middle-income countries, LMC: lower middle-income countries, LIC: low-income countries

Table A25. Main results for a sensitivity analysis in which own-price elasticities are 10% lower.

Item	Red meat					Processed meat				
	Global	High-income countries	Upper middle-income countries	Lower middle-income countries	Low-income countries	Global	High-income countries	Upper middle-income countries	Lower middle-income countries	Low-income countries
Optimal tax (USD/kg)	0.28	0.94	0.39	0.15	0.02	1.45	4.17	2.41	0.86	0.10
Price before tax (USD/kg)	6.75	4.42	6.05	6.93	8.75	5.74	3.76	5.14	5.89	7.44
Price after tax (USD/kg)	7.03	5.36	6.44	7.08	8.77	7.19	7.93	7.55	6.75	7.54
Price change (%)	4.16	21.34	6.51	2.16	0.23	25.22	111.16	46.85	14.63	1.34
Consumption before tax (g/d)	56.65	94.91	65.98	53.48	25.70	16.52	48.14	25.99	8.88	6.77
Consumption after tax (g/d)	56.99	94.98	66.28	53.99	25.71	14.18	37.36	22.69	8.38	6.70
Consumption change (g/d)	0.34	0.06	0.31	0.52	0.02	-2.34	-10.78	-3.31	-0.51	-0.07
Consumption change (%)	0.60	0.07	0.46	0.96	0.06	-14.14	-22.39	-12.72	-5.71	-1.03
Attributable deaths before tax (thousands)	863.06	167.22	124.08	531.39	34.90	1,533.21	604.53	384.96	484.43	55.69
Attributable deaths after tax (thousands)	869.34	167.19	125.29	536.42	34.93	1,324.78	485.23	327.43	453.77	54.92
Change in attributable deaths (thousands)	6.28	-0.03	1.21	5.04	0.03	-208.43	-119.30	-57.53	-30.66	-0.78
Change in attributable deaths (%)	0.73	-0.02	0.98	0.95	0.08	-13.59	-19.73	-14.94	-6.33	-1.39
Health care-related costs before tax (USD billion)	80.74	44.88	10.00	25.17	0.41	216.53	163.34	33.76	18.45	0.76
Health care-related costs after tax (USD billion)	81.09	44.88	10.10	25.41	0.42	178.32	132.02	28.31	17.05	0.74
Change in health care-related costs (USD billion)	0.35	0.00	0.10	0.25	0.00	-38.20	-31.32	-5.45	-1.41	-0.02
Change in health care-related costs (%)	0.44	0.00	1.03	0.98	0.24	-17.64	-19.18	-16.15	-7.61	-1.98
Tax revenues (USD billion)	70.14	38.56	8.93	22.01	0.38	105.56	73.89	18.62	12.36	0.54

Abbreviations: HIC: high-income countries, UMC: upper middle-income countries, LMC: lower middle-income countries, LIC: low-income countries

Table A26. Main results for a sensitivity analysis in which own-price elasticities are 10% higher.

Item	Red meat					Processed meat				
	Global	High-income countries	Upper middle-income countries	Lower middle-income countries	Low-income countries	Global	High-income countries	Upper middle-income countries	Lower middle-income countries	Low-income countries
Optimal tax (USD/kg)	0.28	0.94	0.39	0.15	0.02	1.45	4.17	2.41	0.86	0.10
Price before tax (USD/kg)	6.75	4.42	6.05	6.93	8.75	5.74	3.76	5.14	5.89	7.44
Price after tax (USD/kg)	7.03	5.36	6.44	7.08	8.77	7.19	7.93	7.55	6.75	7.54
Price change (%)	4.16	21.34	6.51	2.16	0.23	25.22	111.16	46.85	14.63	1.34
Consumption before tax (g/d)	56.65	94.91	65.98	53.48	25.70	16.52	48.14	25.99	8.88	6.77
Consumption after tax (g/d)	56.54	93.30	65.85	53.73	25.70	13.62	34.80	21.90	8.24	6.68
Consumption change (g/d)	-0.12	-1.62	-0.12	0.25	0.00	-2.90	-13.35	-4.10	-0.64	-0.09
Consumption change (%)	-0.20	-1.70	-0.19	0.48	0.02	-17.55	-27.72	-15.75	-7.19	-1.31
Attributable deaths before tax (thousands)	863.06	167.22	124.08	531.39	34.90	1,533.21	604.53	384.96	484.43	55.69
Attributable deaths after tax (thousands)	863.12	164.45	124.39	533.87	34.91	1,273.02	455.61	313.66	445.65	54.71
Change in attributable deaths (thousands)	0.06	-2.77	0.30	2.49	0.01	-260.19	-148.92	-71.30	-38.77	-0.98
Change in attributable deaths (%)	0.01	-1.66	0.25	0.47	0.02	-16.97	-24.63	-18.52	-8.00	-1.77
Health care-related costs before tax (USD billion)	80.74	44.88	10.00	25.17	0.41	216.53	163.34	33.76	18.45	0.76
Health care-related costs after tax (USD billion)	80.07	44.07	10.02	25.29	0.41	168.65	124.02	27.01	16.67	0.74
Change in health care-related costs (USD billion)	-0.67	-0.81	0.02	0.12	0.00	-47.88	-39.32	-6.75	-1.78	-0.02
Change in health care-related costs (%)	-0.83	-1.81	0.23	0.48	0.00	-22.11	-24.07	-19.99	-9.64	-2.51
Tax revenues (USD billion)	69.21	37.83	8.86	21.89	0.37	99.20	68.72	17.71	12.08	0.54

Abbreviations: HIC: high-income countries, UMC: upper middle-income countries, LMC: lower middle-income countries, LIC: low-income countries

Table A27. Main results for a sensitivity analysis in which the price of processed meat is equal to the price of red meat.

Item	Red meat					Processed meat				
	Global	High-income countries	Upper middle-income countries	Lower middle-income countries	Low-income countries	Global	High-income countries	Upper middle-income countries	Lower middle-income countries	Low-income countries
Optimal tax (USD/kg)	0.28	0.94	0.39	0.15	0.02	1.45	4.17	2.41	0.86	0.10
Price before tax (USD/kg)	6.75	4.42	6.05	6.93	8.75	6.75	4.42	6.05	6.93	8.75
Price after tax (USD/kg)	7.03	5.36	6.44	7.08	8.77	8.20	8.59	8.46	7.79	8.85
Price change (%)	4.16	21.34	6.51	2.16	0.23	21.43	94.48	39.82	12.42	1.14
Consumption before tax (g/d)	56.65	94.91	65.98	53.48	25.70	16.52	48.14	25.99	8.88	6.77
Consumption after tax (g/d)	56.52	93.44	65.82	53.67	25.70	14.18	37.25	22.73	8.39	6.70
Consumption change (g/d)	-0.13	-1.47	-0.16	0.19	0.00	-2.34	-10.90	-3.26	-0.50	-0.07
Consumption change (%)	-0.24	-1.55	-0.23	0.36	0.00	-14.18	-22.64	-12.56	-5.59	-0.99
Attributable deaths before tax (thousands)	863.06	167.22	124.08	531.39	34.90	1,533.21	604.53	384.96	484.43	55.69
Attributable deaths after tax (thousands)	862.62	164.69	124.29	533.25	34.90	1,324.13	483.93	327.54	454.28	54.94
Change in attributable deaths (thousands)	-0.44	-2.54	0.20	1.86	0.00	-209.08	-120.60	-57.42	-30.15	-0.75
Change in attributable deaths (%)	-0.05	-1.52	0.16	0.35	0.00	-13.64	-19.95	-14.92	-6.22	-1.34
Health care-related costs before tax (USD billion)	80.74	44.88	10.00	25.17	0.41	216.53	163.34	33.76	18.45	0.76
Health care-related costs after tax (USD billion)	80.11	44.15	10.01	25.26	0.41	177.89	131.56	28.31	17.07	0.74
Change in health care-related costs (USD billion)	-0.63	-0.73	0.02	0.09	0.00	-38.64	-31.78	-5.45	-1.39	-0.01
Change in health care-related costs (%)	-0.77	-1.63	0.15	0.35	0.00	-17.84	-19.46	-16.14	-7.51	-1.85
Tax revenues (USD billion)	69.24	37.89	8.85	21.87	0.37	105.28	73.59	18.62	12.38	0.54

Abbreviations: HIC: high-income countries, UMC: upper middle-income countries, LMC: lower middle-income countries, LIC: low-income countries

Table A28. Main results for a sensitivity analysis in which the price of processed meat is 30% less than the price of red meat.

Item	Red meat					Processed meat				
	Global	High-income countries	Upper middle-income countries	Lower middle-income countries	Low-income countries	Global	High-income countries	Upper middle-income countries	Lower middle-income countries	Low-income countries
Optimal tax (USD/kg)	0.28	0.94	0.39	0.15	0.02	1.45	4.18	2.41	0.86	0.10
Price before tax (USD/kg)	6.75	4.42	6.05	6.93	8.75	4.73	3.09	4.24	4.85	6.13
Price after tax (USD/kg)	7.03	5.36	6.44	7.08	8.77	6.17	7.27	6.64	5.71	6.23
Price change (%)	4.16	21.34	6.51	2.16	0.23	30.60	135.03	56.88	17.75	1.63
Consumption before tax (g/d)	56.65	94.91	65.98	53.48	25.70	16.52	48.14	25.99	8.88	6.77
Consumption after tax (g/d)	57.09	95.04	66.40	54.12	25.72	13.54	34.58	21.71	8.21	6.68
Consumption change (g/d)	0.44	0.13	0.42	0.65	0.02	-2.98	-13.56	-4.29	-0.68	-0.10
Consumption change (%)	0.78	0.13	0.64	1.21	0.09	-18.02	-28.17	-16.48	-7.61	-1.42
Attributable deaths before tax (thousands)	863.06	167.22	124.08	531.39	34.90	1,533.21	604.53	384.96	484.43	55.69
Attributable deaths after tax (thousands)	871.05	167.29	125.58	537.73	34.94	1,265.88	453.02	311.35	443.52	54.63
Change in attributable deaths (thousands)	7.99	0.07	1.49	6.34	0.04	-267.33	-151.51	-73.61	-40.91	-1.07
Change in attributable deaths (%)	0.93	0.04	1.20	1.19	0.11	-17.44	-25.06	-19.12	-8.44	-1.91
Health care-related costs before tax (USD billion)	80.74	44.88	10.00	25.17	0.41	216.53	163.34	33.76	18.45	0.76
Health care-related costs after tax (USD billion)	81.19	44.89	10.13	25.48	0.42	167.80	123.47	26.81	16.58	0.74
Change in health care-related costs (USD billion)	0.45	0.01	0.13	0.31	0.00	-48.73	-39.87	-6.95	-1.87	-0.02
Change in health care-related costs (%)	0.56	0.03	1.27	1.23	0.24	-22.51	-24.41	-20.59	-10.14	-2.77
Tax revenues (USD billion)	70.24	38.58	8.95	22.07	0.38	98.64	68.37	17.57	12.01	0.54

Abbreviations: HIC: high-income countries, UMC: upper middle-income countries, LMC: lower middle-income countries, LIC: low-income countries

Table A29. Main results for a sensitivity analysis in which the high value of the standard deviation of healthcare-related costs were used.

Item	Red meat					Processed meat				
	Global	High-income countries	Upper middle-income countries	Lower middle-income countries	Low-income countries	Global	High-income countries	Upper middle-income countries	Lower middle-income countries	Low-income countries
Optimal tax (USD/kg)	0.38	1.31	0.54	0.20	0.03	1.93	5.63	3.20	1.13	0.13
Price before tax (USD/kg)	6.75	4.42	6.05	6.93	8.75	5.74	3.76	5.14	5.89	7.44
Price after tax (USD/kg)	7.13	5.72	6.59	7.13	8.78	7.66	9.38	8.34	7.02	7.57
Price change (%)	5.66	29.54	8.84	2.84	0.31	33.57	149.91	62.27	19.15	1.76
Consumption before tax (g/d)	56.65	94.91	65.98	53.48	25.70	16.52	48.14	25.99	8.88	6.77
Consumption after tax (g/d)	56.62	93.17	65.91	53.90	25.71	13.39	33.99	21.39	8.17	6.67
Consumption change (g/d)	-0.03	-1.74	-0.06	0.43	0.01	-3.13	-14.15	-4.61	-0.71	-0.10
Consumption change (%)	-0.05	-1.83	-0.10	0.80	0.05	-18.94	-29.39	-17.73	-8.04	-1.51
Attributable deaths before tax (thousands)	863.06	167.22	124.08	531.39	34.90	1,533.21	604.53	384.96	484.43	55.69
Attributable deaths after tax (thousands)	864.87	164.25	124.63	535.56	34.92	1,252.91	446.27	307.38	441.35	54.56
Change in attributable deaths (thousands)	1.81	-2.97	0.54	4.17	0.02	-280.30	-158.27	-77.58	-43.08	-1.13
Change in attributable deaths (%)	0.21	-1.78	0.44	0.78	0.06	-18.28	-26.18	-20.15	-8.89	-2.03
Health care-related costs before tax (USD billion)	109.90	62.31	13.60	33.06	0.54	292.64	221.98	45.20	24.18	0.99
Health care-related costs after tax (USD billion)	108.97	61.06	13.65	33.33	0.55	223.84	165.51	35.49	21.60	0.96
Change in health care-related costs (USD billion)	-0.94	-1.25	0.05	0.27	0.00	-68.80	-56.47	-9.71	-2.58	-0.03
Change in health care-related costs (%)	-0.85	-2.01	0.35	0.80	0.18	-23.51	-25.44	-21.48	-10.65	-2.82
Tax revenues (USD billion)	94.09	52.35	12.05	28.85	0.49	130.56	90.88	23.16	15.63	0.70

Abbreviations: HIC: high-income countries, UMC: upper middle-income countries, LMC: lower middle-income countries, LIC: low-income countries

Table A30. Main results for a sensitivity analysis in which the low value of the standard deviation of healthcare-related costs were used.

Item	Red meat					Processed meat				
	Global	High-income countries	Upper middle-income countries	Lower middle-income countries	Low-income countries	Global	High-income countries	Upper middle-income countries	Lower middle-income countries	Low-income countries
Optimal tax (USD/kg)	0.18	0.58	0.25	0.10	0.01	0.97	2.72	1.62	0.60	0.07
Price before tax (USD/kg)	6.75	4.42	6.05	6.93	8.75	5.74	3.76	5.14	5.89	7.44
Price after tax (USD/kg)	6.93	5.00	6.30	7.03	8.76	6.71	6.48	6.76	6.49	7.51
Price change (%)	2.68	13.15	4.17	1.49	0.16	16.87	72.44	31.45	10.10	0.91
Consumption before tax (g/d)	56.65	94.91	65.98	53.48	25.70	16.52	48.14	25.99	8.88	6.77
Consumption after tax (g/d)	56.87	95.00	66.18	53.79	25.71	14.53	38.79	23.35	8.47	6.72
Consumption change (g/d)	0.22	0.09	0.21	0.31	0.01	-1.99	-9.35	-2.64	-0.42	-0.05
Consumption change (%)	0.38	0.09	0.31	0.59	0.03	-12.02	-19.43	-10.16	-4.71	-0.81
Attributable deaths before tax (thousands)	863.06	167.22	124.08	531.39	34.90	1,533.21	604.53	384.96	484.43	55.69
Attributable deaths after tax (thousands)	867.03	167.23	124.94	534.45	34.91	1,355.03	501.33	336.21	458.95	55.07
Change in attributable deaths (thousands)	3.97	0.00	0.86	3.07	0.01	-178.18	-103.21	-48.75	-25.47	-0.62
Change in attributable deaths (%)	0.46	0.00	0.69	0.58	0.03	-11.62	-17.07	-12.66	-5.26	-1.12
Health care-related costs before tax (USD billion)	51.58	27.45	6.40	17.27	0.28	140.41	104.70	22.32	12.73	0.52
Health care-related costs after tax (USD billion)	51.76	27.47	6.45	17.37	0.29	118.94	87.20	19.17	11.92	0.51
Change in health care-related costs (USD billion)	0.18	0.02	0.05	0.10	0.00	-21.47	-17.50	-3.15	-0.81	-0.01
Change in health care-related costs (%)	0.35	0.09	0.84	0.59	0.35	-15.29	-16.71	-14.12	-6.37	-1.53
Tax revenues (USD billion)	44.87	23.67	5.72	15.06	0.26	71.56	49.70	12.72	8.66	0.38

Abbreviations: HIC: high-income countries, UMC: upper middle-income countries, LMC: lower middle-income countries, LIC: low-income countries

References

1 Murray CJ, Ezzati M, Lopez AD, Rodgers A, Vander Hoorn S. Comparative quantification of health risks: conceptual framework and methodological issues. *Popul Health Metr* 2003; **1**: 1.

2 Lim SS, Vos T, Flaxman AD, *et al.* A comparative risk assessment of burden of disease and injury attributable to 67 risk factors and risk factor clusters in 21 regions, 1990–2010: a systematic analysis for the Global Burden of Disease Study 2010. *The Lancet* 2012; **380**: 2224–60.

3 Scarborough P, Nnoaham KE, Clarke D, Capewell S, Rayner M. Modelling the impact of a healthy diet on cardiovascular disease and cancer mortality. *J Epidemiol Community Health* 2012; **66**: 420–6.

4 Wang X, Lin X, Ouyang YY, *et al.* Red and processed meat consumption and mortality: dose-response meta-analysis of prospective cohort studies. *Public Health Nutr* 2016; **19**: 893–905.

5 Feskens EJM, Sluik D, van Woudenbergh GJ. Meat consumption, diabetes, and its complications. *Curr Diab Rep* 2013; **13**: 298–306.

6 Micha R, Wallace SK, Mozaffarian D. Red and processed meat consumption and risk of incident coronary heart disease, stroke, and diabetes mellitus: a systematic review and meta-analysis. *Circulation* 2010; **121**: 2271–83.

7 Micha R, Michas G, Mozaffarian D. Unprocessed red and processed meats and risk of coronary artery disease and type 2 diabetes--an updated review of the evidence. *Curr Atheroscler Rep* 2012; **14**: 515–24.

8 Chen G-C, Lv D-B, Pang Z, Liu Q-F. Red and processed meat consumption and risk of stroke: a meta-analysis of prospective cohort studies. *Eur J Clin Nutr* 2013; **67**: 91–5.

9 Micha R, Michas G, Mozaffarian D. Unprocessed red and processed meats and risk of coronary artery disease and type 2 diabetes--an updated review of the evidence. *Curr Atheroscler Rep* 2012; **14**: 515–24.

10 Abete I, Romaguera D, Vieira AR, Lopez de Munain A, Norat T. Association between total, processed, red and white meat consumption and all-cause, CVD and IHD mortality: a meta-analysis of cohort studies. *Br J Nutr* 2014; **112**: 762–75.

11 WCRF/AICR. Food, Nutrition, Physical Activity, and the Prevention of Cancer: A Global Perspective. Washington, DC, USA: AICR, 2007.

12 Bouvard V, Loomis D, Guyton KZ, *et al.* Carcinogenicity of consumption of red and processed meat. *Lancet Oncol* 2015; **16**: 1599–600.

13 Chan DSM, Lau R, Aune D, *et al.* Red and processed meat and colorectal cancer incidence: meta-analysis of prospective studies. *PloS One* 2011; **6**: e20456.

14 Sinha R, Cross AJ, Graubard BI, Leitzmann MF, Schatzkin A. Meat intake and mortality: a prospective study of over half a million people. *Arch Intern Med* 2009; **169**: 562–71.

15 Pan A, Sun Q, Bernstein AM, *et al.* Red Meat Consumption and Mortality: Results From 2 Prospective Cohort Studies. *Arch Intern Med* 2012; **172**: 555–63.

16 Cover TM, Thomas JA. Elements of information theory. John Wiley & Sons, 2012.

17 Willett WC, Manson JE, Stampfer MJ, et al. Weight, weight change, and coronary heart disease in women: Risk within the ‘normal’ weight range. *JAMA* 1995; **273**: 461–5.

18 Asia Pacific Cohort Studies Collaboration. Body mass index and cardiovascular disease in the Asia-Pacific Region: an overview of 33 cohorts involving 310 000 participants. *Int J Epidemiol* 2004; **33**: 751–8.

19 Song Y-M, Sung J, Smith GD, Ebrahim S. Body Mass Index and Ischemic and Hemorrhagic Stroke A Prospective Study in Korean Men. *Stroke* 2004; **35**: 831–6.

20 Rexrode KM, Hennekens CH, Willett WC, et al. A prospective study of body mass index, weight change, and risk of stroke in women. *JAMA* 1997; **277**: 1539–45.

21 Calle EE, Rodriguez C, Walker-Thurmond K, Thun MJ. Overweight, obesity, and mortality from cancer in a prospectively studied cohort of U.S. adults. *N Engl J Med* 2003; **348**: 1625–38.

22 Reeves GK, Pirie K, Beral V, *et al.* Cancer incidence and mortality in relation to body mass index in the Million Women Study: cohort study. *BMJ* 2007; **335**: 1134.

23 Parr CL, Batty GD, Lam TH, *et al.* Body-mass index and cancer mortality in the Asia-Pacific Cohort Studies Collaboration: pooled analyses of 424,519 participants. *Lancet Oncol* 2010; **11**: 741–52.

24 Calle EE, Kaaks R. Overweight, obesity and cancer: epidemiological evidence and proposed mechanisms. *Nat Rev Cancer* 2004; **4**: 579–91.

25 Prospective Studies Collaboration, Whitlock G, Lewington S, *et al.* Body-mass index and cause-specific mortality in 900 000 adults: collaborative analyses of 57 prospective studies. *Lancet* 2009; **373**: 1083–96.

26 Willett WC, Dietz WH, Colditz GA. Guidelines for healthy weight. *N Engl J Med* 1999; **341**: 427–34.

27 Chiolero A, Kaufman JS. Metabolic mediators of body-mass index and cardiovascular risk. *The Lancet* 2014; **383**: 2042.

28 Yusuf S, Hawken S, Ounpuu S, *et al.* Effect of potentially modifiable risk factors associated with myocardial infarction in 52 countries (the INTERHEART study): case-control study. *Lancet* 2004; **364**: 937–52.

29 Khaw K-T, Wareham N, Bingham S, Welch A, Luben R, Day N. Combined Impact of Health Behaviours and Mortality in Men and Women: The EPIC-Norfolk Prospective Population Study. *PLoS Med* 2008; **5**: e12.

30 Dam RM van, Li T, Spiegelman D, Franco OH, Hu FB. Combined Impact of Lifestyle Factors on Mortality: Prospective Cohort Study in US Women. *BMJ* 2008; **337**: 742–5.

31 Huxley RR, Ansary-Moghaddam A, Clifton P, Czernichow S, Parr CL, Woodward M. The impact of dietary and lifestyle risk factors on risk of colorectal cancer: a quantitative overview of the epidemiological evidence. *Int J Cancer J Int Cancer* 2009; **125**: 171–80.

32 Nechuta SJ, Shu X-O, Li H-L, *et al.* Combined impact of lifestyle-related factors on total and cause-specific mortality among Chinese women: prospective cohort study. *PLoS Med* 2010; **7**. DOI:10.1371/journal.pmed.1000339.

33 Berrington de Gonzalez A, Hartge P, Cerhan JR, *et al.* Body-Mass Index and Mortality among 1.46 Million White Adults. *N Engl J Med* 2010; **363**: 2211–9.

34 Springmann M, Godfray HCJ, Rayner M, Scarborough P. Analysis and valuation of the health and climate change cobenefits of dietary change. *Proc Natl Acad Sci* 2016; **113**: 4146–51.

35 Leal J, Luengo-Fernández R, Gray A, Petersen S, Rayner M. Economic burden of cardiovascular diseases in the enlarged European Union. *Eur Heart J* 2006; **27**: 1610–9.

36 Bloom DE, Cafiero E, Jané-Llopis E, *et al.* The global economic burden of noncommunicable diseases. Geneva: World Economic Forum, 2011.

37 Nichols M, Townsend N, Scarborough P, Rayner M. European cardiovascular disease statistics. 2012.

38 Luengo-Fernandez R, Leal J, Gray A, Sullivan R. Economic burden of cancer across the European Union: a population-based cost analysis. *Lancet Oncol* 2013; **14**: 1165–74.

39 Zhang P, Zhang X, Brown J, *et al.* Global healthcare expenditure on diabetes for 2010 and 2030. *Diabetes Res Clin Pract* 2010; **87**: 293–301.

40 Köster I, Huppertz E, Hauner H, Schubert I. Direct costs of diabetes mellitus in Germany-CoDiM 2000-2007. *Exp Clin Endocrinol Diabetes Off J Ger Soc Endocrinol Ger Diabetes Assoc* 2011; **119**: 377–385.

41 American Diabetes Association. Economic costs of diabetes in the US in 2012. *Diabetes Care* 2013; **36**: 1033–1046.

42 Robinson S, Mason-D'Croz D, Islam S, *et al.* The International Model for Policy Analysis of Agricultural Commodities and Trade (IMPACT) -- Model description for version 3. 2015; published online Oct.

43 Evenson RE, Rosengrant MW. Productivity Projections for Commodity Marketing Modeling. Paper presented at the final workshop of the International Cooperative Research Project on 'Projections and Policy Implications of Medium and Long-Term Rice Supply and Demand', organized by IFPRI, IRRI, and CCER, Beijing, China, April 23-26, 1995. 1995.

44 Evenson RE, Pray C, Rosengrant MW. Agricultural Research and Productivity Growth in India. IFPRI Research Report No. 109. 1999.

45 Hoogenboom G, Jones JW, Wilkens PW, *et al.* Decision Support System for Agrotechnology Transfer (DSSAT). ver. 4.5 [CD-ROM]. *Univ Hawaii Honol Hawaii* 2012.

46 Jones JW, Hoogenboom G, Porter CH, *et al.* The DSSAT cropping system model. *Eur J Agron* 2003; **18**: 235–265.

47 USDA. Commodity and Food Elasticities. Retrieved from http://www.ers.usda.gov/Data/Elasticities/. 1998.

48 Green R, Cornelsen L, Dangour AD, *et al.* The effect of rising food prices on food consumption: systematic review with meta-regression. *BMJ* 2013; **346**. http://www.bmj.com/content/346/bmj.f3703.full (accessed Jan 17, 2015).

49 GAMS. General Algebraic Modeling System (GAMS). GAMS, Washington, D.C. Retrieved from www.gams.com. 2012.

50 World Bank. Manufactures Unit Value Index. 2000. http://data.worldbank.org/data-catalog/MUV-index.

51 World Bank. Prospects Commodity Markets. 2012. http://go.worldbank.org/4ROCCIEQ50.

52 OECD-AMAD. Agricultural market Access Data Base. Retrieved from www.oecd.org/site/amad in 2013. 2010.

53 Narayanan BG, Walmsley TL. Global Trade, Assistance, and Production: The GTAP 7 Data Base, Center for Global Trade Analysis, Purdue University. Available online at: http://www.gtap.agecon.purdue.edu/databases/v7/v7_doco.asp. 2008.

54 International Trade Center. User Guide - Market Access Map: Making Tariffs and Market Access Barriers Transparent. Market Analysis Section, Division of Product and Market Development, International Trade Center, Geneva, December. 2006.

55 Boumellassa H, Laborde D, Mitaritonna C. A picture of tariff protection across the world in 2004: MAcMap-HS6, version 2. Intl Food Policy Res Inst, 2009.

56 OECD. Agricultural Policy Monitoring and Evaluation 2014: OECD Countries. 2014.

57 Food and Agriculture Organization of the United Nations. Food balance sheets: a handbook. Rome, 2001.

58 Hawkesworth S, Dangour AD, Johnston D, *et al.* Feeding the world healthily: the challenge of measuring the effects of agriculture on health. *Philos Trans R Soc Lond B Biol Sci* 2010; **365**: 3083–97.

59 Kearney J. Food consumption trends and drivers. *Philos Trans R Soc B Biol Sci* 2010; **365**: 2793–807.

60 Gustavsson J, Cederberg C, Sonesson U, Van Otterdijk R, Meybeck A. Global food losses and food waste: extent, causes and prevention. FAO Rome, 2011 http://www.sidalc.net/cgi-bin/wxis.exe/?IsisScript=SIBE01.xis&method=post&formato=2&cantidad=1&expresion=mfn=028275 (accessed Oct 31, 2014).

61 Gerber PJ, Steinfeld H, Henderson B, *et al.* Tackling climate change through livestock: a global assessment of emissions and mitigation opportunities. Rome: FAO, 2013.

62 Tilman D, Clark M. Global diets link environmental sustainability and human health. *Nature* 2014; **515**: 518–22.

63 Forouzanfar MH, Alexander L, Anderson HR, *et al.* Global, regional, and national comparative risk assessment of 79 behavioural, environmental and occupational, and metabolic risks or clusters of risks in 188 countries, 1990–2013: a systematic analysis for the Global Burden of Disease Study 2013. *The Lancet* 2015; **386**: 2287–323.
